# Metabolomic and lipidomic profile in men with obstructive sleep apnoea: implications for diagnosis and biomarkers of cardiovascular risk

**DOI:** 10.1038/s41598-018-29727-6

**Published:** 2018-07-26

**Authors:** Adriana Lebkuchen, Valdemir M. Carvalho, Gabriela Venturini, Jéssica S. Salgueiro, Lunara S. Freitas, Alessandra Dellavance, Franco C. Martins, Geraldo Lorenzi-Filho, Karina H. M. Cardozo, Luciano F. Drager

**Affiliations:** 1 Unidade de Hipertensão, Instituto do Coração, Faculdade de Medicina, Universidade de São Paulo, São Paulo, SP Brazil; 2 Grupo Fleury, São Paulo, SP Brazil; 3 Laboratório de Genética e Cardiologia Molecular, Instituto do Coração, Faculdade de Medicina, Universidade de São Paulo, São Paulo, SP Brazil; 4 Laboratório do sono, Instituto do Coração, Faculdade de Medicina, Universidade de São Paulo, São Paulo, SP Brazil

## Abstract

The use of metabolomic and lipidomic strategies for selecting potential biomarkers for obstructive sleep apnoea (OSA) has been little explored. We examined adult male patients with OSA (defined by an apnoea-hypopnoea index ≥15 events/hour), as well as age-, gender-, and fat-composition-matched volunteers without OSA. All subjects were subjected to clinical evaluation, sleep questionnaires for detecting the risk of OSA (Berlin and NoSAS score), metabolomic analysis by gas chromatography coupled to mass spectrometry and lipidomic analysis with liquid chromatography followed by detection by MALDI-MS. This study included 37 patients with OSA and 16 controls. From the 6 metabolites and 22 lipids initially selected, those with the best association with OSA were glutamic acid, deoxy sugar and arachidonic acid (metabolites), and glycerophosphoethanolamines, sphingomyelin and lyso-phosphocholines (lipids). For the questionnaires, the NoSAS score performed best with screening for OSA (area under the curve [AUC] = 0.724, *p* = 0.003). The combination of the NoSAS score with metabolites or lipids resulted in an AUC for detecting OSA of 0.911 and 0.951, respectively. In conclusion, metabolomic and lipidomic strategies suggested potential early biomarkers in OSA that could also be helpful in screening for this sleep disorder beyond traditional questionnaires.

## Introduction

Obstructive sleep apnoea (OSA) is a clinical condition characterized by complete or partial collapse of the upper airway during sleep, which produces intrathoracic pressure reduction, intermittent hypoxia and sleep fragmentation^1–4^. OSA has been shown to be highly prevalent in men, in the obese and in patients at risk of cardiovascular disease (CVD)^5–9^. More importantly, OSA, an underdiagnosed condition in clinical practice^10^, is independently associated with higher rates of cardiovascular morbidity and mortality^11^. It is conceivable that the increased cardiovascular risk attributed to OSA is partially mediated by metabolic dysfunction in these patients^12^. Moreover, recent translational evidence suggests that early OSA detection is crucial because the impact of OSA on the cardiovascular system may not be reversible if treatment is delayed^3^. Indeed, the results from one of the largest randomized trials addressing the effect of OSA treatment in patients with established CVD were neutral^13^. Therefore, it is necessary to explore early biomarkers of OSA, as well as potential metabolic pathways by which OSA increases cardiovascular risk.

Metabolomics, which includes lipidomic analysis, is an “omic” strategy that studies the partial or global profile of metabolites from a subject with high-sensitivity and high-throughput to characterize changes in low-molecular-weight metabolites^14,15^. The analytical metabolomic platforms most applied include liquid and gas chromatography coupled to mass spectrometry by untargeted, semi-targeted and targeted strategy, mass spectrometry and nuclear magnetic resonance^14–17^.

To date, a few studies have explored the detailed metabolic profile in OSA using “omic” strategies^18–21^. However, these studies failed to control for obesity parameters (including body fat composition) or failed to exclude comorbidities and medications, which may influence the results. We utilized a combination of metabolomic analytical platforms, such as ultra-performance liquid chromatography coupled to tandem quadrupole mass spectrometry (UPLC-MS/MS) and gas chromatography coupled to quadrupole MS (GC-MS), to investigate the changes in the metabolic pathway. In addition, off-line UPLC with matrix-assisted laser desorption/ionization-mass spectrometry (MALDI-MS) detection was used to screen lipid profiles in young patients with OSA. Our aims were twofold: (1) to investigate the metabolic and lipidomic profile differences in participants with and without OSA, exploring the potential pathways involved in the cardiovascular risk in OSA; and (2) to identify the potential value of selected biomarkers, in addition to sleep questionnaires, in screening for OSA. We propose two hypotheses: (1) there are significant differences in the metabolomic and lipidomic profiles of OSA patients compared to the matched control group, and some of these metabolites are directly related to intermittent hypoxia, a hallmark of OSA; and (2) the use of some of these metabolites may add value to the diagnosis of OSA when using sleep questionnaires.

## Results

### Subject characteristics

**Figure 1:**
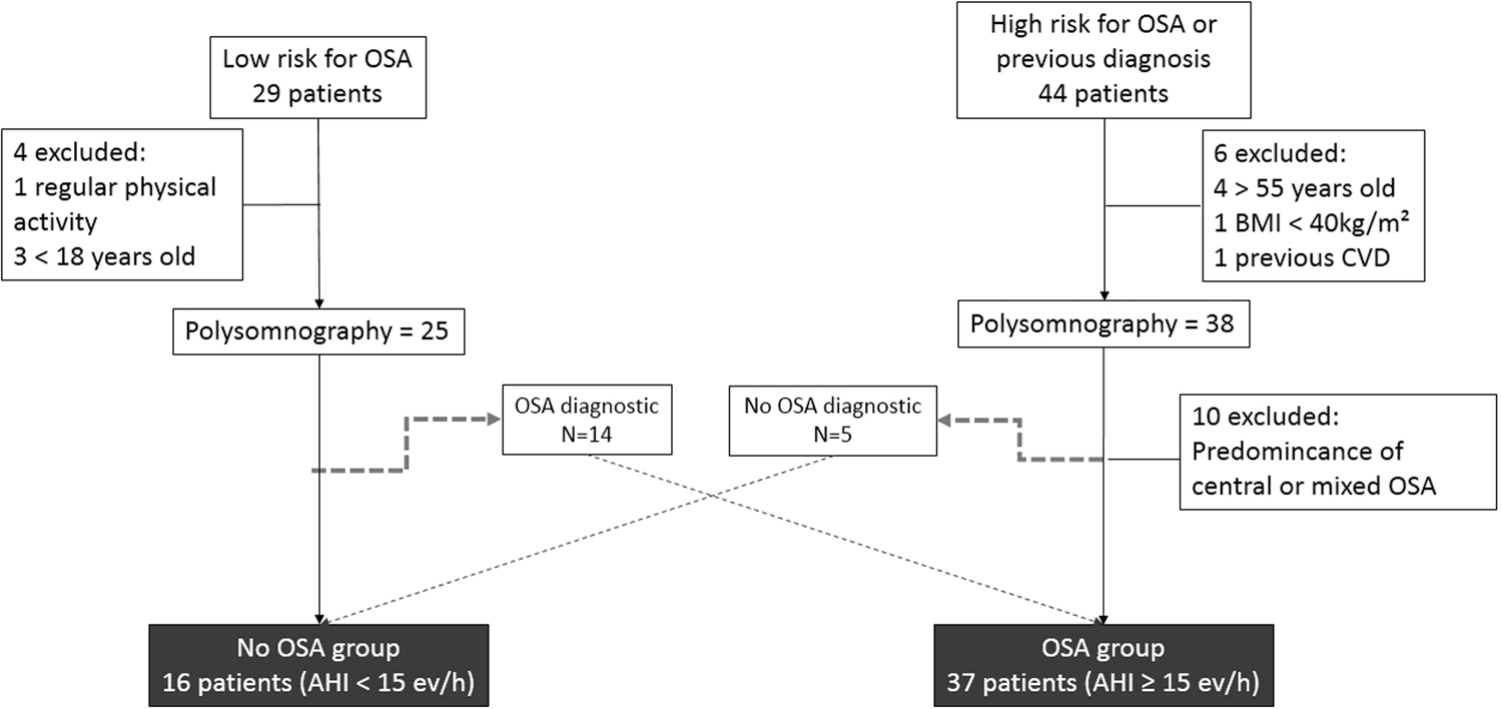
Flow chart of subject selection. Exclusion subject discrimination, crosstalk between groups and study group final composition.

In total, 73 subjects (20 with low risk and 53 with high risk of OSA) were selected. A detailed recruitment process is described in the methods section. After exclusions due to refusals, morbid obesity, previous CVD and predominant central sleep apnoea, only 53 participants were included in the study: 37 with OSA and 16 without OSA (Fig. 1).

Table 1 shows the basic characteristics of the total sample group according to OSA status. Overall, our study comprised young obese adults. Sleep parameters (as expected) were significantly different between the groups: while the AHI and the percentage of total sleep time with oxygen saturation <90% were higher in the OSA group, minimum oxygen saturation was lower in OSA patients. Diastolic blood pressure was higher in patients with OSA.

**Table 1:** Subject characteristics according to study group with respective average, standard deviation, p value and AUC for each variable.

	Total (n = 53)	No OSA (n = 16)	OSA (n = 37)	*p* value	CI dif. 95%	AUC (CI 95%)
**General data**						
Age (years)	38 ± 6	36 ± 6	39.2 ± 7	0.151	[−7.14; 0.81]	0.626 (0.786–0.466)
Neck circumference (cm)	42.0 ± 1.7	40.9 ± 2.4	42.5 ± 2.1	0.027	[−2.92; −0.07]	0.693 (0.869–0.518)
Waist circumference (cm)	105.8 ± 7.6	104.9 ± 9.5	106.1 ± 9.7	0.405	[−7.01; 4.64]	0.573 (0.746–0.401)
Hip circumference (cm)	108.6 ± 5.8	109.5 ± 9.1	108.2 ± 8.5	0.946	[−4.16; 6.81]	0.507 (0.684–0.33)
Systolic blood pressure (mm Hg)	125.4 ± 7.7	123.3 ± 8.1	126.3 ± 10.7	0.204	[−8.48; 2.40]	0.611 (0.781–0.442)
Diastolic blood pressure (mm Hg)	83.8 ± 7.6	79.6 ± 7.5	85.7 ± 10.7	0.047	[−11.24; −0.86]	0.674 (0.828–0.520)
**Sleep study**						
AHI (ev/h)	29.5 ± 18.0	7.4 ± 3.9	39.1 ± 23.5	<0.001	[−39.75; −23.61]	1.000 (1.000–1.000)
Minimum saturation (%)	82.4 ± 6.0	88.8 ± 2.8	79.7 ± 7.5	<0.001	[6.27; 11.95]	0.907 (0.991–0.823)
TST O2 sat <90% (min)	5.2 ± 6.3	0.1 ± 0.1	7.4 ± 11.9	<0.001	[−11.27; −3.30]	0.930 (0.998–0.862)
**Body composition**						
BMI (kg/m²)	30.5 ± 2.7	30.3 ± 3.5	30.6 ± 3.4	0.399	[−2.46; 1.79]	0.574 (0.748–0.401)
Muscle mass (kg)	36.8 ± 3.4	36.9 ± 2.7	36.7 ± 5.1	0.727	[−2.04; 2.31]	0.531 (0.689–0.374)
Fat mass (kg)	28.6 ± 7.2	27.2 ± 11.5	29.2 ± 8.2	0.178	[−8.58; 4.57]	0.618 (0.798–0.439)
% Fat	30.2 ± 5.4	28.7 ± 7.4	30.8 ± 5.9	0.237	[−6.42; 2.18]	0.604 (0.783–0.424)
VFA (cm²)	120.0 ± 29.0	115.2 ± 41.3	122.1 ± 34.4	0.314	[−31.21; 17.40]	0.589 (0.769–0.408)
BMR (kcal)	1767.3 ± 124.2	1770.7 ± 100.8	1765.9 ± 184.1	0.713	[−74.39; 83.98]	0.533 (0.691–0.375)

AHI: apnoea-hypopnoea index; AUC: area under the curve; BMI: body mass index; BMR: basal metabolic rate; CI: confidence interval; OSA: obstructive sleep apnoea; TST O2 sat <90%: total saturation time with oxygen saturation lower than 90%; VFA: visceral fat area.

### Untargeted metabolomic analysis

**Figure 2:**
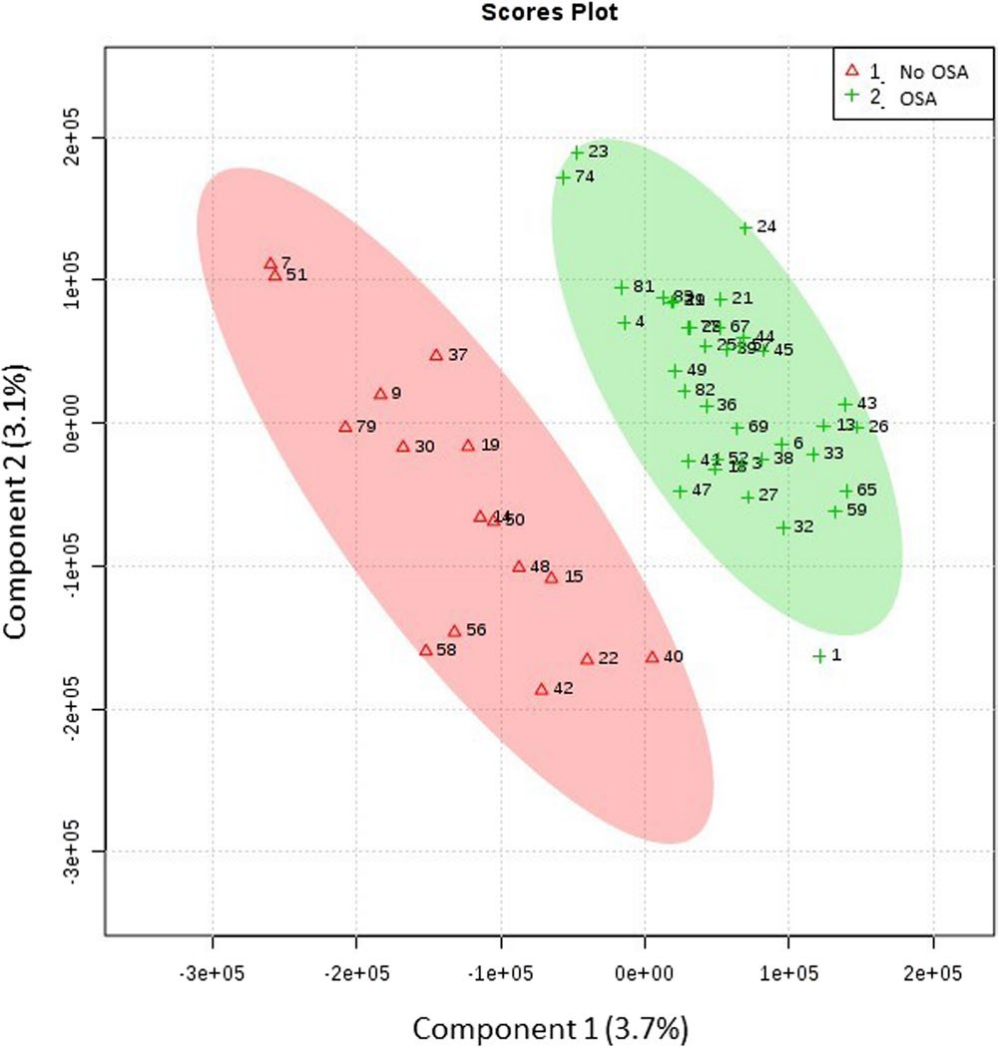
Supervised partial least-squares discriminant analysis (PLS-DA) from GC-MS analysis. Groups are shown in red without OSA and in green with OSA.

The supervised partial least-squares discriminant analysis (PLS-DA) revealed an appropriate model to explain and predict variance between groups (R^2^ = 0.9933 and Q^2^ = 0.6551, accuracy: 0.7067; Fig. 2). Among 152 metabolites (Supplemental Table 1), we found significant differences in six metabolites: Four (also related to glucose and inflammatory pathways) were increased in OSA patients (deoxy sugar; 2,6-diphenyl-1,7-dihydrodipyrrolo[2,3-b:3′,2′-e] pyridine; 9-hexadecenoic acid (Z) and arachidonic acid). In contrast, two showed lower levels in OSA patients: 5,5′-biphthalide and L-glutamine. Pearson’s correlations between metabolites and OSA severity parameters are presented in Supplemental Table 2. A positive correlation with AHI was observed in glutamine and deoxy sugar, whereas arachidonic acid had a negative correlation with AHI.

### Untargeted lipidomic analysis

**Figure 3:**
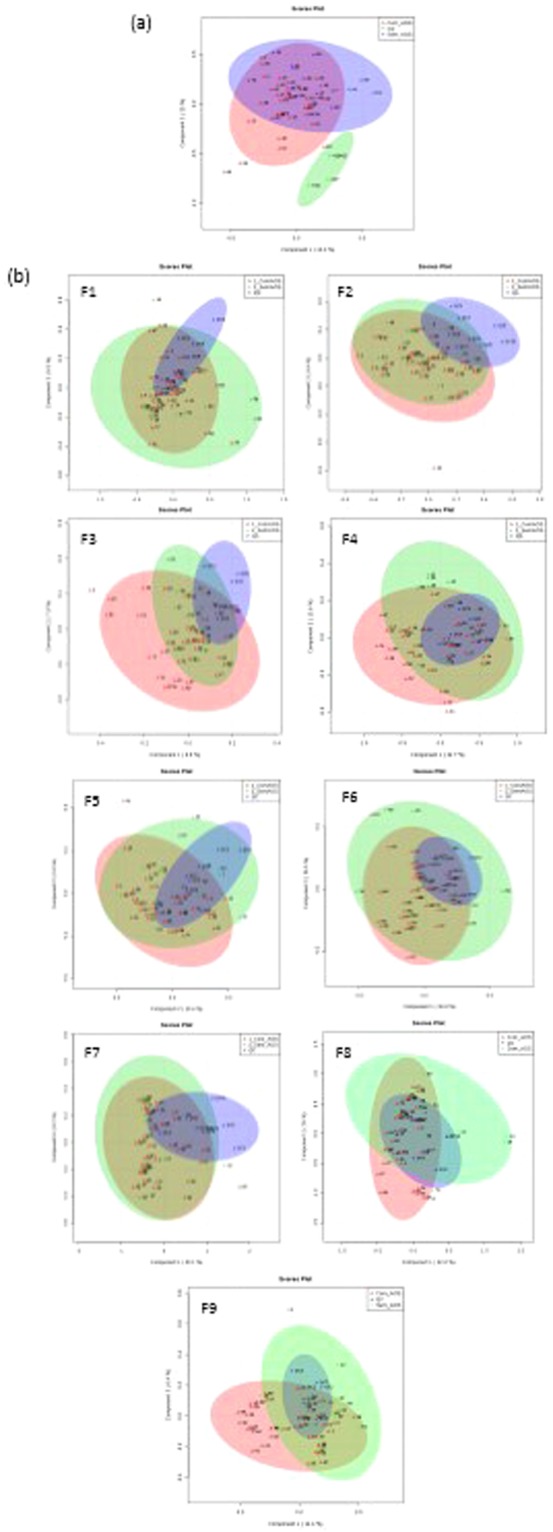
Supervised partial least-squares discriminant analysis (PLS-DA) from untargeted lipidomic analysis. Red: Participants without OSA; Green: Patients with OSA; Blue: Quality controls. (**a**) All samples together before fraction collect and (**b**) samples after each fraction was collected.

The PLS-DA reported in Fig. 3 shows the performance of a quality control (QC) sample before (a) and after (b) fractionation of different lipids classes by off-line UPLC. QC samples were predicted by the model and appeared clustered together in the score plot thus proving the robustness of the methodology^14^. A total of 22 lipids from different lipid classes were significantly different in patients with and without OSA (Table 2) according to off-line UPLC separation and MALDI-MS analysis. The full features with probable lipid identification are listed in Supplementary Table 3. From the 22 significant lipids, glycerophosphoethanolamines (PE), monoacylglycerophosphocholines (lyso-phosphocholines) (LPC), and sphingomyelin (SM) classes were up-regulated in OSA patients compared to patients without OSA. In contrast, diacylglycerols (DAG), glycerophosphocholines (PC), and glycerophosphates (PA) were down-regulated in patients with OSA. Pearson’s correlations between lipids and OSA severity parameters are presented in Supplemental Table 4. A negative correlation with AHI was observed for PA and PC, whereas PE and SM were positively correlated with AHI.

**Table 2:** Statistically significant lipids according to study groups. Average intensity value normalized (x1000) to each lipid is shown with standard deviation, p value, VIP score and AUC.

	Total (n = 53)	No OSA (n = 16)	OSA (n = 37)	*p* value	VIP	CI dif. 95%	AUC (CI 95%)
DAG(45:8)	1.5 ± 0.3	1.7 ± 0.5	1.4 ± 0.3	0.022	2.016	[−0.14; 5.23]	0.699 (0.875–0.523)
Cer(d18:1/24:4)	0.3 ± 0.1	0.2 ± 0.1	0.3 ± 0.1	0.044	2.540	[−5.63; 0.13]	0.676 (0.836–0.515)
PA(35:2)	1.3 ± 0.2	1.4 ± 0.2	1.3 ± 0.2	0.064	3.780	[−0.05; 2.42]	0.662 (0.828–0.497)
PE(36:5)	2.3 ± 0.8	1.8 ± 0.7	2.5 ± 1.0	0.023	3.426	[−11.69; −1.50]	0.698 (0.850–0.545)
PE(35:1)	2.4 ± 1.3	1.5 ± 0.9	2.2 ± 1.1	0.018	3.516	[−12.92; −1.30]	0.704 (0.856–0.553)
PE(38:6)	2.2 ± 1.0	1.7 ± 1.1	2.6 ± 1.5	0.032	3.893	[−16.44; −1.46]	0.688 (0.842–0.535)
PE(38:5)	2.6 ± 1.3	1.6 ± 1.3	2.7 ± 1.6	0.019	4.301	[−18.93; −2.04]	0.705 (0.858–0.552)
PE(37:3)	2.0 ± 0.9	1.7 ± 0.9	2.5 ± 1.3	0.018	3.768	[−14.44; −1.74]	0.704 (0.857–0.552)
PE(37:2)	2.4 ± 1.2	1.9 ± 1.3	2.9 ± 1.7	0.024	4.180	[−18.77; −1.78]	0.696 (0.850–0.542)
PE(39:4)	1.5 ± 0.6	1.2 ± 0.6	1.6 ± 0.07	0.040	2.749	[−8.25; −0.67]	0.679 (0.838–0.52)
PC(33:3)	7.8 ± 2.4	8.8 ± 3.3	7.4 ± 2.9	0.030	3.761	[−4.92; 34.14]	0.694 (0.850–0.539)
PC(36:4)	15.4 ± 2.3	16.4 ± 3.6	14.9 ± 3.4	0.021	3.548	[−6.86; 36.31]	0.706 (0.860–0.551)
PC(35:5)	10.2 ± 1.6	10.9 ± 2.4	9.8 ± 2.3	0.019	2.974	[−4.39; 24.70]	0.709 (0.865–0.554)
PC(34:0)	8.4 ± 2.1	9.3 ± 2.9	8.0 ± 2.6	0.022	3.505	[−4.41; 30.31]	0.704 (0.856–0.551)
PC(37:7)	8.1 ± 1.3	8.5 ± 1.9	7.9 ± 1.9	0.074	2.055	[−5.24; 17.79]	0.661 (0.817–0.505)
PC(37:6)	10.1 ± 1.6	10.8 ± 2.4	9.8 ± 2.3	0.030	2.883	[−4.86; 24.70]	0.694 (0.852–0.537)
PC(36:1)	9.4 ± 1.6	10.0 ± 2.0	9.1 ± 2.2	0.037	2.730	[−3.62; 21.58]	0.687 (0.833–0.541)
SM(d18:1/12:0)	2.8 ± 0.7	2.4 ± 1.0	3.0 ± 0.8	0.025	2.449	[−12.35; −1.06]	0.694 (0.860–0.529)
SM(d18:1/24:0)	2.9 ± 0.5	2.5 ± 0.9	3.1 ± 0.5	0.038	2.304	[−10.78; −0.46]	0.681 (0.855–0.507)
SM(d18:1/26:1(17Z))	3.1 ± 0.4	2.8 ± 1.0	3.3 ± 0.4	0.048	2.157	[−10.50; 0.16]	0.666 (0.840–0.491)
LPC(27:1)	2.1 ± 0.4	1.8 ± 0.5	2.2 ± 0.5	0.008	2.955	[−7.41; −1.27]	0.730 (0.882–0.578)
LPC(27:0)	2.7 ± 0.8	2.4 ± 0.9	2.9 ± 0.9	0.01	2.837	[−10.90; −0.10]	0.671 (0.828–0.513)

AUC: area under the curve; CI: confidence interval; DAG: diacylglycerol; Cer: ceramide; LPC: monoacylglycerophosphocholines (lyso-phosphocholines); PA: glycerophosphates; PC: glycerophosphocholines; PE: glycerophosphoethanolamines; OSA: obstructive sleep apnoea; SM: sphingomyelin; VIP: variable’s importance in the PLS-DA model.

### Targeted metabolomic analysis

Biochemical indices, salivary cortisol, urinary catecholamine, adiponectin, leptin, IL-6 and free fatty acids were not significantly different between groups (Table 3). A plasma amino acids experiment quantified 24 amino acids (Table 4) and resulted in lower levels of glutamic acid (*p* = 0.023) in patients with OSA when compared with patients without OSA. No differences were observed in the remaining amino acids.

**Table 3:** Targeted metabolomics results to clinical evaluation study group.

Clinic Profile	Total (n = 53)	No OSA (n = 16)	OSA (n = 37)	*p* value	CI dif. 95%	AUC (CI 95%)
Glucose (mg/dL)	91.7 ± 5.4	94.6 ± 9.6	90.4 ± 5.4	0.159	[−1.18; 9.56]	0.623 (0.804–0.442)
CRP (mg/dL)	0.31 ± 0.18	0.27 ± 0.21	0.32 ± 0.33	0.907	[−0.20; 0.10]	0.511 (0.682–0.340)
Cholesterol (mg/dL)	190.0 ± 22.3	184.1 ± 32.4	192.6 ± 26.1	0.265	[−27.53; 10.35]	0.598 (0.784–0.412)
HDL cholesterol (mg/dL)	40.3 ± 7.5	40.1 ± 7.8	40.3 ± 11.3	0.756	[−5.72; 5.14]	0.528 (0.697–0.359)
VLDL cholesterol (mg/dL)	29.9 ± 11.2	28.1 ± 17.8	30.7 ± 14.0	0.309	[−12.92; 7.77]	0.590 (0.768–0.411)
LDL cholesterol (mg/dL)	119.9 ± 21.7	115.9 ± 24.2	121.8 ± 28.3	0.318	[−21.42; 9.65]	0.588 (0.759–0.416)
Triglycerides (mg/dL)	149.4 ± 56.1	140.2 ± 88.5	153.3 ± 70.0	0.304	[−64.62; 38.47]	0.590 (0.768–0.413)
TSH (mU/I)	1.9 ± 0.7	2.1 ± 1.1	1.9 ± 0.8	0.485	[−0.43; 0.89]	0.562 (0.750–0.373)
Insulin (mU/I)	15.2 ± 6.4	14.5 ± 7.9	15.5 ± 8.8	0.801	[−5.99; 3.96]	0.523 (0.706–0.340)
HOMA-IR	3.4 ± 1.5	3.4 ± 1.9	3.4 ± 2.0	0.946	[−1.24; 1.18]	0.507 (0.693–0.321)
Free fatty acid (nmol)	0.6 ± 0.2	0.6 ± 0.2	0.6 ± 0.3	0.839	[−0.18; 0.11]	0.519 (0.702–0.335)
Adiponectin (ug/mL)	682.7 ± 334.8	718.9 ± 400.8	667.1 ± 373.4	0.498	[−189.38; 293]	0.560 (0.746–0.374)
Leptin (ng/mL)	4.6 ± 5.0	6.3 ± 10.7	3.9 ± 7.1	0.140	[−3.63; 8.47]	0.628 (0.794–0.463)
Interleukin-6 (ng/mL)	69.8 ± 9.1	72.1 ± 13.3	68.8 ± 10.9	0.361	[−4.54; 11.06]	0.581 (0.772–0.390)
Cortisol Morning (ng/dL)	91.7 ± 5.2	239.5 ± 119.8	256.4 ± 185.5	0.822	[−103.72; 69.89]	0.521 (0.687–0.355)
Cortisol Afternoon (ng/dL)	91.7 ± 5.19	67.9 ± 40.5	76.8 ± 70.7	0.717	[−40.19; 22.33]	0.533 (0.696–0.370)
Cortisol Night (ng/dL)	91.7 ± 5.20	33.5 ± 40.6	22.8 ± 17.2	0.766	[−11.59; 32.89]	0.473 (0.649–0.297)
Noradrenaline (ug/24 h)	91.7 ± 5.2	33.3 ± 18.2	40.1 ± 20.8	0.277	[−18.32; 4.86]	0.596 (0.771–0.422)
Adrenaline (ug/24 h)	91.7 ± 5.2	5.5 ± 5.8	6.4 ± 5.8	0.289	[−4.46; 2.61]	0.590 (0.752–0.427)
Dopamine (ug/24 h)	91.7 ± 5.2	144.9 ± 81.1	180.4 ± 84.3	0.186	[−85.74; 14.65]	0.617 (0.794–0.439)

Averages are shown with standard deviation, p value, VIP score and AUC for each metabolite.

AUC: area under the curve; CI: confidence interval; CRP: C-reactive protein; HDL: high density lipoprotein; LDL: low density lipoprotein; OSA: obstructive sleep apnoea; TSH: thyroid-stimulating hormone; VLDL: very-low-density lipoprotein.

**Table 4:** Targeted metabolomic results to amino acid analysis.

Amino acid (µmol/L)	Total (n = 53)	No OSA (n = 16)	OSA (n = 37)	*p* value	CI dif. 95%	AUC (CI 95%)
Aspartic acid	4.9 ± 1.0	4.7 ± 1.0	5.1 ± 1.5	0.467	[−1.05; 0.40]	0.564 (0.734–0.395)
Glutamic acid	71.8 ± 20.7	83.5 ± 22.5	66.7 ± 24.5	0.023*	[2.70; 30.92]	0.698 (0.847–0.549)
Alanine	337.9 ± 50.4	333.8 ± 61.1	339.7 ± 71.2	0.723	[−45.07; 33.25]	0.532 (0.710–0.354)
Allo-isoleucine	3.6 ± 1.2	3.9 ± 1.4	3.4 ± 1.4	0.378	[−0.42; 1.30]	0.578 (0.750–0.405)
Arginine	61.8 ± 10.2	57.6 ± 10.6	63.6 ± 14.1	0.119	[−13.15; 1.10]	0.637 (0.803–0.471)
Asparagine	36.0 ± 5.8	36.2 ± 4.8	36 ± 8.1	0.691	[−3.34; 3.90]	0.535 (0.693–0.378)
Citrulline	29.7 ± 5.2	30.0 ± 5.3	29.6 ± 7.0	0.642	[−3.17; 3.93]	0.541 (0.703–0.380)
Phenylalanine	45.8 ± 17.2	44.6 ± 16.5	46.3 ± 20.9	0.846	[−12.61; 9.13]	0.518 (0.678–0.358)
Glycine	210.2 ± 34.7	208.4 ± 52.2	211.1 ± 41.4	0.574	[−33.11; 27.71]	0.550 (0.734–0.366)
Glutamine	618.9 ± 73.1	585.2 ± 61.9	633.5 ± 93.7	0.067	[−92.43; −4.32]	0.660 (0.812–0.509)
Hydroxyproline	11.2 ± 3.4	10.4 ± 4.0	11.6 ± 4.3	0.318	[−3.71; 1.27]	0.588 (0.762–0.414)
Histidine	72.5 ± 6.4	69.8 ± 10.5	73.6 ± 6.8	0.215	[−9.82; 2.10]	0.609 (0.794–0.424)
Isoleucine	85.1 ± 11.9	84.8 ± 15.4	85.3 ± 16.5	0.757	[−10.17; 9.06]	0.528 (0.710–0.346)
Leucine	152.2 ± 18.9	152.9 ± 24.0	152.0 ± 24.0	0.946	[−13.82; 15.64]	0.507 (0.695–0.319)
Lysine	178.1 ± 26.2	173.7 ± 34.2	180.0 ± 31.4	0.342	[−26.80; 14.24]	0.584 (0.757–0.411)
Methionine	25.9 ± 2.3	25.1 ± 3.1	26.4 ± 2.9	0.337	[−3.14; 0.61]	0.584 (0.754–0.415)
Ornithine	80.8 ± 15.2	81.5 ± 18.8	80.5 ± 19.5	0.794	[−10.59; 12.69]	0.524 (0.707–0.340)
Proline	204.0 ± 15.2	200.1 ± 48.8	205.7 ± 39.0	0.611	[−34.11; 22.86]	0.546 (0.732–0.359)
Serine	96.6 ± 12.9	100.6 ± 11.2	94.8 ± 18.2	0.104	[−2.46; 14.05]	0.643 (0.794–0.492)
Taurine	28.6 ± 20.0	28.1 ± 25.4	28.8 ± 21.6	0.969	[−15.71; 14.23]	0.496 (0.679–0.312)
Tyrosine	55.3 ± 14.0	52.4 ± 19.1	56.5 ± 17.1	0.584	[−15.41; 7.35]	0.549 (0.728–0.370)
Threonine	92.8 ± 12.9	91.2 ± 12.1	93.6 ± 17.5	0.786	[−10.82; 6.04]	0.476 (0.639–0.312)
Thriptophan	71.33 ± 6.8	71.5 ± 11.4	71.2 ± 8.4	0.713	[−6.28; 6.89]	0.533 (0.733–0.332)
Valine	252.6 ± 27.5	252.8 ± 29.3	252.6 ± 37.6	0.698	[−19.24; 19.63]	0.465 (0.632–0.299)

Averages are shown with standard deviation, p value, VIP score and AUC for each metabolite.

AUC: area under the curve; CI: confidence interval; OSA: obstructive sleep apnoea.

### Performance of NoSAS score with and without metabolite profile in OSA screening

The receiver operating characteristic (ROC) curve and logistic regression analyses were used to discover the most qualified metabolic candidates among the significant metabolites described above. The individual area under the curve (AUC) values for each of the metabolites and lipids are presented in Tables 2, 4 and Supplemental Table 1. The best metabolites and lipids were selected based on the Pearson’s correlation (supplemental file) to build the final model to predict OSA. The correlation between untargeted and targeted metabolites that were analysed was applied to confirm the results avoiding false findings. There was a good correlation to glutamic acid in both techniques (R² = 0.843), while glutamine had a poor correlation (R² = 0.110). Thus, glutamine was excluded to perform logistic regression to obtain the final model for predicting OSA.

**Figure 4:**
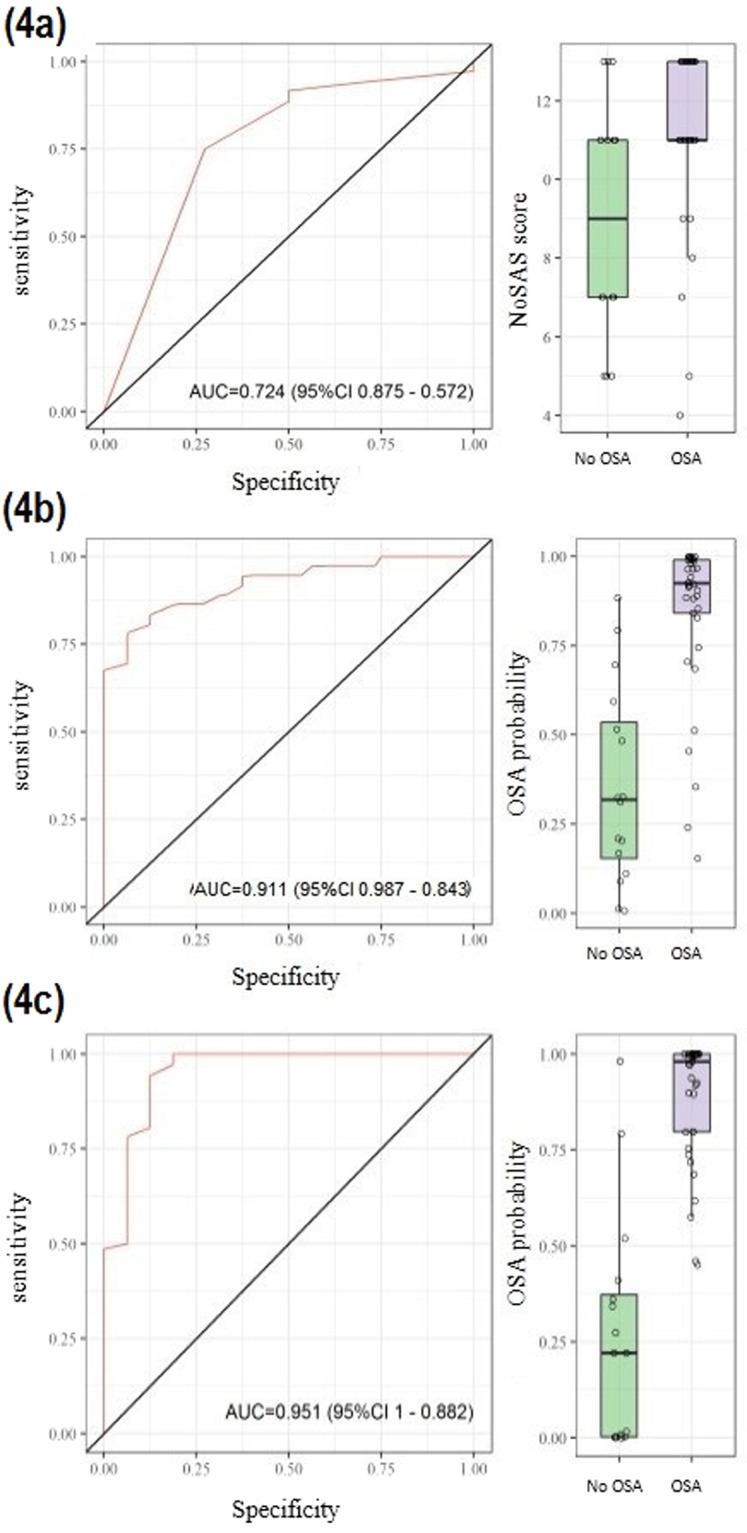
Receiver operating characteristic curve (ROC curve) with, respectively, AUC for (**a**) NoSAS score parameters, (**b**) metabolites statistically significant to screening OSA group and (**c**) lipids statistically significant to screening of OSA.

After collecting sleep questionnaires routinely used in screening OSA patients, we found the NoSAS score had an AUC = 0.724, p = 0.003 while the Berlin questionnaire had an AUC = 0.6565, p = 0.077. Therefore, the NoSAS score (Fig. 4a) was selected based on the better performance in screening OSA in this study. Interestingly, when coupling NoSAS score values to the three metabolites (glutamic acid, deoxy sugar and arachidonic acid) by logistic regression, we found an AUC of 0.911 (Fig. 4b) to propose an OSA biomarkers panel. In the lipidomic analysis, coupling the NoSAS score to the three selected lipids (PE 35:1, SM d18:1/12:0 and LPC 27:1) by logistic regression resulted in an AUC of 0.951 (Fig. 4c) to propose an OSA biomarkers panel.

## Discussion

The present investigation compared both metabolomic and lipidomic profiles in OSA patients and an appropriate control group matched for age, BMI, and body composition. Importantly, these patients had no previous CVD and were using no medications. We found that three metabolites involved in glucose and inflammatory pathways (glutamic acid, deoxy sugar and arachidonic acid) and three lipids (PE 35:1, SM d18:1/12:0 and LPC 27:1) had the best performance in separating the studied groups. Coupling the selected metabolites or lipids to the NoSAS score significantly improves the accuracy of detecting significant OSA. Taken together, our results identified potential early biomarkers of OSA, independent of traditional risk factors such as age and fat composition; these results suggest that these markers may add value in detecting OSA beyond traditional sleep questionnaires.

It is well-known that chronic intermittent hypoxia occurs during OSA^22^, and some molecular adaptations occur to adapt to this intermittent low-oxygen condition^23^. The most important transcriptional factors involved in all cellular response to hypoxia are the ubiquitous hypoxia-inducible factors (including HIF-1α)^24,25^. HIF-1α regulates the transcription of hundreds of genes to maintain a balance between O_2_ supply and demand in cells. When activated, HIF-1α activates a compendium of proteins involved in multiple pathways^26^, including metabolic responses such as the activation of glycolytic enzymes^25,27^. In our study, deoxy sugar was increased in OSA patients compared to the control group. Deoxy sugar is a monosaccharide glucose analogue produced by the action of glucose oxidase^28,29^. Previous studies have shown that OSA is associated with an imbalance between oxidant production and antioxidant activity^30^. This overabundance of oxidants may be associated with the multifactorial aetiology of metabolic disturbances, including insulin resistance^31^. Indeed, OSA is associated with insulin resistance and impaired glucose control^12^. Additional mechanisms and clinical implications concerning higher levels of deoxy sugar in OSA are unclear, but our findings suggest the biological plausibility of this association.

Growing evidence suggests that OSA promotes an inflammatory state that may partially explain the increased cardiovascular risk observed in these patients^32^. In our study, we detected several molecules involved with inflammatory pathways. In low oxygen availability, such as in myocardial ischaemia, energy deficiencies and membrane failures are indicated by intracellular and extracellular changes in [Na^+^] and [K^+^], as well as by a large influx of calcium^33^. Although high cytosolic calcium concentrations may disrupt various intracellular functions, the activation of phospholipase A1 (PLA1), A2 (PLA2) and C (PLC) is considered most damaging under hypoxic conditions^32,33^. A study developed in Chicago^34^ was able to correlate PLA2 activation in paediatric OSA and obesity with the presence of endothelial dysfunction. Supporting causality, OSA treatment promoted PLA2 inhibition^35^. PLA2 is an important enzyme required for repairing and remodelling cell membranes. It is also involved in the generation of lipid signalling molecules by hydrolysis of the *sn*-2 ester-bound-glycerophospholipids to free long chain fatty acids and 2-lysophospholipids^33,36^. Our study is consistent with these data since PC were down-regulated and LPC and arachidonic acid were up-regulated in patients with OSA. Free arachidonic acid synthesised by PLA2 may serve as a substrate for cyclooxygenase enzymes (COX-1 and COX-2) in the generation of prostaglandin E2^37^, which is believed to be critically involved in the mechanisms of regulation of vascular resistance, myocardial ischaemia, myocarditis and other CVD^32–36^. Other studies showed that arachidonic acid produced slowly developing inhibition of glutamate uptake^38^, a principal excitatory neurotransmitter in the brain^39^, and even though this study was related to the mechanisms in glial cells, we observed decreasing levels of glutamate in the biological fluid of patients with OSA.

**Figure 5:**
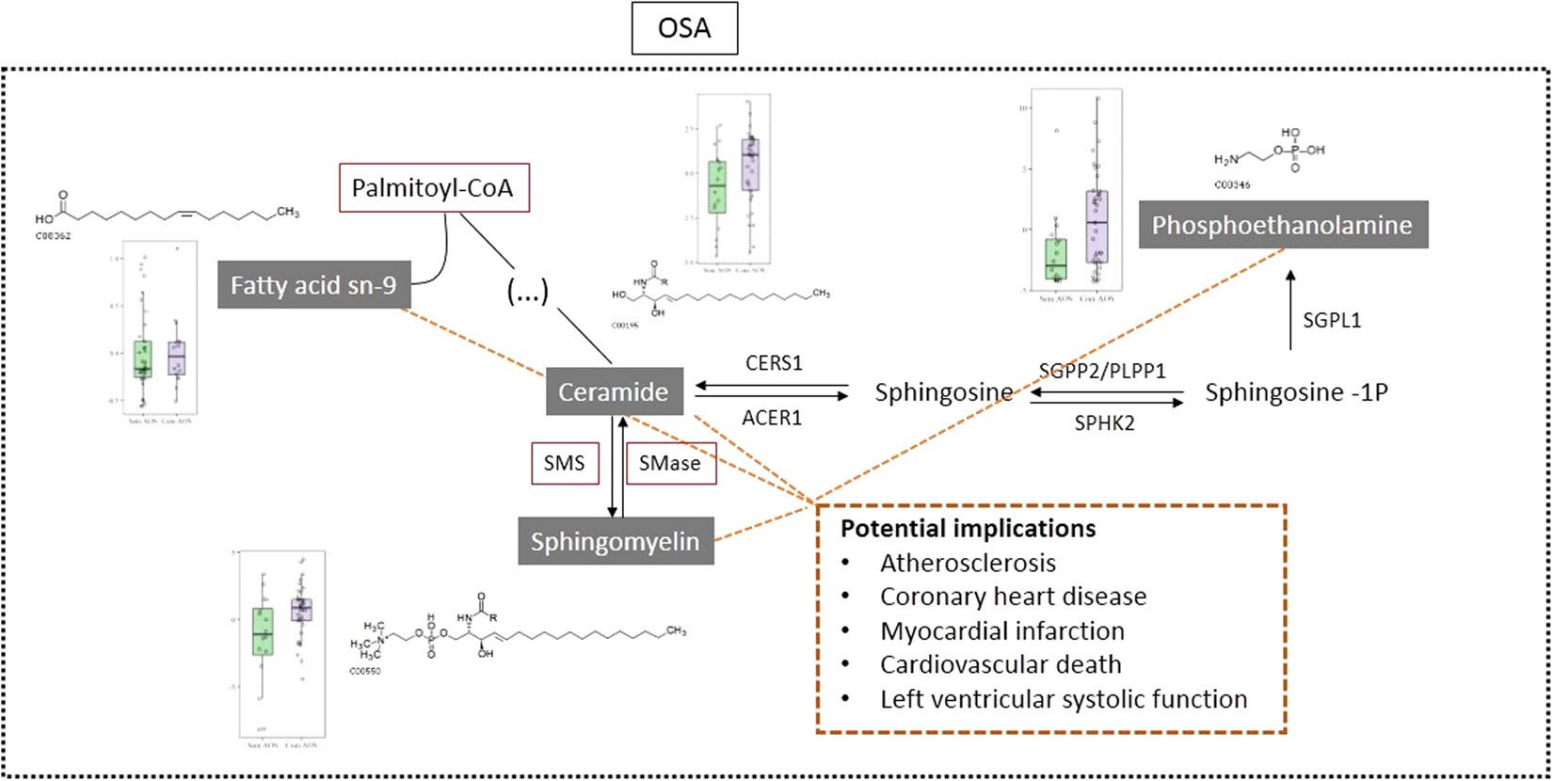
Adapted sphingolipid metabolism pathway with associated cardiovascular risk. Molecules statistically significant in metabolomic and lipidomic experiments are highlighted with probable enzymes activated during hypoxia.

Another target molecule observed in this study was sphingomyelin (SM d18:1/12:0). It is known that atherosclerotic lesions contain a high concentration of sphingolipids, with a strong association with inflammatory response, but the origin of these molecules is not clear^40^. They are also involved in cell apoptosis by proinflammatory cytokines such as TNF-alpha and interleukin-1^41^. One possible pathway for the metabolism of sphingolipids is shown on Fig. 5. Depending on the severity of hypoxia, studies show increased levels of ceramides and sphingomyelin by sphingomyelin phosphodiesterase (SMase) activation, as well as increased levels of sphingosine-1-phosphate (S1P) and ethanolamine^41^. Our study corroborates this information, since we observed elevated levels of ceramides and sphingomyelin, as well as increased levels of ethanolamine in patients with OSA. OSA may activate the palmitoyl CoA enzyme, which is responsible for synthesis of fatty acid unsaturated in 9 position^42^ (p.e. 9-hexadecenoic acid). Sequentially, an increase in ceramides and phosphatidylethanolamines levels were observed, as well as SMase enzyme activation that increased sphingomyelin levels. All of these molecules are elevated in OSA patients independently of body fat composition, suggesting them as attractive biomarkers candidates.

As previously discussed, evidence from metabolomics and lipidomics profiles for OSA is scanty. Previous studies screened potential OSA biomarkers, but the lack of matched controls and/or careful control of medications or habits (such as smoking) prevent appropriate interpretation of the potential role of OSA^18–21^. Moreover, since all previous studies used untargeted metabolomics platforms, there are significant differences compared to our findings. For instance, Ferrarini *et al*.^19^ applied LC-QTOF-MS using plasma samples and found 14 significant metabolites between glycerophospholipids, porphyrins, fatty acid, eicosanoids, amino acids and peptides in OSA patients; unfortunately, that study did not control for previous diseases, gender and smoking status. In another investigation, Xu *et al*.^18^ reported 31 significant metabolites from phospholipid biosynthesis, carbohydrate metabolism, TCA, glutamate metabolism, nucleic acid metabolism, indoles and derivatives, and spermine and tyrosine metabolism with a sensitivity and specificity model of no more than 85% and 80%, respectively, to screen OSA patients in comparison with other sleep disorders. However, these researchers used urine samples for untargeted metabolomics analysis. Recently, miRNAs measured from exosomes^21^ and proteomics^20^ have gained interest as potential biomarkers for OSA. Despite the analytical differences, all of these recent studies highlight the growing interest and challenges in pursuing reliable OSA biomarkers^43^.

Our study had strengths and limitations that should be acknowledged. Standard polysomnography, which is considered the gold standard method for diagnosing OSA, was used. A matched control group was carefully selected to avoid the obvious impact of conflicting factors on the metabolic analysis. A targeted LC-MS/MS approach was used to confirm the findings from the untargeted analysis of a select group of metabolites (amino acids). The following limitations should be addressed. First, this exploratory study comprised a relatively small sample size of young male subjects under no medication. These facts may limit the generalizability of our findings. While it is interesting to explore potential pathways related to OSA, these results may not be true for women or patients with significant comorbidities. Second, this is a cross-sectional study. No cause-and-effect relationship may be claimed in this investigation. An ongoing randomized study addressing OSA treatment may help to clarify the potential role of OSA in the metabolomic/lipidomic profile. Finally, we share the opinion that biomarkers are most useful for clarifying pathways and predicting cardiovascular risk, rather than for use as a complementary tool for OSA diagnosis. We are not proposing to use biomarkers to OSA diagnosis. However, there are considerable efforts to improve OSA screening and increase OSA diagnosis. All available sleep questionnaires do not have a great performance for screening OSA, as previously shown^44^ and confirmed by our study. Moreover, the PSG exam has long wait-lists and may causes some discomfort to patients. In this sense, it is necessary customize the selection of patients who merit objective sleep studies. However, it is not certain whether biomarkers are ideal candidates. This possibility would require significant improvement and validation using the analytical platform of choice, which, after optimization, could have low cost and absorb high-volume routines.

In conclusion, we identified plasma metabolites and lipids related to inflammatory and glycolytic metabolism in patients with OSA. Since these pathways are related to cardiovascular disease^32–37^ and these biomarkers may not be explained in our study as potential conflicting factors, such as age and fat composition, our results identified potential OSA biomarker candidates. Additional efforts to validate the selected metabolites in a population-based study may be helpful in the future to elucidate the potential role of biomarkers in OSA.

## Material and Methods

### Study population

This study was approved by the Ethics Committee at the University of São Paulo Medical School. Written informed consent was obtained from each participant. All methods were performed in accordance with the relevant guidelines and regulations. All participants were adult male non-smokers with no alcohol problems, no medications and no medical history of diabetes. Initially, we applied the Berlin questionnaire (see below) to determine patients with low and high risk for OSA. For each participant selected, we matched another participant for age (±5 years) and BMI (±3 kg/m²). It is important to mention, however, that the previous use of the Berlin questionnaire did not generate a potential bias because participants with low risk who presented OSA in the PSG crossed to the OSA group and vice versa (Fig. 1).

Participants who were diagnosed with central or mixed apnoea, as well as carriers of any pulmonary disease, CVD or chronic kidney disease, were excluded. Anthropometric parameters including height, weight, neck, waist and hip circumference were measured. BMI was calculated as weight/height^2^ (kg/m^2^). Blood pressure was measured in a quiet room three times at 5 min intervals, and the mean was calculated. Bioimpedance analysis was also performed for all included participants.

### Berlin questionnaire

The Berlin questionnaire^45^ is a 10-item questionnaire comprising questions from 3 categories (snoring, sleepiness and comorbidities). High risk for OSA is defined by 2 or more positive categories.

### NoSAS score

The NoSAS score includes variables such as neck circumference, obesity, snoring, age and gender. The score ranges from 0 to 17 points. We attributed 4 points for a neck circumference of >40 cm, 3 points for a body mass index (BMI) of 25 to <30 kg/m^2^, 5 points for a BMI of ≥30 kg/m^2^, 2 points for snoring, 4 points for being older than 55 years of age, and 2 points for male gender. A score ≥8 points defines a high probability of OSA^44^.

### Polysomnography evaluation

Participants were previously selected using the Berlin questionnaire, after which they underwent a full-night polysomnography (PSG) exam at the Sleep Laboratory (InCor). This sleep examination utilizes electroencephalography, electrooculography in both eyes, sub-mental electromyography, nasal airflow, snoring sounds, electrocardiography, thoracic/abdominal movements, pulse oxygen saturation and body position to measure various parameters. The PSG indices included were the apnoea-hypopnoea index (AHI) and oxygen desaturation index. Apnoea was defined as the decrease in airflow of at least 90% for ≥10 s, while hypopnea was defined as a drop in airflow of >30% for ≥10 s with oxygen desaturation ≥3%. The AHI was calculated using the number of respiratory events per hour of sleep according to the American Academy of Sleep Medicine criteria^46^. OSA was defined by AHI ≥15 events/h due to the lack of cardiovascular consequences of mild OSA^47^. All PSG indices were manually checked by the same technician to avoid variability. After the PSG, subjects were divided into two groups: 1) patients without OSA: AHI < 15 (n = 16); and 2) patients with OSA: AHI ≥ 15 (n = 37).

### Sample collection

Blood was obtained from all participants in the same place and at the same time of day (8:00 AM), after fasting for 12–14 hours; urine and saliva were collected by each participant at home. All samples were obtained using materials from the same manufacturer to avoid experimental artefacts. Plasma was collected by EDTA anticoagulant tubes for metabolomic and lipidomic analysis, serum was collected in gel tubes for biochemical analysis, and saliva was collected by Salivette, at three different times of day (between 7:00 AM and 9:00 AM, between 4:00 PM and 5:00 PM and between 11:00 PM and 12:00 AM), to measure cortisol alterations. Urine was collected over a period of 24 hours to measure catecholamine.

### Untargeted metabolomic analysis

GC-MS analysis was performed on an Agilent 7890B gas chromatograph (Agilent Technologies) coupled to an Agilent 5977 A MSD (Agilent Technologies). A 29-m long DB-5 MS column (0.25 mm × 0.25 μm) with a 10-m DuraGuard pre-column was used with other parameters as previously described^48^. An Agilent Mass Hunter WorkStation and Agilent Fiehn GC/MS Metabolomics RTL Library were employed for raw data processing and metabolite identification. The Agilent Fiehn GC/MS Metabolomics RTL Library has a list of metabolites that correlated the retention time with mass spectrum; the metabolites analysed are identified by an RTL (retention time lock) and mass spectrum using a chromatography method default by this library^48^. The chromatography method cannot be modified, in order to preserve the relationship with the library. The protocols to extract plasma samples used 400 μL of methanol (MeOH) and isopropanol (1:1 v/v), and 6 μL of internal standard (3 mg/mL d27-myristic acid, Sigma-Aldrich) added to 100 μL of plasma. The mixture was ultrasonicated for 5 min, mixed for 20 min at 4 °C and centrifuged at 15,800 × *g* at 4 °C for 10 min. The supernatant (350 μL) was transferred to an Eppendorf tube and dried for 16 h in a SpeedVac Savant SC210A. The pellet was derivatized in two steps. First, the carbonyl functional groups were protected by methoximation using 50 μL of a 40 mg/mL solution of methoxyamine hydrochloride in pyridine at 25 °C for 16 h. Next, the samples were derivatized using 100 μL of N-methyl-N-(trimethylsilyl)trifluoroacetamide with 1% trimethylchlorosilane (MSTFA + 1% TMCS, Sigma-Aldrich) at 25 °C for 60 min. After centrifugation at 15,800 × *g* at 4 °C for 10 min, 120 μL of the supernatant was transferred to a GC vial and injected. Quality control samples were extracted and derivatized in the same way as subject samples.

### Untargeted Lipidomic analysis

Lipids from plasma were extracted using a modified methyl tert-butyl ether (MTBE) method^49^. Plasma samples (250 µL) were combined with 1.25 mL of MeOH and 250 µL of water and the mixture was then stirred and centrifuged at 4000 × *g* for 10 min. The supernatant (1 mL) was transferred to a clean tube and extracted with MTBE (1.25 mL) by shaking the mixtures for 15 min at 850 rpm using Eppendorf Thermomixer vortex. After the addition of 1.5 mL of water, the upper phase was collected and dried in a SpeedVac Savant SPD131DDA. The dried extract was resuspended with 50 µL of isooctane. Ten microliters of the extract were fractionated by normal phase liquid chromatography using a UPLC I-Class (Waters Corporation) equipped with a Luna Silica (100 mm × 2 mm × 3 µm) column (Phenomenex). Lipid classes were separated using a binary gradient composed of dichloromethane (mobile phase A) and MeOH with 0.2% acetic acid (mobile phase B). At a constant flow rate of 0.9 mL/min, the proportion of B was ramped from 0 to 100% in 23 minutes. Nine fractions were collected using Gilson 215 Liquid Handler and dried in a SpeedVac for mass spectrometry analyses. MALDI-TOF-MS detection was performed on a Synapt G1 (Waters Corporation) in positive mode with an extraction voltage of 20 kV, a laser step rate of 20 kV and laser frequency and energy of 300 Hz. Lock mass solution (m/z 613,3411) was used to correct mass after acquisition. Mass range acquisition was 400–1700 m/z. Different matrix types (DHB 1 mol/L inMeOH:water (9:1 v/v) and 9-aminoacredine 10 mg/mL in IPA:ACN (6:4 v/v)) were used to detect features from each previously separated lipid class. Control samples used DHB 1 mol/L in MeOH:water (9:1 v/v) for MALDI detection. The raw data were processed and analysed using the R statistic package (MALDIquant and MALDIquantForeign). The algorithm for data processing starts with a raw unprocessed MALDI spectrum followed by smoothing, baseline correction, peak detection, merging and visualization of data. Lockmass correction was applied before R statistic package and was performed using commands previously developed by the Fleury Group. Finally, the results were exported as peak intensity list for statistical tests and mass identification. Each fraction was processed individually and feature identification was done using the LIPID MAPS library with an error of less than 50 ppm.

### Targeted metabolomics

Serum glucose, insulin, homeostasis model assessment of insulin resistance (HOMA-IR), thyroid-stimulating hormone (TSH), C-reactive protein (CRP) and lipid profile (TG, LDL, HDL, VLDL, total cholesterol and N-HDL) were measured by the Fleury Group using enzyme immunoassays from Roche Diagnostics GmbH. Serum adiponectin, interleukin-6 (IL-6), leptin and free fatty acids were measured using the enzyme-linked immunosorbent assay (ELISA), respectively: human adiponectin/Acrp30 ELISA, Sigma-Aldrich Co. LLC, human IL-6 sR ELISA, Sigma-Aldrich Co LLC, human leptin ELISA, Sigma-Aldrich Co. LLC, and free fatty acid quantitation kit, Sigma-Aldrich Co. LLC.

Salivary cortisol was quantified using a fully validated laboratory-developed test using UPLC-MS/MS^50^, and urinary catecholamine was quantified by HPLC and an electrochemical detector using a Chromsystems reagent kit (Catecholamines in Urine, ChromSystems^CE^ Instruments & Chemicals GmbH). Plasma amino acids were quantified by a fully validated laboratory-developed test using UPLC-MS/MS (Waters Corporation) using reversed-phase octadecylsilane (BEH C18 – Waters Technologies) with perfluoropentanoic acid as the ion-pairing agent^51^.

### Statistical analysis

The statistical analysis was performed using MetaboAnalyst 3.0 and the R package. For untargeted metabolomics and lipidomics, normalized data were mean-centred and divided by the square root of the standard deviation of each variable (Pareto Scaling). Principal component analysis (PCA) and supervised partial least-squares discriminant analysis (PLS-DA) were performed to visualize the metabolic and lipid differences between the groups. Significantly altered metabolites and lipids with a VIP score >2 in PLS-DA models and Student’s t-test (*p* < 0.05) were selected. A similar approach was used by Xu and colleagues^18^. For targeted metabolomics, Student´s t-test (p < 0.05) was applied to compare variables. Pearson’s correlation was performed between significant metabolites and OSA markers (AHI, minimum saturation and TTS < 90%) as well as between targeted and untargeted metabolomics in order to validate the results. The metabolites confirmed by LC-MS/MS approach (targeted metabolomics) and other metabolites and lipids which were statistically significant with non-zero correlation to OSA markers were submitted to logistic regression, concomitantly with NoSAS score values and stepwise regression using Akaike criteria^52^ to highlight a potential biomarker panel for OSA.

## Electronic supplementary material

Supplementary Information
