# Supplementary material for: Metabolomic and lipidomic profile in men with obstructive sleep apnoea: implications for diagnosis and biomarkers of cardiovascular risk
Source: Sci Rep. 2018 Jul 26;8:11270. doi: 10.1038/s41598-018-29727-6 (PMC6062521; doi:10.1038/s41598-018-29727-6)
Supplement: Supplementary file 1 — Supplementary Information [file 41598_2018_29727_MOESM1_ESM.docx]

**Supplemental data**

**Metabolomic and lipidomic profile in men with obstructive sleep apnoea: implications for diagnosis and biomarkers of cardiovascular risk**

Adriana Lebkuchen¹^,^², Valdemir M. Carvalho², Gabriela Venturini³, Jéssica S. Salgueiro², Lunara S. Freitas¹, Alessandra Dellavance^2^, Franco C. Martins^4^, Geraldo Lorenzi-Filho^4^, Karina H. M. Cardozo², Luciano F. Drager^1^

¹Unidade de Hipertensão, Instituto do Coração, Faculdade de Medicina, Universidade de São Paulo, São Paulo, SP

²Grupo Fleury, São Paulo, SP

³ Laboratório de Genética e Cardiologia Molecular, Instituto do Coração, Faculdade de Medicina, Universidade de São Paulo, São Paulo, SP

^4^Laboratório do sono, Instituto do Coração, Faculdade de Medicina, Universidade de São Paulo, São Paulo, SP

**Correspondence and requests for materials should be addressed to (email: drikalebe@hotmail.com) or (email: luciano.drager@incor.com.br). Hypertension Unit, Heart Institute (InCor), University of Sao Paulo Medical School, Avenida Dr Eneas de Carvalho Aguiar, 44, CEP 05403-900 Sao Paulo, Brazil. Tel: (5511) 26615084 / FAX: (5511) 26615948**

**Supplemental Table 1**

| **Metabolite** | **No OSA**  **(n = 16)** | **OSA**  **(n = 37)** | **p value** | **VIP score** | **CR dif. 95%** | **AUC (CR 95%)** |
| --- | --- | --- | --- | --- | --- | --- |
| (R*,R*)-2,3-Dihydroxybutanoic acid, tris(trimethylsilyl)- | 3.9 ± 2.2 | 3.6 ± 2.8 | 0.479 | 0.634 | [-1.45; 1.5] | 0.531 (0.715 - 0.347) |
| alpha-D-Galactopyranose, 2,3,4,6-tetrakis-O-(trimethylsilyl)-, bis(trimethylsilyl) phosphate | 4.7 ± 2.8 | 5.0 ± 3.3 | 0.930 | 0.149 | [-2.56; 0.97] | 0.579 (0.763 - 0.394) |
| .alpha.-Tocopherol, TMS derivative | 48 ± 24.4 | 49 ± 17.8 | 0.607 | 0.354 | [-5.73; 15.48] | 0.568 (0.768 - 0.367) |
| beta-D-Galactofuranose, 1,2,3,5,6-pentakis-O-(trimethylsilyl)- | 3.7 ± 1.5 | 3.3 ± 1.5 | 0.366 | 0.877 | [-0.65; 0.9] | 0.525 (0.726 - 0.323) |
| beta-Gentiobiose, octakis(trimethylsilyl) ether, methyloxime (isomer 1) | 0.2 ± 0.2 | 0.3 ± 0.3 | 0.171 | 1.213 | [-0.16; 0.18] | 0.549 (0.758 - 0.34) |
| beta-Tocopherol, TMS derivative | 1.0 ± 0.4 | 1.2 ± 0.6 | 1.000 | 1.235 | [-0.47; -0.02] | 0.692 (0.859 - 0.525) |
| delta-Tocopherol, TMS derivative | 0.2 ± 0.2 | 0.3 ± 0.2 | 0.198 | 0.777 | [-0.12; 0.11] | 0.504 (0.74 - 0.268) |
| gamma-Tocopherol, TMS derivative | 5.1 ± 1.1 | 6.1 ± 2.1 | 0.437 | 0.356 | [-1.85; -0.05] | 0.671 (0.819 - 0.522) |
| [1015] O-phosphocolamine [16.232] | 9.4 ± 2.5 | 8.7 ± 2.4 | 0.236 | 1.690 | [-0.84; 2.14] | 0.6 (0.774 - 0.425) |
| [1023] pyrophosphate [14.993] | 37.3 ± 9.0 | 36.8 ± 10.5 | 0.977 | 0.107 | [-5.44; 6.5] | 0.502 (0.674 - 0.329) |
| [10467] arachidic acid [22.367] | 1.5 ± 0.8 | 1.1 ± 0.9 | 0.246 | 0.344 | [-0.39; 0.63] | 0.574 (0.77 - 0.379) |
| [1101] L- sorbose 1 [17.187] | 16.7 ± 11.6 | 13.5 ± 8.0 | 0.244 | 1.106 | [-3.12; 10] | 0.593 (0.818 - 0.367) |
| [1110] succinic acid [10.509] | 10.4 ± 4.0 | 10.0 ± 3.9 | 0.268 | 1.140 | [-1.07; 3.14] | 0.607 (0.776 - 0.438) |
| [1175] uric acid 1 [19.331] | 203.9 ± 51.2 | 196.4 ± 46.1 | 0.871 | 0.183 | [-23.13; 38.06] | 0.539 (0.72 - 0.358) |
| [169019] D-threitol [12.954] | 8.5 ± 6.3 | 11.1 ± 4.1 | 0.304 | 0.111 | [-3.97; 3.3] | 0.599 (0.81 - 0.388) |
| [18950] D-mannose 1 [17.287] | 45.4 ± 9.6 | 45.0 ± 12.2 | 0.931 | 0.081 | [-5.95; 6.75] | 0.508 (0.679 - 0.338) |
| [219984] 1,5-anhydro-D-sorbitol [16.967] | 178.8 ± 65.9 | 180.4 ± 59.7 | 0.962 | 0.541 | [-41.08; 37.74] | 0.52 (0.694 - 0.346) |
| [236] L-asparagine 2 [14.984] | 4.7 ± 1.7 | 5.0 ± 1.9 | 0.508 | 0.463 | [-1.38; 0.74] | 0.562 (0.729 - 0.396) |
| [311] citric acid [16.615] | 65.5 ± 13.9 | 68.9 ± 19.1 | 0.625 | 0.674 | [-12.91; 6.06] | 0.515 (0.693 - 0.337) |
| [33032] L-glutamic acid 2 [14.398] | 25.8 ± 9.8 | 20.7 ± 9.2 | 0.331 | 0.303 | [-0.89; 10.96] | 0.659 (0.824 - 0.494) |
| [3893] lauric acid [14.789] | 3.8 ± 1.2 | 3.9 ± 2.8 | 0.285 | 0.793 | [-1.26; 0.96] | 0.574 (0.746 - 0.403) |
| [4114] xanthotoxin 2 [20.715] | 109.2 ± 32.3 | 117.4 ± 36.1 | 0.471 | 0.532 | [-28.65; 12.24] | 0.554 (0.721 - 0.387) |
| [439746] 6-deoxy-D-glucose 2 [15.751] | 2.6 ± 1.8 | 4.4 ± 2.5 | 0.021 | 2.637* | [-2.35; 0.28] | 0.667 (0.883 - 0.451) |
| [445638] palmitoleic acid [18.728] | 23 ± 10.1 | 24.6 ± 13.9 | 0.625 | 0.097 | [-8.44; 5.33] | 0.505 (0.679 - 0.331) |
| [5280450] linoleic acid [20.399] | 100.3 ± 42.8 | 96.4 ± 47.3 | 0.811 | 0.253 | [-23.15; 30.81] | 0.539 (0.705 - 0.372) |
| [5364509] methyl oleate [19.44] | 150 ± 75.6 | 138.0 ± 50.4 | 0.414 | 0.253 | [-30.9; 54.94] | 0.537 (0.72 - 0.354) |
| [5460407] threonic acid [13.652] | 21.8 ± 10.7 | 20.9 ± 16.8 | 0.529 | 0.721 | [-8.03; 7.58] | 0.557 (0.719 - 0.395) |
| [5780] D-sorbitol [17.898] | 2.9 ± 5.0 | 2.2 ± 2.0 | 0.429 | 0.433 | [-3.13; 6.67] | 0.496 (0.761 - 0.23) |
| [5810] trans-4-hydroxy-L-proline 1 [12.615] | 6.7 ± 2.7 | 7.5 ± 3.1 | 0.962 | 0.174 | [-2.73; 0.64] | 0.618 (0.792 - 0.444) |
| [5962] L-lysine 2 [17.643] | 103.8 ± 26.6 | 107.1 ± 19.4 | 0.680 | 0.041 | [-18.58; 11.96] | 0.483 (0.675 - 0.291) |
| [6057] tyrosine 1 [17.354] | 22.6 ± 7 | 26.8 ± 8.3 | 0.024 | 1.308 | [-6.88; -0.74] | 0.697 (0.853 - 0.542) |
| [6057] tyrosine 2 [17.871] | 94.6 ± 25.1 | 93.3 ± 18.5 | 0.392 | 1.235 | [-13.25; 15.69] | 0.524 (0.714 - 0.334) |
| [6137] L-methionine 2 [13.188] | 5.4 ± 0.9 | 6.0 ± 1.3 | 0.313 | 0.521 | [-1.19; 0.08] | 0.627 (0.794 - 0.46) |
| [614] L-proline 2 [10.341] | 34.3 ± 20.4 | 29.5 ± 17.5 | 0.338 | 0.972 | [-10.21; 12.28] | 0.522 (0.757 - 0.286) |
| [6161490] trans-13-octadecenoic acid [20.608] | 33.2 ± 24.6 | 30.9 ± 26.8 | 0.448 | 0.601 | [-13.06; 17.78] | 0.566 (0.737 - 0.395) |
| [6274] L-histidine 3 [17.658] | 23 ± 8.8 | 23.9 ± 6.8 | 0.260 | 1.072 | [-4.32; 4.89] | 0.481 (0.661 - 0.301) |
| [6287] L-valine 2 [9.151] | 7.7 ± 9.3 | 9.9 ± 9.2 | 0.264 | 0.641 | [-9.52; 12.69] | 0.447 (0.727 - 0.167) |
| [6288] L-threonine 1 [10.224] | 84.5 ± 43.9 | 79.9 ± 45 | 0.854 | 0.185 | [-16.28; 32.07] | 0.583 (0.766 - 0.4) |
| [6305] L-tryptophan 2 [20.466] | 64.9 ± 25.6 | 68.6 ± 28.0 | 0.598 | 0.672 | [-19.76; 12.33] | 0.552 (0.714 - 0.391) |
| [637517] elaidic acid [20.508] | 228.2 ± 56.7 | 213.7 ± 56.6 | 0.129 | 0.812 | [-20.16; 49.21] | 0.566 (0.738 - 0.394) |
| [643801] methyl palmitoleate [17.466] | 63.1 ± 45.2 | 56.3 ± 30.6 | 0.723 | 0.440 | [-18.93; 32.5] | 0.498 (0.68 - 0.316) |
| [64956] DL-3-aminoisobutyric acid 2 [12.457] | 12.1 ± 5.3 | 10.8 ± 3.9 | 0.197 | 0.336 | [-0.58; 4.5] | 0.655 (0.853 - 0.458) |
| [65098] norvaline 2 [9.468] | 60.8 ± 13.7 | 60.5 ± 14.1 | 0.962 | 0.154 | [-8.23; 8.69] | 0.502 (0.675 - 0.328) |
| [6665] 5alpha-Cholestanol [27.436] | 2.6 ± 1.4 | 2.4 ± 1.4 | 0.432 | 0.314 | [-0.32; 1.15] | 0.662 (0.848 - 0.476) |
| [67678] L-cystine 2 [20.862] | 7.3 ± 2.6 | 7.2 ± 2.9 | 0.371 | 0.521 | [-1.57; 1.7] | 0.52 (0.702 - 0.338) |
| [67678] L-cystine 3 [21.104] | 10.0 ± 3.6 | 10.0 ± 3.8 | 0.277 | 0.861 | [-2.22; 2.25] | 0.49 (0.666 - 0.314) |
| [738] L-glutamine 3 [16.092] | 65.9 ± 31.1 | 62.7 ± 25.0 | 0.008* | 2.806* | [-11.82; 22.72] | 0.556 (0.742 - 0.37) |
| [7405] L-pyroglutamic acid [13.218] | 76.7 ± 28.8 | 60.7 ± 29.1 | 0.367 | 1.080 | [-9.69; 24.66] | 0.554 (0.737 - 0.372) |
| [750] glycine [10.456] | 59.5 ± 19.5 | 55.0 ± 15.3 | 0.520 | 0.758 | [-6.8; 15.83] | 0.443 (0.619 - 0.266) |
| [754] glycerol 1-phosphate [16.056] | 26.2 ± 18.7 | 31.4 ± 32.0 | 0.414 | 0.565 | [-19.4; 8.91] | 0.534 (0.703 - 0.365) |
| [827] ribitol [15.66] | 5.4 ± 2.4 | 5.5 ± 2.9 | 0.708 | 0.010 | [-1.64; 1.5] | 0.542 (0.725 - 0.359) |
| [8343] dioctyl phthalate [23.163] | 32.5 ± 15.3 | 29.6 ± 12.0 | 0.508 | 0.501 | [-5.96; 11.84] | 0.556 (0.743 - 0.369) |
| [8897] iminodiacetic acid 1 [12.487] | 40.6 ± 16.3 | 42.5 ± 12.3 | 0.962 | 0.548 | [-11.34; 7.47] | 0.586 (0.778 - 0.395) |
| [892] myo-inositol [19.354] | 55.9 ± 16.3 | 54.8 ± 21.2 | 0.124 | 1.285 | [-9.85; 11.87] | 0.554 (0.725 - 0.383) |
| [92824] D-malic acid [12.794] | 3.7 ± 1.6 | 3.5 ± 2.3 | 0.425 | 1.147 | [-0.97; 1.25] | 0.562 (0.722 - 0.403) |
| [9316] methyl linolenate [19.432] | 73.6 ± 45.9 | 64.6 ± 34.4 | 0.184 | 0.977 | [-20.31; 32.62] | 0.529 (0.717 - 0.34) |
| [938] nicotinic acid [10.27] | 2.5 ± 1.3 | 2.9 ± 1.2 | 0.170 | 1.198 | [-0.64; 0.48] | 0.528 (0.72 - 0.336) |
| [9750] citrulline 2 [16.691] | 1.4 ± 0.8 | 1.3 ± 0.8 | 0.158 | 1.222 | [-0.53; 0.48] | 0.513 (0.715 - 0.311) |
| [985] palmitic acid [18.846] | 134.3 ± 34.1 | 143.3 ± 30.8 | 0.931 | 1.238 | [-29.39; 11.41] | 0.596 (0.768 - 0.424) |
| [RTL] Myristic acid d27 [16.727] | 289.5 ± 44.1 | 296 ± 41.8 | 0.886 | 0.812 | [-33.16; 20.16] | 0.551 (0.728 - 0.373) |
| {4-Methoxy-2-[(trimethylsilyl)oxy]phenyl}{2-[(trimethylsilyl)oxy]phenyl}methanone | 1.6 ± 1.0 | 1.5 ± 1.0 | 0.913 | 0.182 | [-0.73; 0.43] | 0.63 (0.832 - 0.429) |
| 1-(Trifluoroacetyl)-L-proline, TMS derivative | 0.5 ± 0.2 | 0.5 ± 0.2 | 0.846 | 0.234 | [-0.02; 0.18] | 0.64 (0.814 - 0.466) |
| 1,2-Dipalmitin, TMS derivative | 6.4 ± 4.9 | 6.0 ± 4.1 | 0.886 | 0.180 | [-2.54; 3.27] | 0.505 (0.693 - 0.317) |
| 1,3-Dipalmitin, TMS derivative | 5.9 ± 2.8 | 6.2 ± 3.5 | 0.425 | 0.664 | [-2.11; 1.6] | 0.485 (0.672 - 0.297) |
| 1,3-Propanediol, 2TMS derivative | 47.3 ± 16.4 | 42.2 ± 16.8 | 0.392 | 0.384 | [-4.97; 15.25] | 0.618 (0.791 - 0.445) |
| 1,5-Diformyl-2,6-dimethoxy-anthracene | 2.4 ± 1.6 | 2.6 ± 1.4 | 0.535 | 0.624 | [-0.66; 1.15] | 0.584 (0.816 - 0.353) |
| 10-Heptadecenoic acid, (Z)-, TMS derivative | 0.5 ± 0.4 | 0.8 ± 0.6 | 0.106 | 1.327 | [-0.56; 0.12] | 0.628 (0.859 - 0.397) |
| 13-Docosenoic acid, methyl ester, (Z)- | 79.0 ± 45.7 | 71.3 ± 35.6 | 0.124 | 1.561 | [-18.78; 34.26] | 0.559 (0.736 - 0.382) |
| 1-Chloromethyl-1-hexadecyloxy-1-silacyclohexane | 50.6 ± 78.6 | 70.6 ± 74.4 | 0.307 | 1.325 | [-75.03; 67.01] | 0.421 (0.705 - 0.136) |
| 1-Heptanol, TMS derivative | 6.0 ± 2.4 | 6.1 ± 2.1 | 0.529 | 0.173 | [-1.59; 1.02] | 0.582 (0.789 - 0.375) |
| 1H-Indole-2,3-dione, 1-(tert-butyldimethylsilyl)-7-propyl-, 3-(O-methyloxime) | 2.6 ± 2.2 | 2.6 ± 1.8 | 1.000 | 0.195 | [-1.26; 1.8] | 0.539 (0.745 - 0.333) |
| 1H-Pyrazole, 4-nitro- | 9.2 ± 2.1 | 8.7 ± 2.6 | 0.708 | 0.542 | [-1; 1.7] | 0.557 (0.73 - 0.385) |
| 1-Monomyristin, 2TMS derivative | 58.1 ± 11.2 | 56.1 ± 9.9 | 0.285 | 1.276 | [-4.7; 8.65] | 0.53 (0.711 - 0.35) |
| 2-(Methylamino)ethanol, tert-butyldimethylsilyl ether | 7.1 ± 2.8 | 4.4 ± 2.9 | 0.069 | 1.915 | [-0.16; 3.28] | 0.654 (0.832 - 0.476) |
| 2,3-Dihydroxypropyl icosanoate, 2TMS derivative | 8.4 ± 1.5 | 8.6 ± 1.5 | 0.840 | 0.072 | [-1.08; 0.79] | 0.529 (0.71 - 0.347) |
| 2,5-Di-tert-butyl-4-((trimethylsilyl)oxy)phenol | 3.0 ± 0.9 | 2.9 ± 0.9 | 0.900 | 0.165 | [-0.46; 0.41] | 0.515 (0.699 - 0.331) |
| 2,6-Bis(tert-butyl)phenol, TMS derivative | 5.7 ± 3.4 | 5.0 ± 2.6 | 0.811 | 0.151 | [-1.33; 2.56] | 0.537 (0.718 - 0.356) |
| 2,6-Diphenyl-1,7-dihydrodipyrrolo[2,3-b:3',2'-e]pyridine | 9.2 ± 3.2 | 10.2 ± 3.4 | 0.021* | 3.046* | [-3.64; 0.15] | 0.693 (0.872 - 0.514) |
| 2-Ethyl-3-hydroxypropionic acid, di-TMS | 1.7 ± 0.8 | 1.6 ± 0.9 | 0.502 | 0.433 | [-0.37; 0.44] | 0.544 (0.743 - 0.344) |
| 2H,8H-Benzo[1,2-b:3,4-b']dipyran-6-propanoic acid, 5-methoxy-2,2,8,8-tetramethyl- | 1.0 ± 0.5 | 0.9 ± 0.6 | 0.272 | 1.212 | [-0.48; 0.1] | 0.672 (0.886 - 0.457) |
| 2-Ketoisocaproic acid mo-tms | 19.3 ± 5.9 | 18.4 ± 5.1 | 0.086 | 1.470 | [-2.6; 4.39] | 0.47 (0.645 - 0.294) |
| 2-Monostearin, 2TMS derivative | 99.5 ± 25.9 | 95.8 ± 27.2 | 1.000 | 0.286 | [-12.3; 19.81] | 0.554 (0.725 - 0.383) |
| 2-Palmitoylglycerol, 2TMS derivative | 124.1 ± 23.4 | 126.6 ± 32.4 | 0.625 | 0.354 | [-18.6; 13.43] | 0.519 (0.686 - 0.351) |
| 2-Propanol, 1,3-dibromo- | 5.1 ± 3.3 | 4.9 ± 3.2 | 0.897 | 0.488 | [-0.61; 1.88] | 0.538 (0.738 - 0.337) |
| 3-.alpha.-Mannobiose, octakis(trimethylsilyl) ether (isomer 2) | 35.9 ± 26.5 | 38.3 ± 23.6 | 0.620 | 0.441 | [-12.27; 18.03] | 0.539 (0.743 - 0.335) |
| 3-[3-(4-Hydroxyphenyl)-3-oxoprop-1-en-1-yl]-6-methylchromen-4-one | 2.1 ± 1.3 | 2.0 ± 1.3 | 0.594 | 0.489 | [-0.58; 0.76] | 0.481 (0.701 - 0.261) |
| 3-Indoleacetic acid, 2TMS derivative | 2.7 ± 1.6 | 3.2 ± 1.8 | 0.454 | 0.633 | [-1.38; 0.5] | 0.581 (0.779 - 0.382) |
| 3-Pyridinol, TMS derivative | 2.5 ± 2.7 | 5.2 ± 5.1 | 0.063 | 1.283 | [-4.68; 0.78] | 0.569 (0.765 - 0.373) |
| 4-Bromo-1-butanol, TBDMS derivative | 32.3 ± 11.7 | 28.4 ± 11.9 | 0.102 | 1.687 | [-3.98; 10.32] | 0.566 (0.745 - 0.387) |
| 5,5'-Biphthalide | 2.2 ± 1.9 | 1.6 ± 2.1 | 0.019* | 2.229* | [-1.41; 1.41] | 0.528 (0.739 - 0.317) |
| 6-Hydroxy-.alpha.-methylnaphthaleneacetic acid, di-TMS | 125.7 ± 23.8 | 135.4 ± 26.0 | 0.013 | 1.230 | [-24.59; 5.26] | 0.62 (0.78 - 0.46) |
| 9-Hexadecenoic acid, (Z)-, TBDMS derivative | 73.3 ± 13.2 | 77.4 ± 13.7 | 0.011* | 2.837* | [-12.34; 3.98] | 0.605 (0.776 - 0.434) |
| 9H-Purin-6-ol, 2TMS derivative | 1.1 ± 0.5 | 0.7 ± 0.5 | 0.058 | 0.867 | [-0.12; 0.42] | 0.649 (0.838 - 0.46) |
| Acetamide, TMS derivative | 7.1 ± 1.7 | 6.0 ± 3.2 | 0.099 | 2.474 | [-1.57; 0.6] | 0.555 (0.736 - 0.373) |
| Aminomalonic acid, tris(trimethylsilyl)- | 8.4 ± 6.9 | 9.7 ± 5.3 | 0.432 | 1.061 | [-4.44; 4.31] | 0.535 (0.763 - 0.308) |
| Anthraquinone, 1-(o-chlorophenyl)- | 9.2 ± 2.1 | 9.5 ± 3.2 | 0.977 | 0.046 | [-1.8; 1.15] | 0.507 (0.668 - 0.346) |
| Arachidonic acid, TMS derivative | 7.3 ± 2.5 | 10.1 ± 11.9 | 0.036* | 2.381* | [-6.87; 1.39] | 0.596 (0.758 - 0.435) |
| Cholest-7-en-3-ol, (3.beta.,5.alpha.)-, TMS derivative | 2.6 ± 0.8 | 3.4 ± 1.5 | 0.115 | 1.362 | [-1.43; -0.13] | 0.647 (0.801 - 0.493) |
| Cholestanol, 5,6-eposy-, acetate(ester) | 3.9 ± 1.3 | 3.7 ± 1.8 | 0.211 | 1.106 | [-1.18; 0.52] | 0.55 (0.728 - 0.373) |
| Cholesterol, TMS derivative | 161.5 ± 72.0 | 163.9 ± 85.3 | 0.776 | 0.136 | [-49.19; 24.33] | 0.532 (0.73 - 0.334) |
| Cyclodecasiloxane, eicosamethyl- | 149.5 ± 113.7 | 123.5 ± 99.9 | 0.154 | 1.529 | [-21.94; 114.48] | 0.664 (0.826 - 0.502) |
| Cyclohexene, 3-butyl-3,5,5-trimethyl-1-(trimethylsilyloxy)- | 2.7 ± 0.9 | 2.8 ± 1.0 | 0.309 | 0.940 | [-0.52; 0.31] | 0.54 (0.728 - 0.353) |
| Cyclononasiloxane, octadecamethyl- | 19.6 ± 20.5 | 14.9 ± 18.3 | 0.900 | 0.037 | [-6.08; 20.01] | 0.615 (0.796 - 0.435) |
| Cyclooctasiloxane, hexadecamethyl- | 17.7 ± 17.2 | 15.2 ± 16.3 | 0.078 | 1.598 | [-5.31; 17.27] | 0.652 (0.82 - 0.484) |
| Diethanolamine, 2TMS derivative | 5.4 ± 1.3 | 4.7 ± 1.5 | 0.162 | 1.255 | [-0.23; 1.42] | 0.618 (0.787 - 0.449) |
| DL-Ornithine, 3TMS derivative | 18.6 ± 7.1 | 17.8 ± 6.5 | 0.437 | 0.648 | [-3.49; 4.98] | 0.542 (0.713 - 0.372) |
| DL-Phenylalanine, TMS derivative | 26.6 ± 5.8 | 26.4 ± 4.5 | 0.855 | 0.297 | [-3.19; 3.55] | 0.495 (0.674 - 0.316) |
| Doconexent, TMS derivative | 1.6 ± 1.0 | 2.2 ± 1.2 | 0.268 | 0.684 | [-0.97; 0.26] | 0.588 (0.803 - 0.373) |
| Edetic Acid, 4TMS derivative | 176.8 ± 67.0 | 183.4 ± 60.8 | 0.392 | 0.372 | [-46.63; 33.55] | 0.578 (0.754 - 0.402) |
| Ethanol, 2-(methylamino)-, N-trifluoroacetyl, O-(tert-butyldimethylsilyl) | 42.8 ± 28.3 | 41.6 ± 24.5 | 0.937 | 0.105 | [-13.66; 18.79] | 0.524 (0.749 - 0.299) |
| Ethanolamine, 3TMS derivative | 28.3 ± 16.9 | 30.8 ± 14.5 | 0.691 | 0.304 | [-10.35; 7.81] | 0.494 (0.686 - 0.301) |
| Glucose, 5TMS derivative | 26.3 ± 37.6 | 27.5 ± 33.8 | 0.438 | 0.994 | [-27.37; 35.46] | 0.559 (0.767 - 0.351) |
| Glyceric acid, 3TMS derivative | 10.6 ± 3.5 | 9.6 ± 4.8 | 0.737 | 0.112 | [-1.41; 3.37] | 0.593 (0.755 - 0.431) |
| Glycine, 2TMS derivative | 8.1 ± 4.4 | 8.9 ± 4.0 | 0.946 | 0.309 | [-2.26; 2.35] | 0.516 (0.711 - 0.321) |
| Heptadecanoic acid, glycerine-(1)-monoester, bis-O-trimethylsilyl- | 4.3 ± 0.9 | 4.3 ± 1.0 | 0.855 | 0.224 | [-0.64; 0.5] | 0.5 (0.678 - 0.322) |
| Heptadecanoic acid, TMS derivative | 2.3 ± 1.6 | 2.0 ± 1.1 | 0.219 | 1.684 | [-0.6; 1.24] | 0.522 (0.722 - 0.322) |
| Hexadecane | 4.9 ± 4.0 | 3.6 ± 3.2 | 0.184 | 1.046 | [-1.52; 3.48] | 0.583 (0.77 - 0.396) |
| Hydroxylamine, 3TMS derivative | 4.9 ± 2.0 | 4.8 ± 2.5 | 0.058 | 1.824 | [-1.42; 1.19] | 0.509 (0.678 - 0.339) |
| Lactic Acid, 2TMS derivative | 105.5 ± 69.7 | 104 ± 76.6 | 0.142 | 0.821 | [-42.28; 37.08] | 0.493 (0.681 - 0.306) |
| L-Alanine, 2TMS derivative | 26.4 ± 14.4 | 28 ± 12.5 | 0.120 | 0.798 | [-10.06; 6.97] | 0.579 (0.748 - 0.411) |
| L-Alanine, TMS derivative | 15.2 ± 5.0 | 14.3 ± 6.2 | 0.274 | 0.407 | [-3.22; 3.12] | 0.52 (0.699 - 0.341) |
| L-Aspartic acid, 2TMS derivative | 15.5 ± 11.1 | 10.8 ± 5.9 | 0.962 | 0.508 | [-1.45; 10.86] | 0.649 (0.83 - 0.467) |
| L-Isoleucine, 2TMS derivative | 31.4 ± 9.9 | 36.4 ± 16.2 | 0.826 | 0.436 | [-12.29; 2.38] | 0.578 (0.745 - 0.41) |
| L-Leucine, TMS derivative | 53.9 ± 15.0 | 44.8 ± 18.5 | 0.187 | 1.007 | [-2.76; 12.18] | 0.613 (0.788 - 0.438) |
| L-Methionine, TMS derivative | 11.0 ± 2.3 | 10.3 ± 2.3 | 0.351 | 1.209 | [-0.66; 2.17] | 0.59 (0.766 - 0.413) |
| L-Phenylalanine, 2TMS derivative | 29.9 ± 8.6 | 30.2 ± 5.6 | 0.871 | 0.041 | [-5.19; 4.59] | 0.508 (0.717 - 0.3) |
| L-Proline, 2TMS derivative | 45.9 ± 26.8 | 35.9 ± 13.6 | 0.886 | 0.340 | [-4.81; 24.78] | 0.598 (0.785 - 0.411) |
| L-Proline, TMS derivative | 75.2 ± 14.5 | 69.0 ± 16.3 | 0.520 | 0.011 | [-4.05; 13.69] | 0.616 (0.797 - 0.435) |
| L-Serine, 2TMS derivative | 40.4 ± 15.1 | 37.9 ± 17.3 | 0.207 | 0.816 | [-7.11; 12.12] | 0.578 (0.749 - 0.406) |
| L-Threonine, 3TMS derivative | 54.5 ± 18.7 | 57.2 ± 17.6 | 0.855 | 0.315 | [-13.94; 8.59] | 0.458 (0.641 - 0.274) |
| L-Valine, TMS derivative | 67.0 ± 24.6 | 53.4 ± 23.1 | 0.124 | 1.437 | [-1.24; 28.44] | 0.671 (0.832 - 0.509) |
| Methoxyamine, TMS derivative | 44.8 ± 27.1 | 47.3 ± 24.7 | 0.663 | 0.386 | [-16.48; 14.99] | 0.529 (0.718 - 0.341) |
| Methyl 2-[2-(4-chlorophenyl)-5-methyl-1H-imidazol-1-yl]dithiobenzoate | 4.9 ± 7.3 | 3.1 ± 3.5 | 0.375 | 0.727 | [-3.14; 6.05] | 0.495 (0.686 - 0.303) |
| Monolaurin, 2TMS derivative | 5.9 ± 1.0 | 6.2 ± 1.0 | 0.313 | 0.986 | [-0.86; 0.31] | 0.596 (0.762 - 0.43) |
| Myo-Inositol, 1,3,4,5,6-pentakis-O-(trimethylsilyl)-, bis(trimethylsilyl) phosphate | 2.3 ± 1.1 | 2.6 ± 1.4 | 0.688 | 0.197 | [-0.82; 0.23] | 0.589 (0.773 - 0.405) |
| N-(2-Acetamido)iminodiacetic acid, 3TMS derivative | 13.8 ± 7.2 | 16 ± 6.6 | 0.946 | 0.307 | [-5.61; 2.64] | 0.576 (0.763 - 0.388) |
| Nonanoic acid, TMS derivative | 3.8 ± 2.8 | 3.4 ± 1.7 | 0.389 | 0.320 | [-0.91; 2.41] | 0.54 (0.757 - 0.323) |
| Oxalic acid, 2TMS derivative | 180.5 ± 34.3 | 183.3 ± 54.9 | 0.694 | 0.075 | [-27.88; 22.33] | 0.549 (0.718 - 0.38) |
| Pentadecanoic acid, TMS derivative | 2.5 ± 0.7 | 2.6 ± 0.9 | 0.766 | 0.239 | [-0.65; 0.29] | 0.468 (0.64 - 0.296) |
| Pentanedioic acid, 2-(methoxyimino)-, bis(trimethylsilyl) ester | 10.0 ± 4.9 | 9.1 ± 4.9 | 0.572 | 1.000 | [-2.09; 3.13] | 0.549 (0.754 - 0.345) |
| Pentasiloxane, dodecamethyl- | 4.7 ± 3.1 | 4.3 ± 2.1 | 0.930 | 0.111 | [-0.24; 3.04] | 0.669 (0.854 - 0.484) |
| Phenol, 2,4-bis(1,1-dimethylethyl)-, phosphite (3:1) | 4.2 ± 1 | 4.4 ± 1.0 | 0.680 | 0.344 | [-0.78; 0.45] | 0.473 (0.648 - 0.298) |
| Phosphoric acid, 2-[(trimethylsilyl)oxy]-1,3-propanediyl tetrakis(trimethylsilyl) ester | 36.0 ± 20.9 | 44.9 ± 18.9 | 0.085 | 1.825 | [-11.66; 11.42] | 0.504 (0.769 - 0.239) |
| Phosphoric acid, bis(trimethylsilyl)monomethyl ester | 4.4 ± 1.1 | 4.5 ± 0.8 | 0.694 | 0.113 | [-0.4; 0.59] | 0.553 (0.728 - 0.378) |
| Pseudo uridine penta-tms | 7.9 ± 1.8 | 7.4 ± 2.2 | 0.268 | 0.208 | [-0.62; 1.69] | 0.633 (0.799 - 0.468) |
| Pyrazine, 2,3,5-tris-(trimethylsilyloxy)- | 1.2 ± 0.8 | 1.1 ± 0.7 | 0.443 | 1.123 | [-0.43; 0.58] | 0.495 (0.706 - 0.285) |
| Serine, 3TMS derivative | 40.7 ± 10.3 | 42.1 ± 10.4 | 0.708 | 0.606 | [-7.65; 4.98] | 0.47 (0.647 - 0.292) |
| Silanamine, N,N'-methanetetraylbis[1,1,1-trimethyl- | 5.8 ± 2.5 | 5.2 ± 2.6 | 0.123 | 1.725 | [-0.94; 1.63] | 0.576 (0.765 - 0.387) |
| Silane, diethyldecyloxyhexadecyloxy- | 0.5 ± 0.2 | 0.5 ± 0.2 | 0.181 | 1.254 | [-0.1; 0.11] | 0.538 (0.724 - 0.353) |
| Silanol, trimethyl-, phosphate (3:1) | 167.1 ± 37.4 | 156.5 ± 45.5 | 0.840 | 0.096 | [-16.65; 29.98] | 0.557 (0.74 - 0.375) |
| Tetrasiloxane, decamethyl- | 1.4 ± 0.3 | 1.4 ± 0.4 | 0.757 | 0.233 | [-0.17; 0.13] | 0.502 (0.67 - 0.334) |
| Undecane, 2,10-dimethyl- | 2.6 ± 1.3 | 2.2 ± 1.3 | 0.073 | 1.781 | [-0.72; 0.81] | 0.492 (0.676 - 0.308) |

**Supplemental Table 2**

|  | AHI | Minimum O_2_ saturation | TST O_2_ sat <90% |
| --- | --- | --- | --- |
| AHI | 1 |  |  |
| Minimum O_2_ saturation | -0.741* | 1 |  |
| TST O_2_ sat <90% | 0.782* | -0.861* | 1 |
| Neck circumference | 0.299* | -0.345* | 0.429* |
| Diastolic blood pressure | 0.2 | -0.061 | 0.106 |
| Glutamic acid | -0.224 | 0.102 | -0.068 |
| Glutamine | 0.365* | -0.392* | 0.403* |
| 2,6-Diphenyl-1,7-dihydrodipyrrolo[2,3-b:3',2'-e]pyridine | -0.153 | 0.209 | -0.209 |
| 9- hexadecenoic acid (Z) | 0.196 | -0.267 | 0.163 |
| 6-deoxy-D-glucose | 0.351* | -0.209 | 0.203 |
| 5,5'-biphthalide | -0.275* | 0.187 | -0.269 |
| Arachidonic acid | -0.31* | 0.397* | -0.204 |

*Correlation significantly different than zero.

*p<0.05; AHI: Apnoea-hypopnoea index; O2: oxygen; sat: saturation; TST: Total sleep time

**Supplemental table 3**

**Glycerolipid**

| Imput mass | Delta | Abbreviation | No OSA (n=16) | OSA (n=37) | p value |
| --- | --- | --- | --- | --- | --- |
| 479,3224 | .0488 | DG(24:0) | 1,82±0,75 | 1,48±0,34 | 0,1071 |
| 481,3147 | .0148 | DG(23:0) | 1,71±0,63 | 1,54±0,45 | 0,3878 |
| 493,2818 | .0477 | DG(24:1) | 1,64±0,77 | 1,41±0,39 | 0,2688 |
| 507,3100 | .0351 | DG(25:1) | 1,51±0,53 | 1,27±0,37 | 0,2388 |
| 527,3321 | .0391 | DG(28:4) | 1,63±0,71 | 1,39±0,37 | 0,2380 |
| 533,3109 | .0498 | DG(27:2) | 1,64±0,74 | 1,36±0,35 | 0,1635 |
| 535,3411 | .0354 | DG(27:1) | 1,70±0,78 | 1,42±0,39 | 0,1745 |
| 543,3137 | .0315 | DG(28:4) | 1,97±1,03 | 1,86±0,78 | 0,7388 |
| 545,3226 | .0382 | DG(28:3) | 1,71±0,79 | 1,39±0,39 | 0,1199 |
| 547,3400 | .0364 | DG(28:2) | 1,66±0,74 | 1,29±0,31 | 0,0871 |
| 561,4223 | .0271 | DG(30:1) | 1,96±0,74 | 1,55±0,31 | 0,0807 |
| 569,3863 | .0318 | DG(31:4) | 2,08±0,37 | 2,14±0,60 | 0,7946 |
| 571,3602 | .0163 | DG(30:4) | 1,36±0,39 | 1,22±0,16 | 0,1747 |
| 573,3503 | .0418 | DG(30:3) | 1,43±0,52 | 1,16±0,18 | 0,0968 |
| 575,3950 | .0128 | DG(30:2) | 1,83±0,78 | 1,47±0,32 | 0,0834 |
| 577,3790 | .0444 | DG(30:1) | 5,21±1,92 | 4,59±1,18 | 0,3086 |
| 579,5471 | .0125 | DG(O-34:2) | 26,26±4,85 | 27,21±4,72 | 0,6121 |
| 580,5500 | .0201 | DG(P-33:2) | 15,72±4,66 | 15,12±3,09 | 0,6561 |
| 581,5561 | .0058 | DG(P-34:0) | 10,98±2,95 | 10,66±1,93 | 0,7226 |
| 582,5631 | .0175 | DG(O-33:2) | 5,88±1,44 | 5,71±1,04 | 0,7025 |
| 585,3788 | .0132 | DG(31:4) | 2,02±0,35 | 2,03±0,29 | 0,9474 |
| 587,3831 | .0246 | DG(31:3) | 1,47±0,42 | 1,28±0,21 | 0,1092 |
| 595,3431 | .0334 | DG(32:6) | 9,44±1,86 | 9,77±2,26 | 0,7131 |
| 597,3656 | .0264 | DG(32:5) | 3,20±0,77 | 3,28±0,89 | 0,8331 |
| 599,4358 | .0280 | DG(32:4) | 1,79±0,56 | 1,76±0,47 | 0,8245 |
| 601,4881 | .0055 | DG(35:5) | 2,78±0,98 | 2,22±0,60 | 0,0725 |
| 602,4952 | .0173 | DG(34:6) | 1,58±0,36 | 1,36±0,26 | 0,0747 |
| 603,4974 | .0008 | DG(35:4) | 1,94±0,39 | 1,59±0,33 | 0,0128 |
| 605,4350 | .0196 | DG(32:1) | 1,58±0,37 | 1,34±0,23 | 0,4652 |
| 607,5433 | .0138 | DG(35:2) | 3,18±1,17 | 2,73±1,08 | 0,2749 |
| 608,5013 | .0235 | DG(34:3) | 2,67±0,59 | 2,24±0,62 | 0,0763 |
| 609,5570 | .0118 | DG(35:1) | 7,25±2,24 | 6,37±2,19 | 0,2801 |
| 610,5315 | .0089 | DG(34:2) | 4,43±1,20 | 3,83±1,23 | 0,2072 |
| 611,5564 | .0044 | DG(35:0) | 2,25±0,48 | 2,00±0,52 | 0,1948 |
| 613,4525 | .0073 | DG(O-34:4) | 1,83±0,35 | 2,10±0,73 | 0,3223 |
| 619,5233 | .0045 | DG(34:0) | 2,07±0,65 | 1,82±0,62 | 0,2666 |
| 621,5552 | .0100 | DG(36:2) | 7,62±2,67 | 6,27±2,09 | 0,1248 |
| 622,5616 | .0153 | DG(O-36:3) | 5,12±1,76 | 4,17±1,37 | 0,1042 |
| 623,5725 | .0116 | DG(36:1) | 7,95±2,33 | 6,82±1,60 | 0,1141 |
| 624,5742 | .0181 | DG(35:2) | 4,65±1,29 | 3,93±0,86 | 0,0730 |
| 625,5545 | .0144 | TG(35:0) | 2,98±0,53 | 2,60±0,49 | 0,0701 |
| 626,5616 | .0102 | DG(35:1) | 1,44±0,26 | 1,35±0,17 | 0,2777 |
| 627,4849 | .0115 | DG(35:3) | 1,62±0,35 | 1,55±0,33 | 0,6545 |
| 629,4197 | .0349 | DG(34:3) | 1,44±0,30 | 1,38±0,27 | 0,6660 |
| 631,4005 | .0334 | DG(36:8) | 1,31±0,40 | 1,13±0,18 | 0,1007 |
| 635,4583 | .0070 | TG(33:0) | 1,41±0,32 | 1,28±0,21 | 0,2064 |
| 637,5014 | .0188 | DG(38:8) | 1,90±0,47 | 1,67±0,36 | 0,1368 |
| 639,5569 | .0012 | TG(36:0) | 2,61±0,63 | 2,19±0,42 | 0,0923 |
| 640,5505 | .0005 | TG(35:1) | 1,46±0,27 | 1,31±0,17 | 0,0897 |
| 641,5478 | .0339 | DG(38:6) | 143±0,24 | 1,36±0,21 | 0,4347 |
| 647,3888 | .0190 | DG(36:8) | 1,33±0,48 | 1,24±0,36 | 0,5444 |
| 651,4760 | .0204 | DG(37:5) | 1,66±0,46 | 1,57±0,45 | 0,6587 |
| 652,4470 | .0465 | DG(38:9) | 1,25±0,43 | 1,15±0,33 | 0,5107 |
| 653,4877 | .0243 | DG(37:4) | 1,42±0,39 | 1,35±0,35 | 0,6412 |
| 655,5057 | .0220 | DG(37:3) | 1,41±0,33 | 1,28±0,24 | 0,2467 |
| 657,4830 | .0030 | DG(36:3) | 1,69±0,48 | 1,98±0,86 | 0,3847 |
| 659,4207 | .0444 | DG(38:8) | 1,17±0,41 | 1,21±0,41 | 0,8314 |
| 663,4539 | .0148 | DG(37:7) | 1,24±0,35 | 1,12±0,26 | 0,3125 |
| 665,4365 | .0182 | DG(37:6) | 1,13±0,38 | 1,01±0,23 | 0,2971 |
| 667,4325 | .0379 | DG(37:5) | 1,21±0,40 | 1,10±0,23 | 0,3122 |
| 669,4555 | .0305 | DG(37:4) | 1,29±0,46 | 1,16±0,31 | 0,3629 |
| 671,4636 | .0381 | DG(37:3) | 1,13±0,29 | 1,09±0,27 | 0,6999 |
| 673,4664 | .0143 | DG(39:8) | 1,09±0,28 | 1,09±0,25 | 0,9762 |
| 675,4455 | .0065 | DG(38:8) | 1,18±0,30 | 1,11±0,26 | 0,5151 |
| 677,4504 | .0043 | DG(38:7) | 1,15±0,41 | 1,06±0,26 | 0,4181 |
| 679,4556 | .0147 | DG(38:6) | 1,07±0,33 | 0,98±0,23 | 0,3758 |
| 683,4529 | .0123 | DG(40:10) | 1,11±0,32 | 1,01±0,19 | 0,2638 |
| 685,5643 | .0103 | DG(39:2) | 5,15±2,44 | 4,10±1,62 | 0,1498 |
| 703,5059 | .0219 | DG(41:7) | 1,48±0,59 | 1,74±0,75 | 0,3562 |
| 705,5411 | .0022 | DG(41:6) | 1,57±0,67 | 1,54±0,60 | 0,8914 |
| 706,4973 | .0431 | DG(42:10) | 1,11±0,37 | 1,06±0,32 | 0,7003 |
| 707,5001 | .0015 | DG(40:6) | 0,99±0,24 | 0,99±0,22 | 0,9376 |
| 709,4894 | .0087 | DG(42:11) | 0,89±0,19 | 0,94±0,18 | 0,5019 |
| 719,5068 | .0052 | DG(41:7) | 1,13±0,44 | 1,04±0,25 | 0,5004 |
| 721,5208 | .0035 | DG(41:6) | 1,03±0,27 | 1,01±0,23 | 0,8375 |
| 723,4884 | .0081 | TG(40:5) | 1,05±0,31 | 1,04±0,24 | 0,9122 |
| 725,4846 | .0276 | TG(40:4) | 0,98±0,31 | 0,94±0,22 | 0,693 |
| 727,5179 | .0099 | TG(40:3) | 1,01±0,30 | 0,96±0,19 | 0,5865 |
| 729,5039 | .0180 | DG(42:9) | 0,98±0,27 | 0,95±0,21 | 0,7553 |
| 731,5068 | .0051 | DG(42:8) | 1,01±0,22 | 1,00±0,18 | 0,9415 |
| 733,5160 | .0012 | DG(42:7) | 0,98±0,23 | 0,97±0,19 | 0,9049 |
| 735,5077 | .0112 | DG(44:12) | 0,93±0,24 | 0,93±0,18 | 0,9518 |
| 739,4897 | .0380 | DG(44:10) | 0,97±0,24 | 0,93±0,18 | 0,6635 |
| 745,5710 | .0038 | TG(41:1) | 1,55±0,45 | 1,99±0,95 | 0,2177 |
| 746,5846 | .0446 | TG(43:4) | 1,01±0,18 | 1,27±0,40 | 0,0885 |
| 749,5002 | .0119 | TG(42:6) | 0,94±0,19 | 0,91±0,16 | 0,6756 |
| 751,4994 | .0284 | TG(42:5) | 0,90±0,18 | 0,86±0,14 | 0,5532 |
| 753,5113 | .0322 | TG(42:4) | 0,97±0,24 | 0,91±0,15 | 0,4374 |
| 755,4964 | .0052 | DG(44:10) | 1,03±0,26 | 0,95±0,16 | 0,3691 |
| 756,6035 | .0101 | TG(44:6) | 2,05±0,54 | 2,02±0,52 | 0,8995 |
| 757,5916 | .0170 | DG(45:8) | 1,69±0,51 | 1,44±0,27 | *0,0221** |
| 758,5889 | .0404 | TG(44:5) | 1,34±0,27 | 1,23±0,21 | 0,2541 |
| 759,5314 | .0016 | DG(44:8) | 1,16±0,20 | 1,12±0,18 | 0,5578 |
| 761,5333 | .0153 | DG(44:7) | 1,03±0,14 | 1,14±0,26 | 0,2721 |
| 771,5817 | .0088 | TG(43:2) | 5,58±1,07 | 5,04±1,18 | 0,2397 |
| 773,6416 | .0008 | DG(44:1) | 4,73±1,41 | 4,35±1,04 | 0,4002 |
| 774,6391 | .0215 | TG(45:4) | 2,85±0,71 | 2,74±0,64 | 0,6618 |
| 775,6413 | .0168 | DG(44:0) | 2,41±0,53 | 2,26±0,46 | 0,4231 |
| 777,5492 | .0057 | TG(44:6) | 1,34±0,35 | 1,34±0,31 | 0,9597 |
| 779,5129 | .0463 | TG(44:5) | 1,04±0,20 | 1,03±0,16 | 0,8184 |
| 781,5357 | .0184 | DG(46:11) | 0,94±0,14 | 0,96±0,17 | 0,7154 |
| 783,5315 | .0015 | DG(46:10) | 1,06±0,19 | 1,04±0,14 | 0,7399 |
| 785,5528 | .0167 | TG(46:8) | 1,36±0,33 | 1,28±0,27 | 0,5349 |
| 787,5690 | .0047 | DG(46:8) | 1,75±0,42 | 1,65±0,39 | 0,5662 |
| 789,6067 | .0040 | TG(48:9) | 1,81±0,48 | 2,22±0,96 | 0,2529 |
| 791,5856 | .0100 | DG(46:6) | 1,15±0,21 | 1,19±0,23 | 0,5824 |
| 793,5367 | .0381 | TG(45:5) | 0,99±0,20 | 0,99±0,16 | 0,9436 |
| 795,5709 | .0195 | TG(45:4) | 0,99±0,18 | 0,94±0,15 | 0,4711 |
| 797,5713 | .0348 | TG(45:3) | 1,15±0,19 | 1,11±0,26 | 0,6742 |
| 799,6029 | .0188 | TG(45:2) | 1,32±0,30 | 1,24±0,31 | 0,4959 |
| 801,6088 | .0079 | TG(47:7) | 1,28±0,24 | 1,19±0,24 | 0,2995 |
| 803,5945 | .0010 | DG(47:7) | 1,42±0,32 | 1,27±0,25 | 0,1774 |
| 805,5816 | .0068 | TG(46:6) | 1,04±0,19 | 1,09±0,20 | 0,5025 |
| 807,5611 | .0294 | TG(46:5) | 0,95±0,13 | 0,93±0,13 | 0,7495 |
| 809,5946 | .0115 | TG(46:4) | 0,93±0,15 | 0,93±0,14 | 0,9653 |
| 811,6245 | .0028 | TG(46:3) | 1,01±0,16 | 0,98±0,13 | 0,6743 |
| 813,6508 | .0135 | TG(46:2) | 1,41±0,35 | 1,23±0,31 | 0,3112 |
| 814,6474 | .0130 | DG(50:12) | 1,10±0,25 | 1,03±0,22 | 0,4385 |
| 815,6414 | .0116 | TG(46:1) | 1,86±0,47 | 1,57±0,40 | 0,0802 |
| 816,6064 | .0436 | DG(50:11) | 1,26±0,19 | 1,15±0,23 | 0,2248 |
| 817,6313 | .0009 | TG(48:6) | 1,24±0,17 | 1,15±0,18 | 0,2132 |
| 819,6051 | .0146 | TG(47:6) | 1,07±0,15 | 1,02±0,15 | 0,4039 |
| 821,5700 | .0359 | DG(50:11) | 0,92±0,14 | 0,91±0,14 | 0,9298 |
| 825,6079 | .0294 | TG(47:3) | 0,87±0,12 | 0,88±0,12 | 0,9423 |
| 827,5979 | .0023 | DG(49:9) | 2,51±0,87 | 2,07±0,68 | 0,1082 |
| 829,6225 | .0096 | TG(49:7) | 1,29±0,22 | 1,21±0,23 | 0,3462 |
| 831,6452 | .0026 | TG(49:6) | 1,41±0,30 | 1,28±0,19 | 0,1443 |
| 832,6128 | .0321 | TG(50:10) | 1,05±0,13 | 0,97±0,13 | 0,1878 |
| 833,6331 | .0093 | DG(49:6) | 1,76±0,53 | 2,13±1,02 | 0,3227 |
| 834,6154 | .0452 | TG(50:9) | 1,12±0,22 | 1,13±0,46 | 0,2540 |
| 835,6166 | .0052 | TG(48:5) | 1,18±0,25 | 1,26±0,30 | 0,4279 |
| 839,6662 | .0131 | TG(48:3) | 0,99±0,21 | 0,93±0,11 | 0,3506 |
| 841,8128 | .0140 | DG(P-51:0) | 1,72±0,64 | 1,63±0,50 | 0,6991 |
| 843,8157 | .0358 | DG(52:3) | 1,80±0,59 | 1,80±0,59 | 0,9975 |
| 844,7548 | .0161 | TG(50:4) | 1,18±0,28 | 1,23±0,31 | 0,7169 |
| 845,7026 | .0009 | DG(53:9) | 1,11±0,22 | 1,13±0,25 | 0,8635 |
| 847,6135 | .0083 | TG(49:6) | 0,98±0,13 | 0,96±0,15 | 0,7086 |
| 853,6461 | .0140 | TG(51:9) | 0,91±0,15 | 0,94±0,20 | 0,5882 |
| 855,6731 | .0112 | DG(52:8) | 0,98±0,13 | 0,98±0,15 | 0,9019 |
| 857,7246 | .0247 | TG(49:1) | 1,19±0,27 | 1,13±0,22 | 0,4984 |
| 858,6885 | .0279 | TG(52:11) | 0,91±0,16 | 0,92±0,15 | 0,9513 |
| 859,6898 | .0089 | TG(53:9) | 1,03±0,15 | 0,99±0,15 | 0,5974 |
| 863,6219 | .0054 | TG(52:11) | 0,94±0,18 | 0,94±0,18 | 0,9432 |
| 865,7075 | .0024 | DG(51:4) | 0,96±0,19 | 0,96±0,16 | 0,8608 |
| 867,7192 | .0016 | DG(51:3) | 0,87±0,18 | 0,90±0,15 | 0,6172 |
| 869,6613 | .0022 | TG(52:8) | 0,79±0,11 | 0,81±0,11 | 0,6951 |
| 871,6061 | .0156 | TG(51:8) | 0,86±0,15 | 0,89±0,16 | 0,6359 |
| 873,6362 | .0012 | TG(51:7) | 0,86±0,13 | 0,97±0,19 | 0,1238 |
| 875,6320 | .0211 | TG(51:6) | 0,90±0,14 | 0,96±0,17 | 0,3858 |
| 877,6639 | .0047 | TG(51:5) | 1,58±0,59 | 1,97±1,14 | 0,3479 |
| 879,7784 | .0003 | DG(53:3) | 1,24±0,37 | 1,18±0,37 | 0,6749 |
| 880,8951 | .0103 | DG(O-54:0) | 0,88±0,24 | 0,84±0,22 | 0,6599 |
| 881,7970 | .0014 | DG(55:5) | 1,18±0,38 | 1,07±0,31 | 0,4715 |
| 883,6571 | .0011 | DG(53:9) | 0,84±0,14 | 0,85±0,14 | 0,8352 |
| 885,6206 | .0168 | TG(52:8) | 0,82±0,11 | 0,85±0,13 | 0,5147 |
| 887,6439 | .0092 | TG(52:7) | 0,81±0,12 | 0,84±0,13 | 0,5904 |
| 889,6570 | .0117 | TG(52:6) | 0,87±0,13 | 0,89±0,15 | 0,6787 |
| 891,6904 | .0061 | TG(52:5) | 0,99±0,16 | 1,04±0,21 | 0,5443 |
| 893,6927 | .0073 | TG(52:4) | 0,97±0,15 | 1,05±0,24 | 0,3414 |
| 895,6734 | .0057 | TG(54:9) | 0,82±0,14 | 0,86±0,14 | 0,4656 |
| 897,6930 | .0017 | TG(54:8) | 0,83±0,12 | 0,83±0,12 | 0,8877 |
| 899,6651 | .0120 | TG(53:8) | 0,80±0,13 | 0,81±0,12 | 0,8701 |
| 901,6510 | .0176 | TG(53:7) | 0,75±0,10 | 0,77±0,12 | 0,6395 |
| 903,6895 | .0051 | TG(53:6) | 0,85±0,14 | 0,83±0,13 | 0,7973 |
| 905,7185 | .0178 | DG(54:5) | 0,81±0,15 | 0,82±0,11 | 0,8523 |
| 907,7038 | .0117 | DG(56:10) | 0,80±0,13 | 0,83±0,13 | 0,5731 |
| 909,6504 | .0130 | TG(54:10) | 0,79±0,13 | 0,82±0,14 | 0,4858 |
| 911,6891 | .0003 | DG(55:9) | 0,78±0,12 | 0,79±0,12 | 0,8267 |
| 913,7431 | .0151 | TG(57:10) | 0,79±0,10 | 0,83±0,13 | 0,4684 |
| 915,6540 | .0061 | TG(56:13) | 0,79±0,12 | 0,83±0,12 | 0,4346 |
| 917,6568 | .0067 | TG(56:12) | 0,81±0,11 | 0,85±0,14 | 0,4757 |
| 933,6527 | .0153 | TG(56:12) | 0,77±0,11 | 0,77±0,11 | 0,8201 |
| 935,6416 | .0115 | TG(56:11) | 0,75±0,10 | 0,78±0,12 | 0,4964 |
| 937,6840 | .0153 | TG(56:10) | 0,78±0,11 | 0,88±0,19 | 0,2019 |
| 939,6762 | .0081 | TG(56:9) | 0,75±0,12 | 0,79±0,13 | 0,4011 |
| 941,6700 | .0066 | TG(58:14) | 0,73±0,11 | 0,75±0,11 | 0,7644 |
| 943,6472 | .0319 | TG(58:13) | 0,71±0,12 | 0,76±0,14 | 0,3412 |
| 945,6774 | .0173 | TG(58:12) | 0,76±0,12 | 0,76±0,12 | 0,9139 |
| 947,7007 | .0097 | TG(58:11) | 0,80±0,13 | 0,80±0,12 | 0,9946 |
| 949,7028 | .0022 | DG(58:11) | 0,77±0,11 | 0,76±0,11 | 0,9406 |
| 951,6913 | .0069 | TG(57:10) | 0,76±0,10 | 0,77±0,12 | 0,6891 |
| 953,6699 | .0301 | TG(57:9) | 0,73±0,10 | 0,76±0,11 | 0,5659 |
| 955,6708 | .0449 | TG(57:8) | 0,72±0,09 | 0,73±0,10 | 0,9076 |
| 957,6639 | .0265 | TG(58:14) | 0,71±0,08 | 0,72±0,10 | 0,8915 |
| 959,6997 | .0107 | TG(59:12) | 0,78±0,09 | 0,80±0,12 | 0,6088 |
| 961,6613 | .0073 | TG(58:12) | 0,81±0,10 | 0,85±0,15 | 0,4414 |
| 963,7685 | .0096 | DG(60:10) | 1,02±0,21 | 1,10±0,21 | 0,3617 |
| 964,7447 | .0060 | TG(60:14) | 0,88±0,16 | 0,90±0,14 | 0,7092 |
| 965,7599 | .0007 | TG(61:12) | 1,86±0,66 | 2,39±1,29 | 0,2576 |
| 966,7640 | .0096 | TG(60:13) | 1,22±0,38 | 1,55±0,73 | 0,2369 |
| 967,7583 | .0062 | DG(59:9) | 0,94±0,15 | 1,08±0,28 | 0,1979 |
| 968,7687 | .0014 | TG(60:12) | 0,74±0,10 | 0,80±0,12 | 0,2235 |
| 969,7049 | .0102 | TG(60:14) | 0,78±0,12 | 0,84±0,12 | 0,2302 |
| 973,6769 | .0335 | TG(60:13) | 0,74±0,10 | 0,75±0,12 | 0,7984 |
| 975,6612 | .0231 | TG(59:12) | 0,74±0,11 | 0,75±0,10 | 0,7370 |
| 977,6857 | .0143 | TG(59:11) | 0,73±0,09 | 0,75±0,11 | 0,6144 |
| 979,6765 | .0392 | TG(59:10) | 0,76±0,10 | 0,77±0,10 | 0,8233 |
| 981,6856 | .0456 | TG(59:9) | 0,75±0,11 | 0,85±0,20 | 0,1703 |
| 983,7246 | .0042 | TG(61:14) | 0,73±0,10 | 0,77±0,12 | 0,3799 |
| 987,6585 | .0259 | TG(60:13) | 0,72±0,09 | 0,75±0,15 | 0,5047 |
| 989,6659 | .0340 | TG(60:12) | 0,74±0,09 | 0,75±0,11 | 0,8120 |
| 991,7196 | .0039 | TG(60:11) | 0,75±0,09 | 0,76±0,12 | 0,8409 |
| 993,7475 | .0163 | TG(60:10) | 0,69±0,09 | 0,73±0,09 | 0,3320 |
| 995,7233 | .0128 | TG(62:15) | 0,71±0,08 | 0,75±0,10 | 0,3404 |
| 997,7172 | .0088 | TG(62:14) | 0,72±0,07 | 0,73±0,09 | 0,6864 |
| 999,6960 | .0116 | TG(61:14) | 0,68±0,09 | 0,71±0,09 | 0,4146 |

**Glycerophospholipids**

| Imput mass | Delta | Abbreviation | Ion | No OSA (n=16) | OSA (n=37) | p value |
| --- | --- | --- | --- | --- | --- | --- |
| 481,2930 | .0006 | PA(20:0) | [M+H]+ | 1,99±0,27 | 1,97±0,33 | 0,8142 |
| 519,0975 | .1513 | PA(20:0) | [M+K]+ | 1,98±0,09 | 1,98±0,12 | 0,9932 |
| 551,3662 | .0045 | PA(25:0) | [M+H]+ | 2,22±0,35 | 2,07±0,24 | 0,1687 |
| 607,4305 | .0028 | PA(29:0) | [M+H]+ | 2,02±0,30 | 1,85±0,24 | 0,0897 |
| 611,3529 | .0159 | PA(28:2) | [M+Na]+ | 1,43±0,10 | 1,41±0,10 | 0,6136 |
| 613,3808 | .0037 | PA(28:1) | [M+Na]+ | 2,46±0,37 | 2,44±0,48 | 0,8971 |
| 629,3630 | .0045 | PA(28:1) | [M+K]+ | 1,47±0,11 | 1,47±0,14 | 0,9815 |
| 639,3785 | .0216 | PA(30:2) | [M+Na]+ | 1,84±0,13 | 1,76±0,13 | 0,1501 |
| 645,3912 | .0015 | PA(29:0) | [M+K]+ | 1,25±0,11 | 1,26±0,13 | 0,9492 |
| 655,3728 | .0013 | PA(30:2) | [M+K]+ | 1,45±0,11 | 1,43±0,09 | 0,5288 |
| 657,4124 | .0227 | PA(30:1) | [M+K]+ | 2,28±0,37 | 2,25±0,49 | 0,8872 |
| 659,3870 | .0182 | PA(32:6) | [M+Na]+ | 1,22±0,09 | 1,19±0,09 | 0,4781 |
| 661,3782 | .0063 | PA(32:5) | [M+Na]+ | 1,21±0,09 | 1,18±0,10 | 0,5602 |
| 663,4945 | .0015 | PA(33:0) | [M+H]+ | 1,89±0,24 | 1,74±0,22 | 0,0702 |
| 673,3950 | .0260 | PA(31:0) | [M+K]+ | 1,15±0,11 | 1,15±0,10 | 0,9951 |
| 683,4397 | .0230 | PA(33:1) | [M+Na]+ | 1,19±0,11 | 1,13±0,09 | 0,1478 |
| 685,4887 | .0085 | PA(35:3) | [M+H]+ | 4,04±0,80 | 3,63±0,59 | 0,1003 |
| 687,4765 | .0194 | PA(35:2) | [M+H]+ | 1,44±0,16 | 1,31±0,18 | 0,0641* |
| 699,4253 | .0114 | PA(33:1) | [M+K]+ | 1,24±0,11 | 1,21±0,09 | 0,4126 |
| 701,4487 | .0034 | PA(P-36:6) | [M+Na]+ | 2,30±0,40 | 2,23±0,45 | 0,6855 |
| 703,4477 | .0163 | PA(35:5) | [M+Na]+ | 1,19±0,11 | 1,13±0,12 | 0,1670 |
| 705,4107 | .0365 | PA(35:4) | [M+Na]+ | 1,22±0,14 | 1,16±0,12 | 0,2297 |
| 717,4640 | .0151 | PA(38:8) | [M+H]+ | 1,17±0,17 | 1,11±0,17 | 0,3164 |
| 719,4813 | .0167 | PA(38:7) | [M+H]+ | 1,07±0,19 | 1,01±0,18 | 0,3571 |
| 721,5265 | .0117 | PA(P-37:3) | [M+Na]+ | 1,19±0,16 | 1,19±0,14 | 0,9935 |
| 723,5682 | .0377 | PA(P-37:1) | [M+Na]+ | 1,22±0,16 | 1,22±0,20 | 0,9779 |
| 725,5460 | .0000 | PA(P-37:0) | [M+Na]+ | 0,95±0,12 | 0,93±0,12 | 0,6062 |
| 727,4569 | .0110 | PA(P-38:6) | [M+Na]+ | 1,13±0,08 | 1,10±0,08 | 0,3418 |
| 733,4501 | .0283 | PA(37:4) | [M+Na]+ | 1,01±0,11 | 0,98±0,12 | 0,4327 |
| 735,4470 | .0103 | PA(36:4) | [M+K]+ | 1,05±0,08 | 1,00±0,11 | 0,2055 |
| 739,5037 | .0217 | PA(37:1) | [M+Na]+ | 0,92±0,15 | 0,89±0,15 | 0,6983 |
| 741,4564 | .0075 | PA(40:10) | [M+H]+ | 1,04±0,11 | 1,01±0,11 | 0,5944 |
| 743,4579 | .0048 | PA(38:6) | [M+Na]+ | 1,27±0,11 | 1,24±0,11 | 0,5079 |
| 745,4818 | .0015 | PA(40:8) | [M+H]+ | 2,07±0,34 | 2,04±0,39 | 0,8496 |
| 747,4902 | .0038 | PA(38:4) | [M+Na]+ | 1,17±0,15 | 1,12±0,15 | 0,3420 |
| 749,4275 | .0249 | PA(37:4) | [M+K]+ | 1,21±0,10 | 1,19±0,09 | 0,5774 |
| 751,4610 | .0070 | PA(37:3) | [M+K]+ | 0,96±0,08 | 0,95±0,09 | 0,7233 |
| 755,4553 | .0074 | PA(39:7) | [M+Na]+ | 0,96±0,08 | 0,96±0,09 | 0,8333 |
| 757,4926 | .0142 | PA(39:6) | [M+Na]+ | 0,95±0,12 | 0,91±0,13 | 0,3862 |
| 759,4868 | .0073 | PA(39:5) | [M+Na]+ | 1,07±0,11 | 1,03±0,13 | 0,3876 |
| 761,4524 | .0000 | PA(38:5) | [M+K]+ | 1,12±0,09 | 1,09±0,09 | 0,4630 |
| 769,5058 | .0092 | PA(38:1) | [M+K]+ | 1,02±0,16 | 0,95±0,14 | 0,1960 |
| 771,4962 | .0002 | PA(42:9) | [M+H]+ | 1,12±0,11 | 1,07±0,10 | 0,2993 |
| 773,4933 | .0165 | PA(40:5) | [M+Na]+ | 0,98±0,10 | 0,94±0,10 | 0,2996 |
| 777,4540 | .0296 | PA(39:4) | [M+K]+ | 0,99±0,07 | 0,96±0,08 | 0,4491 |
| 779,4273 | .0219 | PA(40:10) | [M+K]+ | 1,10±0,10 | 1,14±0,11 | 0,2832 |
| 781,5959 | .0218 | PA(42:4) | [M+H]+ | 1,09±0,14 | 1,05±0,16 | 0,5184 |
| 783,5913 | .0015 | PA(42:3) | [M+H]+ | 0,96±0,14 | 0,95±0,16 | 0,8674 |
| 785,4751 | .0228 | PA(40:7) | [M+K]+ | 0,97±0,07 | 0,94±0,09 | 0,3097 |
| 787,4756 | .0077 | PA(40:6) | [M+K]+ | 1,14±0,12 | 1,11±0,09 | 0,3913 |
| 789,5013 | .0176 | PA(40:5) | [M+K]+ | 1,86±0,31 | 1,84±0,38 | 0,9097 |
| 791,4900 | .0093 | PA(40:4) | [M+K]+ | 1,16±0,13 | 1,11±0,12 | 0,3128 |
| 795,4710 | .0231 | PA(42:8) | [M+Na]+ | 0,93±0,07 | 0,90±0,09 | 0,3340 |
| 797,4621 | .0477 | PA(42:7) | [M+Na]+ | 0,93±0,07 | 0,90±0,08 | 0,3763 |
| 799,4662 | .0018 | PA(41:7) | [M+K]+ | 0,92±0,07 | 0,92±0,09 | 0,9853 |
| 801,4695 | .0142 | PA(41:6) | [M+K]+ | 0,92±0,08 | 0,88±0,09 | 0,2294 |
| 803,4902 | .0090 | PA(41:5) | [M+K]+ | 0,98±0,09 | 0,93±0,11 | 0,2108 |
| 805,4615 | .0404 | PA(42:11) | [M+K]+ | 1,02±0,09 | 1,00±0,09 | 0,6741 |
| 807,4572 | .0205 | PA(42:10) | [M+K]+ | 1,01±0,07 | 1,00±0,08 | 0,5301 |
| 809,4273 | .0250 | PA(42:9) | [M+K]+ | 1,04±0,09 | 1,05±0,08 | 0,8104 |
| 811,4869 | .0189 | PA(42:8) | [M+K]+ | 0,85±0,09 | 0,83±0,09 | 0,4656 |
| 813,5093 | .0256 | PA(42:7) | [M+K]+ | 0,87±0,08 | 0,84±0,10 | 0,4086 |
| 815,4753 | .0126 | PA(44:12) | [M+Na]+ | 0,98±0,08 | 0,94±0,08 | 0,1303 |
| 817,5144 | .0005 | PA(42:5) | [M+K]+ | 0,89±0,09 | 0,84±0,11 | 0,2006 |
| 819,5060 | .0120 | PA(44:10) | [M+Na]+ | 0,90±0,09 | 0,85±0,10 | 0,2314 |
| 821,4699 | .0398 | PA(44:9) | [M+Na]+ | 0,88±0,06 | 0,87±0,08 | 0,6189 |
| 823,5495 | .0123 | PA(42:2) | [M+K]+ | 1,04±0,09 | 1,07±0,17 | 0,7133 |
| 825,5384 | .0027 | PA(44:7) | [M+Na]+ | 0,93±0,09 | 0,93±0,14 | 0,9424 |
| 827,4953 | .0039 | PA(43:7) | [M+K]+ | 0,85±0,07 | 0,83±0,10 | 0,4777 |
| 829,5040 | .0110 | PA(43:6) | [M+K]+ | 0,85±0,07 | 0,83±0,09 | 0,4800 |
| 831,5231 | .0075 | PA(43:5) | [M+K]+ | 1,04±0,11 | 0,98±0,12 | 0,2268 |
| 833,5292 | .0170 | PA(43:4) | [M+K]+ | 1,62±0,27 | 1,58±0,35 | 0,7610 |
| 835,5272 | .0347 | PA(43:3) | [M+K]+ | 1,01±0,13 | 0,96±0,11 | 0,3173 |
| 841,5395 | .0245 | PA(44:7) | [M+K]+ | 1,01±0,11 | 0,96±0,15 | 0,3527 |
| 843,5515 | .0209 | PA(44:6) | [M+K]+ | 0,89±0,09 | 0,86±0,10 | 0,3852 |
| 847,5057 | .0196 | PA(46:10) | [M+Na]+ | 0,85±0,08 | 0,84±0,08 | 0,5873 |
| 849,5037 | .0374 | PA(46:9) | [M+Na]+ | 0,86±0,08 | 0,85±0,09 | 0,5842 |
| 853,4823 | .0326 | PA(45:8) | [M+K]+ | 0,88±0,07 | 0,88±0,09 | 0,8828 |
| 855,5116 | .0190 | PA(45:7) | [M+K]+ | 0,90±0,07 | 0,88±0,08 | 0,3319 |
| 857,6273 | .0219 | PA(48:8) | [M+H]+ | 1,40±0,31 | 1,43±0,38 | 0,7695 |
| 859,5540 | .0078 | PA(45:5) | [M+K]+ | 0,99±0,08 | 0,95±0,08 | 0,2185 |
| 861,5236 | .0400 | PA(46:11) | [M+K]+ | 0,86±0,09 | 0,83±0,08 | 0,3599 |
| 863,5145 | .0152 | PA(46:10) | [M+K]+ | 0,85±0,09 | 0,83±0,08 | 0,4844 |
| 865,5313 | .0163 | PA(46:9) | [M+K]+ | 0,82±0,09 | 0,78±0,09 | 0,2308 |
| 867,6284 | .0039 | PA(45:1) | [M+K]+ | 1,02±0,16 | 1,05±0,24 | 0,6874 |
| 869,5542 | .0080 | PA(46:7) | [M+K]+ | 0,76±0,10 | 0,76±0,11 | 0,8939 |
| 871,5871 | .0253 | PA(46:6) | [M+K]+ | 0,88±0,12 | 0,90±0,15 | 0,7273 |
| 873,5367 | .0043 | PA(48:11) | [M+Na]+ | 0,88±0,07 | 0,86±0,09 | 0,4608 |
| 875,5368 | .0198 | PA(48:10) | [M+Na]+ | 1,03±0,08 | 0,99±0,09 | 0,3457 |
| 877,5576 | .0147 | PA(48:9) | [M+Na]+ | 1,57±0,26 | 1,54±0,31 | 0,7571 |
| 879,5411 | .0468 | PA(48:8) | [M+Na]+ | 0,94±0,10 | 0,90±0,08 | 0,2562 |
| 881,5091 | .0371 | PA(47:8) | [M+K]+ | 0,95±0,10 | 0,92±0,09 | 0,5772 |
| 883,6495 | .0063 | PA(46:0) | [M+K]+ | 1,72±0,41 | 1,72±0,52 | 0,9821 |
| 885,6708 | .0340 | PA(50:8) | [M+H]+ | 4,98±1,26 | 4,89±1,65 | 0,8782 |
| 887,6583 | .0059 | PA(50:7) | [M+H]+ | 1,31±0,27 | 1,33±0,35 | 0,8848 |
| 889,5356 | .0206 | PA(48:11) | [M+K]+ | 0,86±0,08 | 0,84±0,10 | 0,5177 |
| 891,5469 | .0164 | PA(48:10) | [M+K]+ | 0,89±0,07 | 0,86±0,09 | 0,4566 |
| 893,5484 | .0022 | PA(48:9) | [M+K]+ | 0,87±0,08 | 0,85±0,09 | 0,6592 |
| 895,5355 | .0263 | PA(48:8) | [M+K]+ | 0,83±0,07 | 0,83±0,07 | 0,9366 |
| 899,6216 | .0284 | PA(48:6) | [M+K]+ | 1,00±0,13 | 0,96±0,13 | 0,4206 |
| 901,6356 | .0268 | PA(48:5) | [M+K]+ | 1,18±0,18 | 1,22±0,29 | 0,6930 |
| 903,5922 | .0043 | PA(50:10) | [M+Na]+ | 0,93±0,08 | 0,89±0,09 | 0,3218 |
| 907,5189 | .0430 | PA(49:9) | [M+K]+ | 0,84±0,07 | 0,81±0,08 | 0,3186 |
| 909,5478 | .0297 | PA(49:8) | [M+K]+ | 0,78±0,08 | 0,77±0,08 | 0,5573 |
| 911,6390 | .0116 | PA(50:6) | [M+Na]+ | 0,77±0,09 | 0,76±0,11 | 0,8770 |
| 913,6341 | .0252 | PA(49:6) | [M+K]+ | 0,79±0,10 | 0,87±0,19 | 0,2879 |
| 915,5687 | .0382 | PA(50:12) | [M+K]+ | 0,82±0,08 | 0,78±0,09 | 0,2293 |
| 917,5841 | .0379 | PA(50:11) | [M+K]+ | 0,88±0,09 | 0,84±0,11 | 0,3173 |
| 919,5831 | .0213 | PA(50:10) | [M+K]+ | 1,01±0,11 | 0,96±0,11 | 0,2519 |
| 921,5936 | .0161 | PA(50:9) | [M+K]+ | 1,49±0,26 | 1,49±0,30 | 0,9837 |
| 923,5811 | .0121 | PA(50:8) | [M+K]+ | 0,90±0,09 | 0,89±0,08 | 0,6150 |
| 927,5462 | .0417 | PA(52:12) | [M+Na]+ | 0,76±0,07 | 0,74±0,07 | 0,5417 |
| 931,5812 | .0380 | PA(52:10) | [M+Na]+ | 0,81±0,09 | 0,77±0,08 | 0,2794 |
| 935,5734 | .0197 | PA(51:9) | [M+K]+ | 0,78±0,06 | 0,76±0,08 | 0,3800 |
| 937,5787 | .0302 | PA(51:8) | [M+K]+ | 0,82±0,07 | 0,79±0,08 | 0,3401 |
| 939,6188 | .0057 | PA(51:7) | [M+K]+ | 0,84±0,10 | 0,80±0,09 | 0,2166 |
| 943,5681 | .0062 | PA(52:12) | [M+K]+ | 0,72±0,06 | 0,71±0,07 | 0,6483 |
| 945,5606 | .0170 | PA(52:11) | [M+K]+ | 0,76±0,06 | 0,74±0,08 | 0,5234 |
| 947,5848 | .0083 | PA(52:10) | [M+K]+ | 0,82±0,07 | 0,80±0,07 | 0,3892 |
| 949,5798 | .0290 | PA(52:9) | [M+K]+ | 0,75±0,05 | 0,73±0,07 | 0,2717 |
| 951,5895 | .0350 | PA(52:8) | [M+K]+ | 0,74±0,06 | 0,72±0,07 | 0,4419 |
| 952,0066 | .1945 | PA(O-53:0) | [M+Na]+ | 1,62±0,18 | 1,63±0,23 | 0,9532 |
| 957,5891 | .0458 | PA(54:11) | [M+Na]+ | 0,69±0,07 | 0,67±0,07 | 0,5189 |
| 963,5907 | .0338 | PA(53:9) | [M+K]+ | 0,84±0,08 | 0,82±0,08 | 0,4336 |
| 965,6188 | .0214 | PA(53:8) | [M+K]+ | 1,44±0,17 | 1,44±0,21 | 0,9099 |
| 971,5620 | .0311 | PA(54:12) | [M+K]+ | 1,45±0,15 | 1,47±0,18 | 0,7541 |
| 973,5712 | .0377 | PA(54:11) | [M+K]+ | 1,32±0,13 | 1,31±0,16 | 0,8628 |
| 975,5879 | .0366 | PA(54:10) | [M+K]+ | 1,07±0,11 | 1,09±0,11 | 0,6369 |
| 546,3539 | .0367 | PE(20:0) | [M+Na]+ | 1,60±0,49 | 1,85±0,57 | 0,2425 |
| 552,3675 | .0015 | PE(22:0) | [M+H]+ | 1,52±0,37 | 1,97±0,60 | 0,0711 |
| 566,3638 | .0178 | PE(23:0) | [M+H]+ | 1,09±0,17 | 1,16±0,24 | 0,4458 |
| 574,3449 | .0035 | PE(22:0) | [M+Na]+ | 1,52±0,53 | 2,26±0,86 | 0,0720 |
| 608,4556 | .0271 | PE(26:0) | [M+H]+ | 1,17±0,24 | 1,11±0,17 | 0,4436 |
| 614,4396 | .0235 | PE(P-26:0) | [M+Na]+ | 1,55±0,26 | 1,60±0,32 | 0,6206 |
| 622,4953 | .0148 | PE(O-28:0) | [M+H]+ | 1,05±0,29 | 1,03±0,20 | 0,8171 |
| 630,4239 | .0110 | PE(28:3) | [M+H]+ | 1,15±0,25 | 1,15±0,15 | 0,9976 |
| 632,4120 | .0165 | PE(28:2) | [M+H]+ | 1,09±0,21 | 1,05±0,15 | 0,6024 |
| 634,4505 | .0063 | PE(28:1) | [M+H]+ | 1,04±0,25 | 1,02±0,25 | 0,8548 |
| 636,4468 | .0130 | PE(28:0) | [M+H]+ | 1,10±0,28 | 1,06±0,21 | 0,6613 |
| 640,4217 | .0263 | PE(27:2) | [M+Na]+ | 1,19±0,22 | 1,26±0,16 | 0,2658 |
| 642,4310 | .0200 | PE(27:1) | [M+Na]+ | 1,07±0,21 | 1,06±0,17 | 0,8332 |
| 644,4881 | .0251 | PE(O-28:0) | [M+Na]+ | 1,11±0,34 | 1,01±0,24 | 0,2825 |
| 646,5039 | .0233 | PE(P-30:2) | [M+H]+ | 1,02±0,29 | 1,02±0,22 | 0,9745 |
| 656,4221 | .0046 | PE(28:1) | [M+Na]+ | 1,12±0,19 | 1,17±0,15 | 0,4254 |
| 658,4695 | .0253 | PE(30:3) | [M+H]+ | 1,55±0,27 | 1,66±0,31 | 0,3020 |
| 660,4276 | .0322 | PE(30:2) | [M+H]+ | 1,13±0,23 | 1,15±0,19 | 0,7659 |
| 662,4466 | .0289 | PE(30:1) | [M+H]+ | 1,14±0,26 | 1,17±0,21 | 0,6397 |
| 664,5442 | .0167 | PE(O-31:0) | [M+H]+ | 1,39±0,25 | 1,27±0,20 | 0,1893 |
| 668,4343 | .0076 | PE(29:2) | [M+Na]+ | 1,05±0,26 | 1,03±0,18 | 0,7938 |
| 670,4513 | .0071 | PE(31:4) | [M+H]+ | 1,05±0,21 | 1,03±0,18 | 0,7799 |
| 672,4632 | .0034 | PE(31:3) | [M+H]+ | 1,08±0,20 | 1,10±0,19 | 0,7862 |
| 674,4546 | .0209 | PE(31:2) | [M+H]+ | 1,12±0,24 | 1,13±0,18 | 0,8643 |
| 676,4567 | .0344 | PE(31:1) | [M+H]+ | 1,08±0,25 | 1,08±0,21 | 0,9781 |
| 682,4341 | .0082 | PE(30:2) | [M+Na]+ | 1,10±0,27 | 1,15±0,24 | 0,5805 |
| 684,4346 | .0234 | PE(30:1) | [M+Na]+ | 1,08±0,23 | 1,12±0,19 | 0,5521 |
| 686,5432 | .0314 | PE(P-33:3) | [M+H]+ | 1,68±0,57 | 1,73±0,37 | 0,8017 |
| 688,4458 | .0138 | PE(29:0) | [M+K]+ | 1,09±0,20 | 1,11±0,16 | 0,7776 |
| 696,5031 | .0069 | PE(P-34:5) | [M+H]+ | 1,08±0,26 | 1,05±0,20 | 0,7220 |
| 698,5406 | .0287 | PE(P-34:4) | [M+H]+ | 1,03±0,28 | 0,94±0,21 | 0,3093 |
| 700,5299 | .0024 | PE(P-34:2) | [M+H]+ | 1,31±0,48 | 1,18±0,24 | 0,2901 |
| 702,5169 | .0101 | PE(33:2) | [M+H]+ | 1,52±0,24 | 1,61±0,30 | 0,4112 |
| 704,5369 | .0145 | PE(33:1) | [M+H]+ | 1,10±0,29 | 1,32±0,29 | 0,0596 |
| 706,4649 | .0226 | PE(32:4) | [M+Na]+ | 1,11±0,22 | 1,20±0,23 | 0,3554 |
| 708,5335 | .0392 | PE(P-33:3) | [M+Na]+ | 1,08±0,25 | 1,04±0,22 | 0,6099 |
| 710,4474 | .0262 | PE(32:2) | [M+Na]+ | 1,23±0,30 | 1,17±0,26 | 0,5732 |
| 712,4707 | .0186 | PE(32:1) | [M+Na]+ | 1,12±0,24 | 1,12±0,26 | 0,9918 |
| 714,4680 | .0204 | PE(31:1) | [M+K]+ | 1,06±0,21 | 1,10±0,15 | 0,6453 |
| 718,5119 | .0262 | PE(34:1) | [M+H]+ | 1,08±0,23 | 1,08±0,17 | 0,9724 |
| 720,5492 | .0045 | PE(34:0) | [M+H]+ | 1,12±0,30 | 1,17±0,20 | 0,6064 |
| 726,5829 | .0398 | PE(P-36:3) | [M+H]+ | 1,01±0,31 | 1,04±0,23 | 0,7599 |
| 730,5582 | .0201 | PE(35:2) | [M+H]+ | 1,13±0,30 | 1,32±0,32 | 0,1308 |
| 732,5772 | .0235 | PE(35:1) | [M+H]+ | 1,15±0,36 | 1,42±0,41 | 0,0917 |
| 734,5320 | .0374 | PE(35:0) | [M+H]+ | 1,03±0,25 | 1,05±0,17 | 0,8414 |
| 736,5291 | .0035 | PE(P-35:3) | [M+Na]+ | 1,06±0,26 | 1,05±0,18 | 0,9499 |
| 738,6147 | .0370 | PE(P-36:0) | [M+Na]+ | 1,29±0,55 | 1,50±0,48 | 0,2680 |
| 742,5344 | .0018 | PE(34:0) | [M+Na]+ | 1,10±0,27 | 1,10±0,19 | 0,9559 |
| 744,5754 | .0217 | PE(36:2) | [M+H]+ | 1,30±0,48 | 1,60±0,48 | 0,0874 |
| 746,5944 | .0250 | PE(36:5) | [M+H]+ | 1.81 ± 0.72 | 2.51 ± 1.05 | 0.0232* |
| 748,6077 | .0227 | PE(36:0) | [M+H]+ | 1,40±0,58 | 1,93±0,75 | 0,0668 |
| 754,5864 | .0120 | PE(P-38:4) | [M+H]+ | 1,09±0,37 | 1,19±0,30 | 0,3857 |
| 756,5773 | .0109 | PE(O-36:0) | [M+Na]+ | 1,07±0,32 | 1,20±0,28 | 0,2320 |
| 760,6124 | .0090 | PE(P-38:1) | [M+H]+ | 1,51±0,60 | 1,84±0,64 | 0,1474 |
| 762,6014 | .0007 | PE(37:0) | [M+H]+ | 1,61±0,75 | 2,11±0,86 | 0,0996 |
| 768,6249 | .0367 | PE(O-37:1) | [M+Na]+ | 1,10±0,89 | 2,46±1,16 | 0,0526 |
| 770,6146 | .0452 | PE(38:5) | [M+Na]+ | 1.62 ± 1.31 | 2.70 ± 1.61 | 0.0192* |
| 772,6295 | .0444 | PE(37:2) | [M+K]+ | 1.90 ± 1.31 | 2.90 ± 1.73 | 0.0242* |
| 774,6558 | .0188 | PE(35:1) | [M+K]+ | 1.50 ± 0.92 | 2.21 ± 1.12 | 0.0181* |
| 776,6470 | .0057 | PE(O-39:0) | [M+H]+ | 1,28±0,52 | 1,60±0,56 | 0,1245 |
| 778,6040 | .0296 | PE(O-40:6) | [M+H]+ | 1,13±0,35 | 1,21±0,27 | 0,4967 |
| 784,6141 | .0073 | PE(O-40:3) | [M+H]+ | 1,67±0,73 | 2,08±0,83 | 0,1492 |
| 786,6360 | .0010 | PE(P-40:2) | [M+H]+ | 1,90±0,88 | 2,38±1,03 | 0,1792 |
| 788,6292 | .0129 | PE(39:1) | [M+H]+ | 2,15±1,07 | 2,87±1,31 | 0,1097 |
| 790,6385 | .0064 | PE(39:0) | [M+H]+ | 3,38±0,95 | 3,27±1,36 | 0,0561 |
| 794,6124 | .0279 | PE(38:6) | [M+Na]+ | 1.70 ± 1.11 | 2.63 ± 1.53 | 0.0323* |
| 796,6538 | .0342 | PE(37:3) | [M+Na]+ | 1.71 ± 0.91 | 2.50 ± 1.31 | 0.0185* |
| 798,6414 | .0062 | PE(O-39:0) | [M+Na]+ | 1,38±0,64 | 1,96±0,88 | 0,0584 |
| 800,6193 | .0029 | PE(40:2) | [M+H]+ | 1,15±0,45 | 1,42±0,48 | 0,1317 |
| 802,5967 | .0241 | PE(O-40:5) | [M+Na]+ | 1,15±0,38 | 1,32±0,33 | 0,2416 |
| 804,6336 | .0140 | PE(40:0) | [M+H]+ | 1,32±0,41 | 1,45±0,37 | 0,4126 |
| 806,6255 | .0197 | PE(P-42:6) | [M+H]+ | 1,34±0,42 | 1,55±0,48 | 0,2379 |
| 808,6321 | .0106 | PE(P-42:5) | [M+H]+ | 1,79±0,68 | 2,14±0,80 | 0,2135 |
| 810,6360 | .0007 | PE(O-40:1) | [M+Na]+ | 1,62±0,69 | 2,05±0,81 | 0,1250 |
| 812,6418 | .0090 | PE(O-40:0) | [M+Na]+ | 2,18±1,18 | 3,10±1,46 | 0,0654 |
| 814,6432 | .0112 | PE(41:2) | [M+H]+ | 1,54±0,80 | 2,06±0,91 | 0,0989 |
| 816,6307 | .0170 | PE(41:1) | [M+H]+ | 1,15±0,44 | 1,38±0,41 | 0,1436 |
| 818,6342 | .0291 | PE(41:0) | [M+H]+ | 1,14±0,41 | 1,44±0,42 | 0,0566 |
| 820,6306 | .0456 | PE(39:4) | [M+K]+ | 1.21 ± 0.61 | 1.60 ± 0.70 | 0.0401* |
| 822,6367 | .0015 | PE(P-41:1) | [M+Na]+ | 1,06±0,49 | 1,38±0,47 | 0,0553 |
| 824,6429 | .0079 | PE(O-41:1) | [M+Na]+ | 1,31±0,40 | 1,44±0,36 | 0,3667 |
| 826,6126 | .0175 | PE(40:0) | [M+Na]+ | 1,07±0,36 | 1,17±0,30 | 0,4246 |
| 828,6069 | .0185 | PE(39:0) | [M+K]+ | 1,24±0,46 | 1,38±0,38 | 0,4188 |
| 830,6123 | .0084 | PE(O-42:5) | [M+Na]+ | 1,10±0,36 | 1,18±0,29 | 0,5093 |
| 832,6216 | .0021 | PE(O-42:4) | [M+Na]+ | 1,09±0,29 | 1,16±0,21 | 0,4898 |
| 834,6157 | .0150 | PE(43:6) | [M+H]+ | 1,42±0,29 | 1,63±0,31 | 0,0784 |
| 836,6366 | .0202 | PE(43:5) | [M+H]+ | 1,38±0,62 | 1,75±0,62 | 0,1224 |
| 838,6348 | .0027 | PE(43:4) | [M+H]+ | 1,13±0,42 | 1,38±0,42 | 0,1239 |
| 840,6423 | .0036 | PE(41:0) | [M+Na]+ | 1,12±0,31 | 1,25±0,29 | 0,2668 |
| 842,5969 | .0071 | PE(40:0) | [M+K]+ | 0,94±0,23 | 0,98±0,19 | 0,6260 |
| 844,5894 | .0044 | PE(44:8) | [M+H]+ | 0,93±0,26 | 0,95±0,18 | 0,7735 |
| 846,5957 | .0032 | PE(42:4) | [M+Na]+ | 0,97±0,25 | 0,98±0,16 | 0,8238 |
| 848,5947 | .0198 | PE(42:3) | [M+Na]+ | 0,97±0,26 | 0,99±0,18 | 0,7614 |
| 850,5928 | .0201 | PE(41:3) | [M+K]+ | 0,98±0,25 | 1,03±0,19 | 0,5499 |
| 852,6006 | .0121 | PE(41:2) | [M+K]+ | 1,00±0,31 | 1,11±0,25 | 0,3754 |
| 854,5986 | .0310 | PE(43:7) | [M+Na]+ | 0,98±0,26 | 1,03±0,22 | 0,5710 |
| 856,6036 | .0204 | PE(43:6) | [M+Na]+ | 0,95±0,24 | 1,00±0,17 | 0,4934 |
| 858,6039 | .0032 | PE(45:8) | [M+H]+ | 0,94±0,23 | 0,95±0,17 | 0,8935 |
| 860,6044 | .0102 | PE(43:4) | [M+Na]+ | 0,96±0,25 | 0,99±0,18 | 0,6709 |
| 862,5900 | .0172 | PE(42:4) | [M+K]+ | 0,92±0,26 | 0,96±0,18 | 0,5673 |
| 864,5924 | .0040 | PE(42:3) | [M+K]+ | 0,92±0,25 | 0,94±0,17 | 0,7436 |
| 866,5883 | .0157 | PE(42:2) | [M+K]+ | 0,86±0,22 | 0,89±0,15 | 0,5463 |
| 868,5936 | .0086 | PE(46:10) | [M+H]+ | 0,88±0,22 | 0,90±0,16 | 0,7897 |
| 870,5941 | .0047 | PE(44:6) | [M+Na]+ | 0,89±0,23 | 0,90±0,15 | 0,9109 |
| 872,5776 | .0205 | PE(43:6) | [M+K]+ | 0,91±0,23 | 0,94±0,17 | 0,7025 |
| 874,6000 | .0301 | PE(44:4) | [M+Na]+ | 0,94±0,25 | 0,98±0,16 | 0,6369 |
| 876,6208 | .0250 | PE(44:3) | [M+Na]+ | 1,02±0,25 | 1,06±0,18 | 0,6549 |
| 878,6410 | .0204 | PE(44:2) | [M+Na]+ | 1,21±0,22 | 1,36±0,27 | 0,1414 |
| 880,6059 | .0227 | PE(45:8) | [M+Na]+ | 0,89±0,23 | 0,95±0,16 | 0,3948 |
| 882,6110 | .0122 | PE(45:7) | [M+Na]+ | 0,95±0,21 | 1,00±0,17 | 0,5066 |
| 884,6920 | .0182 | PE(46:2) | [M+H]+ | 1,08±0,31 | 1,14±0,21 | 0,5296 |
| 886,5796 | .0068 | PE(44:6) | [M+K]+ | 0,90±0,21 | 0,91±0,14 | 0,9329 |
| 890,6168 | .0127 | PE(44:4) | [M+K]+ | 0,90±0,27 | 0,92±0,15 | 0,8007 |
| 892,6228 | .0030 | PE(44:3) | [M+K]+ | 0,94±0,24 | 0,94±0,15 | 0,9444 |
| 894,6154 | .0147 | PE(48:11) | [M+H]+ | 0,91±0,22 | 0,94±0,15 | 0,6294 |
| 896,6281 | .0118 | PE(48:10) | [M+H]+ | 0,94±0,24 | 0,98±0,18 | 0,6610 |
| 898,6261 | .0040 | PE(46:6) | [M+Na]+ | 0,96±0,25 | 1,06±0,19 | 0,2007 |
| 900,6572 | .0096 | PE(48:8) | [M+H]+ | 0,92±0,26 | 0,99±0,21 | 0,4489 |
| 902,6344 | .0270 | PE(46:4) | [M+Na]+ | 0,92±0,26 | 1,00±0,19 | 0,3334 |
| 904,6477 | .0294 | PE(46:3) | [M+Na]+ | 0,98±0,30 | 1,08±0,23 | 0,2993 |
| 906,6402 | .0049 | PE(45:3) | [M+K]+ | 0,91±0,27 | 0,96±0,19 | 0,5492 |
| 908,6309 | .0164 | PE(47:8) | [M+Na]+ | 0,88±0,25 | 0,96±0,19 | 0,3782 |
| 910,6360 | .0058 | PE(47:7) | [M+Na]+ | 0,85±0,21 | 0,91±0,16 | 0,4083 |
| 912,6499 | .0023 | PE(49:9) | [M+H]+ | 0,82±0,22 | 0,89±0,18 | 0,3254 |
| 914,6410 | .0204 | PE(47:5) | [M+Na]+ | 0,85±0,23 | 0,91±0,17 | 0,4557 |
| 916,6406 | .0209 | PE(46:5) | [M+K]+ | 0,90±0,25 | 0,96±0,19 | 0,4175 |
| 918,6454 | .0100 | PE(46:4) | [M+K]+ | 0,95±0,26 | 1,03±0,20 | 0,3543 |
| 920,6588 | .0077 | PE(46:3) | [M+K]+ | 1,04±0,34 | 1,16±0,23 | 0,2234 |
| 922,6782 | .0116 | PE(46:2) | [M+K]+ | 1,24±0,25 | 1,45±0,30 | 0,0663 |
| 924,6580 | .0104 | PE(50:10) | [M+H]+ | 0,98±0,34 | 1,17±0,29 | 0,0948 |
| 926,6564 | .0050 | PE(48:6) | [M+Na]+ | 0,99±0,32 | 1,19±0,28 | 0,0804 |
| 928,6654 | .0118 | PE(48:5) | [M+Na]+ | 0,88±0,28 | 1,01±0,24 | 0,1688 |
| 930,6486 | .0133 | PE(47:5) | [M+K]+ | 0,92±0,27 | 1,01±0,22 | 0,2863 |
| 932,6700 | .0189 | PE(47:4) | [M+K]+ | 0,94±0,31 | 1,04±0,25 | 0,3293 |
| 934,6502 | .0201 | PE(49:9) | [M+Na]+ | 0,85±0,27 | 0,98±0,20 | 0,1402 |
| 936,6463 | .0005 | PE(49:8) | [M+Na]+ | 0,88±0,16 | 0,96±0,19 | 0,3234 |
| 938,6399 | .0215 | PE(49:7) | [M+Na]+ | 0,88±0,25 | 1,02±0,20 | 0,0984 |
| 940,6778 | .0007 | PE(49:6) | [M+Na]+ | 0,90±0,28 | 0,99±0,20 | 0,3357 |
| 942,6479 | .0125 | PE(48:6) | [M+K]+ | 0,86±0,27 | 1,04±0,24 | 0,0762 |
| 944,6634 | .0124 | PE(48:5) | [M+K]+ | 0,93±0,31 | 1,08±0,27 | 0,1498 |
| 946,6641 | .0025 | PE(48:4) | [M+K]+ | 0,94±0,31 | 1,08±0,27 | 0,1880 |
| 948,6708 | .0232 | PE(52:12) | [M+H]+ | 1,02±0,40 | 1,23±0,32 | 0,1121 |
| 950,6700 | .0067 | PE(52:11) | [M+H]+ | 0,90±0,29 | 1,03±0,24 | 0,1591 |
| 952,6534 | .0237 | PE(50:7) | [M+Na]+ | 0,84±0,25 | 0,93±0,19 | 0,2459 |
| 954,6368 | .0015 | PE(49:7) | [M+K]+ | 0,82±0,23 | 0,89±0,21 | 0,3565 |
| 956,6458 | .0053 | PE(49:6) | [M+K]+ | 0,80±0,24 | 0,90±0,20 | 0,2127 |
| 958,6539 | .0128 | PE(49:5) | [M+K]+ | 0,82±0,28 | 0,94±0,22 | 0,1671 |
| 960,6491 | .0332 | PE(49:4) | [M+K]+ | 0,87±0,27 | 0,95±0,21 | 0,2855 |
| 962,6559 | .0055 | PE(51:9) | [M+Na]+ | 0,83±0,28 | 0,98±0,22 | 0,1296 |
| 964,6596 | .0175 | PE(51:8) | [M+Na]+ | 0,90±0,30 | 1,08±0,27 | 0,0895 |
| 966,6975 | .0048 | PE(51:7) | [M+Na]+ | 1,08±0,22 | 1,31±0,33 | 0,0558 |
| 968,6529 | .0018 | PE(50:7) | [M+K]+ | 0,77±0,24 | 0,90±0,18 | 0,0875 |
| 970,6373 | .0071 | PE(52:12) | [M+Na]+ | 0,79±0,25 | 0,96±0,22 | 0,0562 |
| 972,6614 | .0156 | PE(52:11) | [M+Na]+ | 0,80±0,26 | 0,92±0,23 | 0,1989 |
| 974,6650 | .0036 | PE(52:10) | [M+Na]+ | 0,79±0,25 | 0,88±0,20 | 0,2667 |
| 976,6397 | .0374 | PE(52:9) | [M+Na]+ | 0,75±0,22 | 0,82±0,17 | 0,2869 |
| 978,6191 | .0163 | PE(51:9) | [M+K]+ | 0,75±0,24 | 0,84±0,18 | 0,2105 |
| 980,6321 | .0190 | PE(51:8) | [M+K]+ | 0,78±0,25 | 0,87±0,22 | 0,2570 |
| 982,6460 | .0207 | PE(51:7) | [M+K]+ | 0,81±0,26 | 0,93±0,24 | 0,2084 |
| 984,6328 | .0496 | PE(51:6) | [M+K]+ | 0,73±0,22 | 0,80±0,19 | 0,3205 |
| 986,6489 | .0448 | PE(52:12) | [M+K]+ | 0,72±0,22 | 0,84±0,21 | 0,1406 |
| 988,6603 | .0405 | PE(52:11) | [M+K]+ | 0,72±0,25 | 0,88±0,24 | 0,0739 |
| 990,6412 | .0058 | PE(52:10) | [M+K]+ | 0,71±0,24 | 0,79±0,19 | 0,2566 |
| 992,6371 | .0139 | PE(52:9) | [M+K]+ | 0,68±0,20 | 0,76±0,16 | 0,2772 |
| 994,6194 | .0472 | PE(52:8) | [M+K]+ | 0,65±0,21 | 0,73±0,16 | 0,1963 |
| 998,6139 | .0475 | PE(54:12) | [M+Na]+ | 0,64±0,21 | 0,72±0,16 | 0,2772 |
| 408,1174 | .0379 | LPE(10:0) | [M+K]+ | 2,28±0,36 | 2,30±0,33 | 0,8687 |
| 462,1554 | .0469 | LPE(14:1) | [M+K]+ | 2,08±0,25 | 2,09±0,28 | 0,9296 |
| 482,3476 | .0436 | LPE(18:0) | [M+H]+ | 1,44±0,19 | 1,44±0,26 | 0,9175 |
| 564,3525 | .0498 | LPE(24:1) | [M+H]+ | 1,66±0,42 | 1,44±0,36 | 0,1801 |
| 566,3723 | .0456 | LPE(24:0) | [M+H]+ | 1,48±0,34 | 1,34±0,24 | 0,2596 |
| 578,2919 | .0303 | LPE(24:5) | [M+Na]+ | 1,49±0,24 | 1,41±0,23 | 0,3801 |
| 582,3746 | .0193 | LPE(26:6) | [M+H]+ | 1,34±0,19 | 1,27±0,16 | 0,3385 |
| 608,4122 | .0431 | LPE(26:4) | [M+Na]+ | 1,19±0,21 | 1,12±0,15 | 0,3572 |
| 610,3970 | .0104 | LPE(28:6) | [M+H]+ | 1,20±0,21 | 1,16±0,15 | 0,5679 |
| 628,4775 | .0458 | LPE(27:1) | [M+Na]+ | 1,39±0,30 | 1,35±0,30 | 0,7114 |
| 630,4548 | .0056 | LPE(29:3) | [M+H]+ | 1,17±0,17 | 1,17±0,20 | 0,9554 |
| 650,5523 | .0405 | LPE(30:0) | [M+H]+ | 1,06±0,15 | 1,10±0,15 | 0,5935 |
| 658,1151 | .0327 | LPE(29:0) | [M+Na]+ | 1,60±0,27 | 1,76±0,38 | 0,2211 |
| 660,4466 | .0097 | LPE(28:0) | [M+K]+ | 1,11±0,18 | 1,11±0,14 | 0,9142 |
| 672,4939 | .0004 | LPE(30:0) | [M+Na]+ | 1,14±0,18 | 1,13±0,18 | 0,8887 |
| 674,4715 | .0188 | LPE(29:0) | [M+K]+ | 1,15±0,18 | 1,14±0,21 | 0,9051 |
| 700,5578 | .0322 | LPE(32:0) | [M+Na]+ | 1,24±0,23 | 1,16±0,22 | 0,4139 |
| 720,5116 | .0173 | LPE(34:4) | [M+Na]+ | 1,04±0,16 | 1,04±0,16 | 0,9626 |
| 728,5252 | .0317 | LPE(34:0) | [M+Na]+ | 1,06±0,16 | 1,05±0,15 | 0,7761 |
| 730,5003 | .0149 | LPE(33:0) | [M+K]+ | 1,08±0,15 | 1,04±0,14 | 0,5291 |
| 744,5345 | .0036 | LPE(34:0) | [M+K]+ | 1,19±0,17 | 1,22±0,21 | 0,6759 |
| 652,4224 | .0041 | PS(26:0) | [M+H]+ | 0,98±0,11 | 1,04±0,07 | 0,0759 |
| 660,4423 | .0207 | PS(O-26:0) | [M+Na]+ | 0,90±0,08 | 0,96±0,09 | 0,0897 |
| 662,4286 | .0259 | PS(27:2) | [M+H]+ | 0,97±0,07 | 1,02±0,08 | 0,0862 |
| 674,4410 | .0383 | PS(28:3) | [M+H]+ | 0,93±0,07 | 0,96±0,06 | 0,1712 |
| 676,4396 | .0212 | PS(28:2) | [M+H]+ | 0,87±0,08 | 0,93±0,07 | 0,0663 |
| 700,4905 | .0376 | PS(P-29:0) | [M+Na]+ | 0,94±0,08 | 1,01±0,09 | 0,0661 |
| 702,5069 | .0383 | PS(O-29:0) | [M+Na]+ | 1,29±0,11 | 1,46±0,22 | 0,0732 |
| 704,4640 | .0144 | PS(30:2) | [M+H]+ | 0,85±0,05 | 0,92±0,06 | 0,1006 |
| 706,4562 | .0092 | PS(30:1) | [M+H]+ | 0,93±0,08 | 0,97±0,08 | 0,1328 |
| 708,5167 | .0006 | PS(O-31:0) | [M+H]+ | 0,87±0,06 | 0,87±0,06 | 0,8059 |
| 712,4544 | .0015 | PS(P-30:1) | [M+Na]+ | 0,88±0,07 | 0,93±0,07 | 0,0902 |
| 714,4675 | .0010 | PS(P-30:1) | [M+Na]+ | 0,94±0,07 | 0,97±0,06 | 0,3306 |
| 716,4757 | .0261 | PS(31:3) | [M+H]+ | 1,04±0,10 | 1,09±0,08 | 0,1776 |
| 718,4796 | .0143 | PS(31:2) | [M+H]+ | 0,89±0,07 | 0,94±0,07 | 0,1473 |
| 720,4644 | .0165 | PS(31:1) | [M+H]+ | 0,90±0,06 | 0,93±0,07 | 0,2256 |
| 722,4705 | .0261 | PS(31:0) | [M+H]+ | 0,99±0,07 | 1,04±0,08 | 0,1931 |
| 726,4687 | .0347 | PS(32:5) | [M+H]+ | 0,86±0,08 | 0,90±0,06 | 0,1519 |
| 728,4825 | .0017 | PS(O-31:1) | [M+Na]+ | 0,87±0,06 | 0,92±0,06 | 0,0697 |
| 730,4653 | .0000 | PS(32:3) | [M+H]+ | 0,91±0,06 | 0,94±0,07 | 0,2072 |
| 732,4661 | .0149 | PS(32:2) | [M+H]+ | 0,89±0,06 | 0,93±0,07 | 0,1880 |
| 734,4739 | .0227 | PS(32:1) | [M+H]+ | 0,87±0,08 | 0,91±0,06 | 0,1233 |
| 736,4814 | .0308 | PS(32:0) | [M+H]+ | 0,89±0,07 | 0,93±0,06 | 0,1619 |
| 738,4767 | .0446 | PS(31:3) | [M+Na]+ | 0,91±0,07 | 0,95±0,08 | 0,1840 |
| 740,4879 | .0019 | PS(P-34:4) | [M+H]+ | 1,06±0,13 | 1,14±0,10 | 0,0844 |
| 742,4824 | .0171 | PS(33:4) | [M+H]+ | 0,94±0,08 | 0,99±0,07 | 0,1277 |
| 744,4781 | .0010 | PS(31:0) | [M+Na]+ | 1,06±0,11 | 1,10±0,10 | 0,2528 |
| 746,5326 | .0004 | PS(P-34:2) | [M+H]+ | 1,21±0,10 | 1,36±0,21 | 0,0881 |
| 748,4916 | .0207 | PS(33:1) | [M+H]+ | 0,93±0,06 | 0,95±0,07 | 0,4355 |
| 754,4757 | .0104 | PS(34:5) | [M+H]+ | 0,87±0,08 | 0,91±0,06 | 0,1691 |
| 756,4907 | .0097 | PS(34:4) | [M+H]+ | 0,86±0,07 | 0,90±0,06 | 0,0734 |
| 758,4903 | .0044 | PS(32:0) | [M+Na]+ | 0,93±0,08 | 0,98±0,06 | 0,0713 |
| 760,4986 | .0136 | PS(34:2) | [M+H]+ | 1,02±0,11 | 1,07±0,09 | 0,1764 |
| 762,4961 | .0318 | PS(34:1) | [M+H]+ | 0,93±0,08 | 0,97±0,07 | 0,1491 |
| 764,4735 | .0257 | PS(33:4) | [M+Na]+ | 0,97±0,05 | 1,03±0,07 | 0,0955 |
| 766,4917 | .0081 | PS(P-34:3) | [M+Na]+ | 0,90±0,07 | 0,94±0,07 | 0,1241 |
| 768,5083 | .0072 | PS(O-34:2) | [M+Na]+ | 0,86±0,06 | 0,88±0,06 | 0,4536 |
| 770,5247 | .0064 | PS(P-34:1) | [M+Na]+ | 0,88±0,07 | 0,94±0,07 | 0,0584 |
| 772,5092 | .0012 | PS(33:0) | [M+Na]+ | 0,92±0,07 | 0,97±0,07 | 0,0867 |
| 774,4962 | .0276 | PS(32:0) | [M+K]+ | 0,87±0,07 | 0,91±0,07 | 0,1609 |
| 776,5119 | .0322 | PS(35:1) | [M+H]+ | 0,88±0,07 | 0,91±0,06 | 0,3186 |
| 778,5156 | .0436 | PS(35:0) | [M+H]+ | 0,89±0,06 | 0,91±0,06 | 0,3902 |
| 780,5211 | .0401 | PS(36:6) | [M+H]+ | 0,88±0,08 | 0,92±0,08 | 0,2646 |
| 782,5552 | .0124 | PS(P-36:0) | [M+Na]+ | 0,90±0,08 | 0,94±0,10 | 0,3207 |
| 784,5193 | .0071 | PS(36:4) | [M+H]+ | 1,11±0,11 | 1,16±0,09 | 0,2011 |
| 786,5172 | .0089 | PS(34:0) | [M+Na]+ | 1,17±0,13 | 1,24±0,11 | 0,1976 |
| 788,5260 | .0175 | PS(36:2) | [M+H]+ | 0,90±0,07 | 0,96±0,07 | 0,2273 |
| 790,5677 | .0085 | PS(36:1) | [M+H]+ | 1,14±0,10 | 1,28±0,20 | 0,0596 |
| 792,5243 | .0069 | PS(P-38:7) | [M+H]+ | 0,88±0,06 | 0,93±0,06 | 0,0786 |
| 794,5202 | .0236 | PS(37:6) | [M+H]+ | 0,94±0,07 | 0,98±0,08 | 0,2787 |
| 796,5673 | .0186 | PS(P-38:5) | [M+H]+ | 0,83±0,08 | 0,85±0,07 | 0,5645 |
| 798,5109 | .0151 | PS(35:1) | [M+Na]+ | 0,86±0,06 | 0,88±0,07 | 0,3361 |
| 800,5179 | .0029 | PS(O-35:1) | [M+K]+ | 0,91±0,08 | 0,94±0,08 | 0,4646 |
| 804,5426 | .0323 | PS(37:1) | [M+H]+ | 0,90±0,09 | 0,92±0,06 | 0,5633 |
| 806,5329 | .0362 | PS(38:7) | [M+H]+ | 0,83±0,06 | 0,87±0,06 | 0,1921 |
| 808,5256 | .0133 | PS(38:6) | [M+H]+ | 0,82±0,05 | 0,86±0,07 | 0,1625 |
| 810,5295 | .0015 | PS(38:5) | [M+H]+ | 0,82±0,07 | 0,88±0,07 | 0,0581 |
| 812,5353 | .0064 | PS(36:1) | [M+Na]+ | 0,81±0,06 | 0,84±0,06 | 0,3032 |
| 814,5264 | .0100 | PS(P-36:0) | [M+K]+ | 0,85±0,08 | 0,85±0,07 | 0,6721 |
| 816,5392 | .0236 | PS(35:0) | [M+K]+ | 0,82±0,07 | 0,87±0,06 | 0,0566 |
| 818,5385 | .0074 | PS(O-38:5) | [M+Na]+ | 0,81±0,07 | 0,83±0,06 | 0,4719 |
| 820,5418 | .0050 | PS(P-38:4) | [M+Na]+ | 0,79±0,06 | 0,83±0,07 | 0,0589 |
| 822,5269 | .0008 | PS(37:3) | [M+Na]+ | 0,78±0,07 | 0,81±0,05 | 0,1143 |
| 824,5725 | .0056 | PS(P-38:2) | [M+Na]+ | 0,84±0,07 | 0,84±0,06 | 0,8038 |
| 826,5310 | .0053 | PS(O-37:2) | [M+K]+ | 0,79±0,07 | 0,83±0,06 | 0,1443 |
| 828,5500 | .0020 | PS(P-37:1) | [M+K]+ | 0,96±0,11 | 1,01±0,10 | 0,2359 |
| 830,5472 | .0159 | PS(36:0) | [M+K]+ | 0,81±0,08 | 0,84±0,06 | 0,2607 |
| 832,5711 | .0350 | PS(39:1) | [M+H]+ | 0,81±0,07 | 0,85±0,06 | 0,1643 |
| 834,5975 | .0243 | PS(39:0) | [M+H]+ | 1,05±0,11 | 1,18±0,21 | 0,0611 |
| 836,5447 | .0012 | PS(40:6) | [M+H]+ | 0,78±0,06 | 0,82±0,07 | 0,1367 |
| 838,5387 | .0186 | PS(38:2) | [M+Na]+ | 0,85±0,06 | 0,90±0,08 | 0,0869 |
| 840,5753 | .0005 | PS(40:4) | [M+H]+ | 0,75±0,07 | 0,80±0,07 | 0,0911 |
| 842,5483 | .0170 | PS(37:1) | [M+K]+ | 0,78±0,06 | 0,81±0,06 | 0,2722 |
| 844,5028 | .0033 | PS(37:0) | [M+K]+ | 0,77±0,06 | 0,80±0,06 | 0,2912 |
| 846,5593 | .0031 | PS(P-40:5) | [M+Na]+ | 0,80±0,06 | 0,83±0,06 | 0,2899 |
| 848,5602 | .0167 | PS(41:7) | [M+H]+ | 0,79±0,06 | 0,82±0,06 | 0,1763 |
| 850,5530 | .0043 | PS(39:3) | [M+Na]+ | 0,75±0,05 | 0,81±0,06 | 0,0665 |
| 852,4685 | .0262 | PS(39:2) | [M+Na]+ | 0,75±0,07 | 0,77±0,06 | 0,3051 |
| 854,5535 | .0222 | PS(38:2) | [M+K]+ | 0,76±0,06 | 0,80±0,07 | 0,1185 |
| 856,5650 | .0181 | PS(38:1) | [M+K]+ | 0,75±0,06 | 0,79±0,07 | 0,1313 |
| 858,5693 | .0067 | PS(38:0) | [M+K]+ | 0,76±0,07 | 0,79±0,07 | 0,2608 |
| 862,5628 | .0036 | PS(42:7) | [M+H]+ | 0,73±0,05 | 0,76±0,06 | 0,2396 |
| 864,5612 | .0118 | PS(40:3) | [M+Na]+ | 0,76±0,06 | 0,78±0,06 | 0,3683 |
| 866,5554 | .0123 | PS(O-40:3) | [M+K]+ | 0,72±0,06 | 0,75±0,06 | 0,3063 |
| 868,5518 | .0049 | PS(39:2) | [M+K]+ | 0,73±0,07 | 0,77±0,06 | 0,0799 |
| 870,5620 | .0006 | PS(39:1) | [M+K]+ | 0,73±0,07 | 0,75±0,06 | 0,4043 |
| 872,5785 | .0002 | PS(39:0) | [M+K]+ | 0,88±0,10 | 0,92±0,08 | 0,1799 |
| 874,5730 | .0137 | PS(43:8) | [M+H]+ | 0,77±0,07 | 0,81±0,06 | 0,1562 |
| 876,5991 | .0103 | PS(O-42:4) | [M+Na]+ | 0,79±0,05 | 0,83±0,07 | 0,1006 |
| 878,6289 | .0020 | PS(P-44:6) | [M+H]+ | 0,98±0,08 | 1,12±0,20 | 0,0555 |
| 880,5694 | .0079 | PS(41:2) | [M+Na]+ | 0,82±0,08 | 0,86±0,11 | 0,3374 |
| 882,6080 | .0119 | PS(41:1) | [M+Na]+ | 0,88±0,09 | 0,91±0,11 | 0,4768 |
| 884,6306 | .0050 | PS(41:0) | [M+Na]+ | 0,77±0,07 | 0,76±0,07 | 0,8662 |
| 886,6143 | .0204 | PS(40:0) | [M+K]+ | 0,78±0,10 | 0,79±0,09 | 0,7368 |
| 888,5757 | .0008 | PS(44:8) | [M+H]+ | 0,72±0,06 | 0,76±0,06 | 0,0956 |
| 890,5904 | .0002 | PS(44:7) | [M+H]+ | 0,73±0,07 | 0,77±0,06 | 0,1383 |
| 892,5901 | .0142 | PS(42:3) | [M+Na]+ | 0,73±0,06 | 0,77±0,05 | 0,0615 |
| 894,5757 | .0131 | PS(41:3) | [M+K]+ | 0,73±0,06 | 0,77±0,06 | 0,0762 |
| 896,5707 | .0075 | PS(41:2) | [M+K]+ | 0,72±0,06 | 0,76±0,06 | 0,1455 |
| 898,5970 | .0032 | PS(41:1) | [M+K]+ | 0,77±0,06 | 0,81±0,07 | 0,0907 |
| 900,5771 | .0041 | PS(43:6) | [M+Na]+ | 0,72±0,07 | 0,72±0,06 | 0,8900 |
| 902,5873 | .0014 | PS(43:5) | [M+Na]+ | 0,72±0,06 | 0,75±0,06 | 0,1817 |
| 904,6312 | .0095 | PS(O-44:4) | [M+Na]+ | 0,85±0,10 | 0,89±0,10 | 0,3283 |
| 906,6178 | .0021 | PS(43:3) | [M+Na]+ | 0,78±0,07 | 0,79±0,08 | 0,6739 |
| 908,6518 | .0144 | PS(45:5) | [M+H]+ | 0,99±0,15 | 1,03±0,22 | 0,6213 |
| 910,6787 | .0089 | PS(O-44:1) | [M+Na]+ | 1,11±0,24 | 1,16±0,29 | 0,6077 |
| 912,6435 | .0234 | PS(43:0) | [M+Na]+ | 0,77±0,09 | 0,79±0,08 | 0,4927 |
| 914,5980 | .0075 | PS(46:9) | [M+H]+ | 0,72±0,08 | 0,74±0,06 | 0,6237 |
| 916,6102 | .0040 | PS(46:8) | [M+H]+ | 0,80±0,08 | 0,82±0,07 | 0,3980 |
| 918,6180 | .0019 | PS(44:4) | [M+Na]+ | 0,73±0,07 | 0,77±0,06 | 0,1814 |
| 920,6396 | .0021 | PS(46:6) | [M+H]+ | 0,75±0,07 | 0,79±0,06 | 0,1212 |
| 922,6638 | .0107 | PS(46:5) | [M+H]+ | 0,94±0,11 | 1,09±0,20 | 0,0648 |
| 924,6087 | .0009 | PS(43:2) | [M+K]+ | 0,75±0,07 | 0,77±0,06 | 0,4236 |
| 926,6114 | .0228 | PS(45:7) | [M+Na]+ | 0,82±0,08 | 0,88±0,09 | 0,1459 |
| 928,6086 | .0042 | PS(45:6) | [M+Na]+ | 0,71±0,06 | 0,73±0,06 | 0,6512 |
| 930,6330 | .0112 | PS(47:8) | [M+H]+ | 0,79±0,09 | 0,82±0,10 | 0,4939 |
| 934,6661 | .0130 | PS(47:6) | [M+H]+ | 0,91±0,14 | 0,94±0,15 | 0,6155 |
| 936,6212 | .0116 | PS(44:3) | [M+K]+ | 0,71±0,08 | 0,73±0,06 | 0,5509 |
| 938,6091 | .0160 | PS(44:2) | [M+K]+ | 0,70±0,06 | 0,72±0,05 | 0,2588 |
| 940,6253 | .0155 | PS(44:1) | [M+K]+ | 0,70±0,07 | 0,73±0,06 | 0,2739 |
| 942,6170 | .0029 | PS(46:6) | [M+Na]+ | 0,72±0,08 | 0,75±0,06 | 0,2525 |
| 946,6218 | .0279 | PS(45:5) | [M+K]+ | 0,69±0,06 | 0,73±0,06 | 0,1664 |
| 948,6514 | .0155 | PS(46:3) | [M+Na]+ | 0,75±0,08 | 0,81±0,10 | 0,1732 |
| 950,6360 | .0108 | PS(45:3) | [M+K]+ | 0,70±0,07 | 0,72±0,07 | 0,4735 |
| 952,6209 | .0166 | PS(47:8) | [M+Na]+ | 0,69±0,06 | 0,70±0,06 | 0,7121 |
| 954,6102 | .0097 | PS(47:7) | [M+Na]+ | 0,66±0,06 | 0,69±0,06 | 0,2195 |
| 956,6349 | .0007 | PS(47:6) | [M+Na]+ | 0,69±0,07 | 0,72±0,07 | 0,2796 |
| 958,6434 | .0078 | PS(47:5) | [M+Na]+ | 0,67±0,07 | 0,70±0,07 | 0,2313 |
| 960,6546 | .0123 | PS(47:4) | [M+Na]+ | 0,71±0,07 | 0,74±0,07 | 0,1543 |
| 962,6273 | .0021 | PS(46:4) | [M+K]+ | 0,67±0,06 | 0,69±0,06 | 0,5691 |
| 964,6498 | .0090 | PS(46:3) | [M+K]+ | 0,70±0,07 | 0,73±0,06 | 0,1635 |
| 966,6946 | .0192 | PS(47:1) | [M+Na]+ | 0,91±0,12 | 1,04±0,20 | 0,0633 |
| 968,6372 | .0002 | PS(50:10) | [M+H]+ | 0,65±0,06 | 0,68±0,06 | 0,2373 |
| 970,6309 | .0203 | PS(48:6) | [M+Na]+ | 0,68±0,05 | 0,72±0,07 | 0,1643 |
| 972,6336 | .0240 | PS(47:6) | [M+K]+ | 0,63±0,06 | 0,65±0,06 | 0,5195 |
| 974,6313 | .0061 | PS(47:5) | [M+K]+ | 0,65±0,07 | 0,67±0,06 | 0,4349 |
| 976,6396 | .0013 | PS(47:4) | [M+K]+ | 0,65±0,06 | 0,64±0,06 | 0,9244 |
| 978,6340 | .0141 | PS(49:9) | [M+Na]+ | 0,64±0,05 | 0,66±0,05 | 0,3258 |
| 980,6324 | .0033 | PS(49:8) | [M+Na]+ | 0,64±0,05 | 0,65±0,04 | 0,3739 |
| 982,6500 | .0012 | PS(49:7) | [M+Na]+ | 0,64±0,06 | 0,67±0,06 | 0,2554 |
| 984,6260 | .0164 | PS(48:7) | [M+K]+ | 0,63±0,05 | 0,64±0,05 | 0,5328 |
| 986,6332 | .0080 | PS(48:6) | [M+K]+ | 0,64±0,06 | 0,66±0,05 | 0,0663 |
| 988,6296 | .0113 | PS(48:5) | [M+K]+ | 0,61±0,05 | 0,63±0,05 | 0,2687 |
| 990,6420 | .0144 | PS(48:4) | [M+K]+ | 0,61±0,05 | 0,63±0,05 | 0,2424 |
| 992,6448 | .0074 | PS(52:12) | [M+H]+ | 0,60±0,06 | 0,63±0,05 | 0,2417 |
| 994,6466 | .0046 | PS(50:8) | [M+Na]+ | 0,59±0,05 | 0,61±0,06 | 0,3206 |
| 998,6278 | .0027 | PS(49:7) | [M+K]+ | 0,60±0,06 | 0,62±0,05 | 0,5080 |
| 566,3838 | .0021 | PC(20:0) | [M+H]+ | 1,40±0,70 | 1,56±0,00 | 0,5384 |
| 594,4299 | .0170 | PC(22:0) | [M+H]+ | 2,21±0,68 | 2,42±0,79 | 0,5939 |
| 658,5254 | .0467 | PC(O-26:0) | [M+Na]+ | 2,66±0,56 | 3,08±0,67 | 0,1156 |
| 692,5683 | .0094 | PC(O-30:0) | [M+H]+ | 2,49±0,37 | 2,49±0,00 | 0,9821 |
| 702,5671 | .0124 | PC(P-32:0) | [M+H]+ | 2,67±0,53 | 2,91±0,56 | 0,2685 |
| 704,5656 | .0068 | PC(O-31:1) | [M+H]+ | 1,96±0,30 | 2,18±0,45 | 0,1381 |
| 706,5832 | .0087 | PC(O-31:0) | [M+H]+ | 2,34±0,31 | 2,44±0,36 | 0,4536 |
| 712,5713 | .0456 | PC(P-30:1) | [M+Na]+ | 2,74±0,55 | 2,94±0,49 | 0,3256 |
| 714,5728 | .0315 | PC(O-30:0) | [M+Na]+ | 2,35±0,45 | 2,59±0,40 | 0,1162 |
| 716,5721 | .0133 | PC(P-32:2) | [M+H]+ | 2,40±0,48 | 2,58±0,57 | 0,3801 |
| 718,5741 | .0359 | PC(31:1) | [M+H]+ | 2,09±0,25 | 2,24±0,26 | 0,1378 |
| 720,5937 | .0036 | PC(O-32:0) | [M+H]+ | 3,33±0,50 | 3,24±0,45 | 0,6506 |
| 728,5894 | .0306 | PC(P-33:2) | [M+H]+ | 2,84±0,48 | 2,99±0,44 | 0,3903 |
| 730,5984 | .0239 | PC(P-33:2) | [M+H]+ | 2,63±0,39 | 2,71±0,28 | 0,5286 |
| 732,6224 | .0323 | PC(O-33:1) | [M+H]+ | 3,10±0,48 | 3,00±0,48 | 0,5943 |
| 734,6291 | .0234 | PC(O-33:0) | [M+H]+ | 2,69±0,30 | 2,58±0,34 | 0,4409 |
| 740,5939 | .0351 | PC(O-34:4) | [M+H]+ | 2,86±0,44 | 2,98±0,44 | 0,5037 |
| 742,5973 | .0229 | PC(P-34:3) | [M+H]+ | 2,42±0,33 | 2,53±0,29 | 0,3592 |
| 744,5954 | .0053 | PC(P-34:2) | [M+H]+ | 3,31±0,59 | 3,45±0,49 | 0,5157 |
| 746,6164 | .0106 | PC(P-34:1) | [M+H]+ | 6,65±1,65 | 6,80±1,47 | 0,7957 |
| 748,6341 | .0126 | PC(O-34:0) | [M+H]+ | 6,14±1,03 | 5,79±1,12 | 0,4489 |
| 759,5621 | .0026 | PC(33:3) | [M+NH4]+ | 8,82±2,52 | 7,43±2,21 | *0,0300** |
| 762,6729 | .0358 | PC(O-35:0) | [M+H]+ | 3,79±0,54 | 3,32±0,59 | 0,0607 |
| 770,6264 | .0206 | PC(P-36:3) | [M+H]+ | 4,62±0,85 | 4,56±0,74 | 0,8562 |
| 774,6708 | .0338 | PC(P-36:1) | [M+H]+ | 6,56±1,16 | 6,34±1,27 | 0,6495 |
| 782,5624 | .0071 | PC(36:4) | [M+H]+ | 16,42±2,06 | 14,94±2,29 | *0,0210** |
| 783,5658 | .0011 | PC(35:5) | [M+NH4]+ | 10.91 ± 2.41 | 9.82 ± 2.33 | *0,0191** |
| 784,5800 | .0033 | PC(34:0) | [M+Na]+ | 9.35 ± 2.94 | 8.01 ± 2.63 | *0,0221** |
| 790,6931 | .0248 | PC(O-37:0) | [M+H]+ | 4,73±0,65 | 4,49±0,63 | 0,3810 |
| 802,6769 | .0085 | PC(P-38:1) | [M+H]+ | 4,28±0,72 | 4,32±0,73 | 0,8855 |
| 804,7010 | .0170 | PC(O-38:0) | [M+H]+ | 11,83±2,66 | 11,91±2,51 | 0,9365 |
| 807,5658 | .0011 | PC(37:7) | [M+NH4]+ | 8.52 ± 1.91 | 7.94 ± 1.92 | *0.0742** |
| 809,5847 | .0044 | PC(37:6) | [M+NH4]+ | 10.81 ± 2.42 | 9.83 ± 2.33 | *0.0303** |
| 810,5651 | .0338 | PC(36:1) | [M+Na]+ | 10.01 ± 2.00 | 9.12 ± 2.22 | *0.0372** |
| 814,7044 | .0003 | PC(P-40:0) | [M+H]+ | 4,10±0,59 | 3,86±0,65 | 0,3879 |
| 816,7152 | .0311 | PC(P-39:0) | [M+H]+ | 4,61±0,72 | 4,28±0,72 | 0,2789 |
| 818,7192 | .0196 | PC(O-39:0) | [M+H]+ | 4,28±0,71 | 4,09±0,68 | 0,5051 |
| 826,7044 | .0360 | PC(P-40:3) | [M+H]+ | 3,26±0,41 | 3,20±0,50 | 0,7632 |
| 828,7015 | .0175 | PC(O-40:2) | [M+H]+ | 6,89±1,66 | 6,91±1,47 | 0,9874 |
| 830,7178 | .0181 | PC(P-40:1) | [M+H]+ | 6,83±1,38 | 6,94±1,32 | 0,8314 |
| 832,7362 | .0208 | PC(O-40:0) | [M+H]+ | 9,70±2,12 | 9,88±2,17 | 0,8358 |
| 836,7358 | .0485 | PC(P-40:0) | [M+Na]+ | 4,67±0,63 | 4,38±0,75 | 0,3596 |
| 838,7002 | .0337 | PC(P-39:1) | [M+Na]+ | 3,78±0,80 | 3,68±0,74 | 0,7249 |
| 840,7158 | .0337 | PC(O-39:0) | [M+Na]+ | 3,84±0,56 | 3,77±0,67 | 0,7956 |
| 842,7158 | .0162 | PC(P-41:1) | [M+H]+ | 3,33±0,46 | 3,27±0,51 | 0,7799 |
| 844,7081 | .0292 | PC(40:1) | [M+H]+ | 3,09±0,45 | 3,11±0,44 | 0,9221 |
| 846,6936 | .0010 | PC(40:0) | [M+H]+ | 3,22±0,44 | 3,31±0,50 | 0,6545 |
| 850,6899 | .0215 | PC(P-42:5) | [M+H]+ | 3,13±0,43 | 3,20±0,42 | 0,7227 |
| 852,6853 | .0376 | PC(41:4) | [M+H]+ | 3,52±0,68 | 3,71±0,81 | 0,5071 |
| 854,6993 | .0003 | PC(O-42:3) | [M+H]+ | 3,59±0,60 | 3,63±0,67 | 0,8734 |
| 856,7244 | .0091 | PC(O-42:2) | [M+H]+ | 4,63±1,05 | 4,71±0,85 | 0,8271 |
| 858,7396 | .0030 | PC(O-42:1) | [M+H]+ | 3,82±0,75 | 3,86±0,62 | 0,8678 |
| 860,7203 | .0101 | PC(41:0) | [M+H]+ | 2,91±0,44 | 2,91±0,42 | 0,9837 |
| 864,6949 | .0128 | PC(P-41:1) | [M+Na]+ | 3,00±0,58 | 3,04±0,50 | 0,8509 |
| 866,7029 | .0033 | PC(P-43:3) | [M+H]+ | 2,75±0,55 | 2,82±0,49 | 0,7476 |
| 868,6887 | .0098 | PC(42:3) | [M+H]+ | 2,85±0,62 | 3,04±0,65 | 0,4257 |
| 870,6959 | .0012 | PC(42:2) | [M+H]+ | 2,51±0,45 | 2,60±0,44 | 0,6153 |
| 872,7029 | .0074 | PC(42:1) | [M+H]+ | 2,32±0,32 | 2,42±0,31 | 0,5027 |
| 874,7040 | .0219 | PC(42:0) | [M+H]+ | 2,34±0,32 | 2,43±0,33 | 0,5730 |
| 876,6902 | .0062 | PC(O-44:6) | [M+H]+ | 2,45±0,43 | 2,52±0,37 | 0,6244 |
| 880,7061 | .0074 | PC(P-42:1) | [M+Na]+ | 2,70±0,62 | 2,89±0,61 | 0,3920 |
| 882,7090 | .0144 | PC(43:3) | [M+H]+ | 2,43±0,56 | 2,56±0,48 | 0,5149 |
| 884,6972 | .0130 | PC(43:2) | [M+H]+ | 2,05±0,39 | 2,13±0,35 | 0,5531 |
| 886,6934 | .0325 | PC(43:1) | [M+H]+ | 1,82±0,25 | 1,92±0,26 | 0,2866 |
| 888,7010 | .0405 | PC(43:0) | [M+H]+ | 1,80±0,27 | 1,87±0,25 | 0,4750 |
| 890,6928 | .0295 | PC(44:6) | [M+H]+ | 2,10±0,37 | 2,13±0,35 | 0,8500 |
| 892,7052 | .0263 | PC(44:5) | [M+H]+ | 3,01±0,76 | 2,83±0,69 | 0,5062 |
| 894,7148 | .0202 | PC(44:4) | [M+H]+ | 2,65±0,62 | 2,61±0,59 | 0,8605 |
| 896,7121 | .0019 | PC(44:3) | [M+H]+ | 2,29±0,52 | 2,45±0,49 | 0,3848 |
| 898,7036 | .0223 | PC(44:2) | [M+H]+ | 1,93±0,32 | 1,98±0,29 | 0,6323 |
| 900,7012 | .0190 | PC(O-44:5) | [M+Na]+ | 1,75±0,24 | 1,80±0,22 | 0,6200 |
| 902,6965 | .0013 | PC(P-44:4) | [M+Na]+ | 1,67±0,27 | 1,72±0,24 | 0,6667 |
| 904,6990 | .0145 | PC(O-44:3) | [M+Na]+ | 1,85±0,31 | 1,94±0,27 | 0,4149 |
| 906,7048 | .0102 | PC(45:5) | [M+H]+ | 1,81±0,35 | 1,90±0,32 | 0,4919 |
| 908,7034 | .0050 | PC(43:1) | [M+Na]+ | 2,26±0,50 | 2,20±0,53 | 0,7525 |
| 910,7023 | .0008 | PC(O-43:1) | [M+K]+ | 2,02±0,36 | 2,12±0,42 | 0,5519 |
| 912,7090 | .0097 | PC(O-43:0) | [M+K]+ | 1,74±0,27 | 1,89±0,31 | 0,2563 |
| 914,6999 | .0365 | PC(46:8) | [M+H]+ | 2,57±0,40 | 2,44±0,45 | 0,4361 |
| 916,7152 | .0363 | PC(46:7) | [M+H]+ | 2,84±0,54 | 2,66±0,50 | 0,4041 |
| 918,7228 | .0063 | PC(P-45:3) | [M+Na]+ | 2,46±0,51 | 2,28±0,47 | 0,3547 |
| 920,7218 | .0116 | PC(46:5) | [M+H]+ | 2,30±0,52 | 2,19±0,45 | 0,5369 |
| 922,7155 | .0085 | PC(44:1) | [M+Na]+ | 1,98±0,23 | 2,12±0,35 | 0,3648 |
| 924,7053 | .0229 | PC(43:1) | [M+K]+ | 1,78±0,28 | 1,87±0,27 | 0,3883 |
| 926,7045 | .0065 | PC(43:0) | [M+K]+ | 1,62±0,20 | 1,68±0,19 | 0,4701 |
| 928,7097 | .0037 | PC(P-46:5) | [M+Na]+ | 1,52±0,19 | 1,61±0,19 | 0,2215 |
| 930,7296 | .0005 | PC(P-46:4) | [M+Na]+ | 2,06±0,43 | 2,02±0,36 | 0,7593 |
| 932,7299 | .0149 | PC(O-46:3) | [M+Na]+ | 1,84±0,35 | 1,82±0,28 | 0,8210 |
| 934,7113 | .0127 | PC(45:2) | [M+Na]+ | 1,67±0,28 | 1,75±0,30 | 0,4993 |
| 936,7173 | .0014 | PC(P-45:2) | [M+K]+ | 1,77±0,33 | 1,84±0,38 | 0,6988 |
| 938,7187 | .0207 | PC(44:1) | [M+K]+ | 2,21±0,41 | 2,31±0,39 | 0,5023 |
| 940,7263 | .0126 | PC(44:0) | [M+K]+ | 2,33±0,43 | 2,26±0,40 | 0,6532 |
| 946,7325 | .0066 | PC(48:6) | [M+H]+ | 1,94±0,41 | 1,87±0,37 | 0,6507 |
| 948,7214 | .0183 | PC(46:2) | [M+Na]+ | 1,77±0,33 | 1,74±0,30 | 0,8156 |
| 950,7456 | .0097 | PC(46:1) | [M+Na]+ | 2,13±0,56 | 1,93±0,50 | 0,3203 |
| 952,7500 | .0000 | PC(P-46:1) | [M+K]+ | 1,83±0,37 | 1,73±0,36 | 0,4812 |
| 954,7480 | .0187 | PC(45:0) | [M+K]+ | 1,53±0,19 | 1,58±0,19 | 0,6518 |
| 956,7422 | .0025 | PC(O-48:5) | [M+Na]+ | 1,46±0,19 | 1,47±0,19 | 0,9305 |
| 958,7543 | .0061 | PC(P-48:4) | [M+Na]+ | 1,74±0,27 | 1,69±0,23 | 0,6906 |
| 960,7417 | .0002 | PC(49:6) | [M+H]+ | 1,50±0,25 | 1,50±0,20 | 0,9982 |
| 962,7272 | .0281 | PC(47:2) | [M+Na]+ | 1,68±0,28 | 1,77±0,25 | 0,3817 |
| 964,7385 | .0115 | PC(O-47:2) | [M+K]+ | 1,82±0,31 | 1,86±0,30 | 0,7465 |
| 966,7544 | .0251 | PC(46:1) | [M+K]+ | 2,21±0,42 | 2,39±0,49 | 0,3367 |
| 968,7607 | .0157 | PC(46:0) | [M+K]+ | 1,87±0,31 | 1,83±0,31 | 0,7867 |
| 970,7470 | .0211 | PC(50:8) | [M+H]+ | 1,58±0,28 | 1,58±0,22 | 0,9318 |
| 972,7795 | .0380 | PC(50:7) | [M+H]+ | 2,70±0,53 | 2,52±0,57 | 0,4147 |
| 974,7987 | .0070 | PC(P-49:3) | [M+Na]+ | 3,01±0,64 | 2,76±0,74 | 0,3534 |
| 976,8004 | .0275 | PC(50:5) | [M+H]+ | 2,30±0,58 | 2,13±0,56 | 0,4371 |
| 978,7874 | .0008 | PC(48:1) | [M+Na]+ | 1,79±0,44 | 1,64±0,40 | 0,3536 |
| 980,7673 | .0223 | PC(47:1) | [M+K]+ | 1,48±0,22 | 1,46±0,22 | 0,8865 |
| 982,7747 | .0142 | PC(47:0) | [M+K]+ | 1,47±0,20 | 1,48±0,21 | 0,8642 |
| 984,7681 | .0266 | PC(51:8) | [M+H]+ | 1,30±0,15 | 1,32±0,15 | 0,7667 |
| 986,7673 | .0101 | PC(51:7) | [M+H]+ | 1,40±0,20 | 1,41±0,23 | 0,9042 |
| 988,7767 | .0039 | PC(51:6) | [M+H]+ | 1,96±0,47 | 1,91±0,46 | 0,7460 |
| 990,7660 | .0206 | PC(49:2) | [M+Na]+ | 1,72±0,32 | 1,68±0,30 | 0,7190 |
| 992,7668 | .0219 | PC(48:2) | [M+K]+ | 1,51±0,28 | 1,52±0,26 | 0,8763 |
| 994,7769 | .0163 | PC(48:1) | [M+K]+ | 1,48±0,29 | 1,48±0,28 | 0,9445 |
| 996,7868 | .0105 | PC(48:0) | [M+K]+ | 2,06±0,45 | 2,17±0,48 | 0,5311 |
| 998,8083 | .0428 | PC(51:1) | [M+H]+ | 2,38±0,53 | 2,33±0,56 | 0,8058 |
| 412,2353 | .0105 | LPC(10:0) | [M+H]+ | 0,86±0,20 | 0,96±0,21 | 0,3356 |
| 434,2549 | .0266 | LPC(10:0) | [M+Na]+ | 1,14±0,49 | 1,13±0,36 | 0,9291 |
| 440,2492 | .0279 | LPC(12:0) | [M+H]+ | 0,92±0,16 | 0,96±0,16 | 0,5562 |
| 450,2144 | .0121 | LPC(10:0) | [M+K]+ | 1,16±0,44 | 1,32±0,37 | 0,2723 |
| 466,3160 | .0232 | LPC(14:1) | [M+H]+ | 1,91±0,46 | 1,97±0,58 | 0,7439 |
| 468,2961 | .0123 | LPC(14:0) | [M+H]+ | 1,11±0,29 | 1,13±0,21 | 0,8289 |
| 480,3637 | .0189 | LPC(P-16:0) | [M+H]+ | 1,04±0,25 | 0,98±0,17 | 0,4252 |
| 482,3487 | .0246 | LPC(15:0) | [M+H]+ | 1,69±0,33 | 1,51±0,26 | 0,0728 |
| 494,3412 | .0172 | LPC(16:1) | [M+H]+ | 1,01±0,25 | 0,97±0,19 | 0,6873 |
| 508,3521 | .0124 | LPC(17:1) | [M+H]+ | 1,92±0,58 | 1,95±0,67 | 0,8806 |
| 510,4167 | .0250 | LPC(O-18:0) | [M+H]+ | 2,16±0,58 | 1,92±0,46 | 0,1894 |
| 532,4197 | .0455 | LPC(O-18:0) | [M+Na]+ | 1,80±0,43 | 1,71±0,35 | 0,4829 |
| 536,4237 | .0163 | LPC(P-20:0) | [M+H]+ | 1,66±0,33 | 1,54±0,29 | 0,2685 |
| 538,4474 | .0244 | LPC(O-20:0) | [M+H]+ | 1,61±0,34 | 1,39±0,24 | 0,0566 |
| 550,3880 | .0014 | LPC(20:1) | [M+H]+ | 1,30±0,21 | 1,31±0,21 | 0,9509 |
| 552,4008 | .0015 | LPC(20:0) | [M+H]+ | 1,15±0,21 | 1,25±0,20 | 0,3000 |
| 558,3985 | .0086 | LPC(P-20:0) | [M+Na]+ | 1,79±0,40 | 1,83±0,40 | 0,8211 |
| 560,4074 | .0018 | LPC(O-20:0) | [M+Na]+ | 1,29±0,31 | 1,41±0,32 | 0,3587 |
| 564,3437 | .0371 | LPC(20:5) | [M+Na]+ | 1,78±0,71 | 1,64±0,46 | 0,5335 |
| 566,4002 | .0177 | LPC(21:0) | [M+H]+ | 2,34±0,65 | 2,32±0,74 | 0,9647 |
| 574,4144 | .0296 | LPC(20:0) | [M+Na]+ | 1,21±0,32 | 1,21±0,20 | 0,9693 |
| 588,4249 | .0245 | LPC(21:0) | [M+Na]+ | 0,99±0,28 | 1,07±0,19 | 0,4227 |
| 594,3623 | .0088 | LPC(22:4) | [M+Na]+ | 1,39±0,47 | 1,51±0,30 | 0,3419 |
| 602,4210 | .0031 | LPC(24:3) | [M+H]+ | 1,23±0,30 | 1,34±0,27 | 0,3059 |
| 616,4761 | .0444 | LPC(23:0) | [M+Na]+ | 1,69±0,58 | 2,10±0,56 | 0,0507 |
| 618,4199 | .0299 | LPC(22:0) | [M+K]+ | 0,91±0,26 | 0,99±0,17 | 0,5078 |
| 620,5015 | .0365 | LPC(25:1) | [M+H]+ | 1,21±0,32 | 1,26±0,26 | 0,7161 |
| 630,4619 | .0127 | LPC(26:3) | [M+H]+ | 1,40±0,43 | 1,48±0,34 | 0,5851 |
| 632,4677 | .0027 | LPC(26:2) | [M+H]+ | 1,09±0,25 | 1,22±0,28 | 0,2544 |
| 634,5138 | .0333 | LPC(26:1) | [M+H]+ | 0,93±0,21 | 0,94±0,14 | 0,8556 |
| 658,5169 | .0364 | LPC(28:3) | [M+H]+ | 2,23±0,80 | 2,30±0,58 | 0,7758 |
| 660,5253 | .0291 | LPC(28:2) | [M+H]+ | 1,33±0,33 | 1,54±0,27 | 0,0681 |
| 662,5489 | .0371 | LPC(28:1) | [M+H]+ | 1,27±0,30 | 1,49±0,31 | 0,0766 |
| 670,5211 | .0424 | LPC(27:1) | [M+Na]+ | 2,32±0,39 | 2,39±0,38 | 0,6790 |
| 678,5885 | .0453 | LPC(29:0) | [M+H]+ | 1,66±0,39 | 1,77±0,28 | 0,3937 |
| 686,4523 | .0004 | LPC(27:1) | [M+K]+ | 1.82 ± 0.51 | 2.20 ± 0.51 | *0.0081** |
| 688,4642 | .0041 | LPC(27:0) | [M+K]+ | 2.41 ± 0.90 | 2.91 ± 0.92 | *0.0411** |
| 690,5836 | .0405 | LPC(30:1) | [M+H]+ | 1,66±0,40 | 1,86±0,39 | 0,1749 |
| 706,6022 | .0278 | LPC(31:0) | [M+H]+ | 1,75±0,40 | 1,85±0,34 | 0,4785 |
| 718,5810 | .0065 | LPC(32:1) | [M+H]+ | 1,34±0,24 | 1,49±0,24 | 0,1306 |
| 770,6494 | .0455 | LPC(34:0) | [M+Na]+ | 2,68±0,62 | 3,06±0,74 | 0,1519 |

**Sphingolipids**

| Imput mass | Delta | Abbreviation | Ion | No OSA (n=16) | OSA (n=37) | p value |
| --- | --- | --- | --- | --- | --- | --- |
| 478.3857 | .0378 | Cer(d18:0/10:0) | [M+Na]+ | 3.38±0.60 | 3.22±0.69 | 0.4977 |
| 526.3545 | .0190 | GlcCer(d18:1/2:0) | [M+Na]+ | 1.38±0.08 | 1.47±0.17 | 0.1165 |
| 642.6199 | .0380 | Cer(d18:1/24:4(5Z.8Z.11Z.14Z)) | [M+H]+ | 1.68±0.30 | 1.70±0.34 | 0.9006 |
| 652.6933 | .0331 | Cer(d18:0/24:0) | [M+H]+ | 0.79±0.11 | 0.72±0.11 | 0.0866 |
| 658.4497 | .0081 | CerP(d18:0/16:0) | [M+K]+ | 1.36±0.12 | 1.47±0.20 | 0.1139 |
| 664.5345 | .0299 | Cer(d18:1/24:4(5Z.8Z.11Z.14Z)) | [M+Na]+ | 1.38±0.08 | 1.35±0.11 | 0.5060 |
| 680.5047 | .0337 | Cer(d18:1/24:4(5Z.8Z.11Z.14Z)) | [M+K]+ | 0.91±0.08 | 0.94±0.06 | 0.3555 |
| 684.4639 | .0094 | CerP(d18:1/18:0) | [M+K]+ | 0.96±0.06 | 0.98±0.07 | 0.4447 |
| 686.5269 | .0378 | CerP(d18:0/18:0) | [M+K]+ | 2.34±0.44 | 2.41±0.32 | 0.5818 |
| 698.5126 | .0338 | CerP(d18:0/20:0) | [M+Na]+ | 0.91±0.06 | 0.90±0.07 | 0.7519 |
| 704.5728 | .0225 | CerP(d18:0/22:0) | [M+H]+ | 1.11±0.17 | 1.03±0.16 | 0.2064 |
| 712.4984 | .0062 | CerP(d18:1/20:0) | [M+K]+ | 0.90±0.05 | 0.89±0.06 | 0.6991 |
| 714.4789 | .0415 | CerP(d18:0/20:0) | [M+K]+ | 0.94±0.05 | 0.93±0.09 | 0.9302 |
| 726.5428 | .0349 | CerP(d18:0/22:0) | [M+Na]+ | 0.88±0.07 | 0.88±0.08 | 0.8502 |
| 744.4978 | .0329 | CerP(d18:1/24:4(5Z.8Z.11Z.14Z)) | [M+Na]+ | 0.24±0.05 | 0.27±0.04 | *0.0447** |
| 746.5194 | .0270 | CerP(d18:0/24:4(5Z.8Z.11Z.14Z)) | [M+Na]+ | 1.31±0.12 | 1.39±0.18 | 0.1835 |
| 748.6160 | .0456 | GlcCer(d18:1/18:1(9Z)) | [M+Na]+ | 1.10±0.11 | 1.07±0.13 | 0.5885 |
| 754.6079 | .0012 | CerP(d18:0/24:0) | [M+Na]+ | 1.06±0.12 | 1.04±0.12 | 0.6160 |
| 756.6409 | .0062 | GlcCer(d18:1/20:0) | [M+H]+ | 1.42±0.23 | 1.30±0.24 | 0.2298 |
| 758.6503 | .0001 | GlcCer(d18:0/20:0) | [M+H]+ | 1.01±0.08 | 1.02±0.12 | 0.8128 |
| 762.5652 | .0449 | CerP(d18:0/24:4(5Z.8Z.11Z.14Z)) | [M+K]+ | 0.97±0.08 | 0.94±0.08 | 0.3928 |
| 782.6830 | .0427 | CerP(d18:0/26:0) | [M+Na]+ | 1.07±0.13 | 1.04±0.11 | 0.5886 |
| 784.6981 | .0321 | GlcCer(d18:1/22:0) | [M+H]+ | 0.91±0.11 | 0.88±0.09 | 0.4069 |
| 786.6486 | .0331 | GlcCer(d18:0/22:0) | [M+H]+ | 0.87±0.11 | 0.87±0.09 | 0.9076 |
| 796.6563 | .0495 | GlcCer(d18:0/20:0) | [M+K]+ | 0.90±0.12 | 0.88±0.14 | 0.6067 |
| 810.7271 | .0454 | GlcCer(d18:1/24:1(15Z)) | [M+H]+ | 1.03±0.15 | 0.97±0.14 | 0.2339 |
| 814.6811 | .0320 | GlcCer(d18:0/24:0) | [M+H]+ | 0.91±0.14 | 0.84±0.14 | 0.1765 |
| 828.6408 | .0078 | GlcCer(d18:0/24:4(5Z.8Z.11Z.14Z)) | [M+Na]+ | 0.82±0.10 | 0.76±0.12 | 0.1557 |
| 838.7596 | .0466 | GlcCer(d18:1/26:1(17Z)) | [M+H]+ | 1.10±0.21 | 1.02±0.20 | 0.3085 |
| 840.7114 | .0172 | GlcCer(d18:1/26:0) | [M+H]+ | 0.97±0.13 | 0.88±0.15 | 0.1221 |

**Supplemental table 4**

|  | AHI | Minimum O_2_ saturation | TST O_2_ sat <90% |
| --- | --- | --- | --- |
| AHI | 1* |  |  |
| Minimum O_2_ saturation | -0.741* | 1* |  |
| TST O_2_ sat <90% | 0.782* | -0.861* | 1* |
| DG(45:8) | -0.269 | 0.257 | -0.308* |
| Cer(d18:1/16:0) | 0.266 | -0.119 | 0.152 |
| PA(35:2) | -0.384* | 0.392* | -0.395* |
| PE(36:5) | 0.254 | -0.17 | 0.129 |
| PE(35:1) | 0.279* | -0.153 | 0.11 |
| PE(38:6) | 0.242 | -0.138 | 0.095 |
| PE(38:5) | 0.258 | -0.15 | 0.133 |
| PE(37:3) | 0.259 | -0.147 | 0.099 |
| PE(37:2) | 0.252 | -0.171 | 0.13 |
| PE(39:4) | 0.254 | -0.144 | 0.088 |
| PC(33:3) | -0.211 | 0.114 | -0.066 |
| PC(36:4) | -0.246 | 0.19 | -0.227 |
| PC(35:5) | -0.239 | 0.184 | -0.212 |
| PC(34:0) | -0.211 | 0.141 | -0.107 |
| PC(37:7) | -0.297* | 0.112 | -0.18 |
| PC(37:6) | -0.4* | 0.247 | -0.267 |
| PC(36:1) | -0.253 | 0.137 | -0.099 |
| SM(d18:1/12:0) | 0.328* | -0.183 | 0.094 |
| SM(d18:1/24:0) | 0.242 | -0.227 | 0.178 |
| SM(d18:1/26:1(17Z)) | 0.106 | -0.191 | 0.064 |
| LPC(27:1) | 0.229 | -0.107 | 0.167 |
| LPC(27:0) | 0.145 | 0.027 | -0.007 |

*Correlation significantly different than zero.

*p<0.05; AHI: Apnoea-hypopnoea index; Cer: ceramide; DG: diacyglycerol; LPC: monoacylglycerophosphocholines (lyso-phosphocholines); O2: oxygen; PA: glycerophosphates; PC: glycerophosphocholines; PE: glycerophosphoethanolamines; sat: saturation; SM: sphingomyelin; TST: Total sleep time.
